# Rhodium-Catalyzed Oxidative Annulation of 2- or 7-Arylindoles with Alkenes/Alkynes Using Molecular Oxygen as the Sole Oxidant Enabled by Quaternary Ammonium Salt

**DOI:** 10.3390/molecules26175329

**Published:** 2021-09-02

**Authors:** Weihui Zhuang, Jiaqi Zhang, Yanping Zheng, Qiufeng Huang

**Affiliations:** 1 Fujian Key Laboratory of Polymer Materials, College of Chemistry & Materials Science, Fujian Normal University, Fuzhou 350007, China; zwh180652@163.com (W.Z.); jiaqi13206681102@163.com (J.Z.); zhengyanping1108@163.com (Y.Z.); 2 Fujian Provincial Key Laboratory of Advanced Materials Oriented Chemical Engineering, Fuzhou 350007, China

**Keywords:** rhodium catalysis, oxidative annulation, 2- or 7-arylindoles, molecular oxygen, quaternary ammonium salt

## Abstract

Developing an efficient catalytic system using molecular oxygen as the oxidant for rhodium-catalyzed cross-dehydrogenative coupling remains highly desirable. Herein, rhodium-catalyzed oxidative annulation of 2- or 7-phenyl-1*H*-indoles with alkenes or alkynes to assemble valuable 6*H*-isoindolo[2,1-*a*]indoles, pyrrolo[3,2,1-*de*]phenanthridines, or indolo[2,1-*a*]isoquinolines using the atmospheric pressure of air as the sole oxidant enabled by quaternary ammonium salt has been accomplished. Mechanistic studies provided evidence for the fast intramolecular aza-Michael reaction and aerobic reoxidation of Rh(I)/Rh(III), facilitated by the addition of quaternary ammonium salt.

## 1. Introduction

**Scheme 1:**
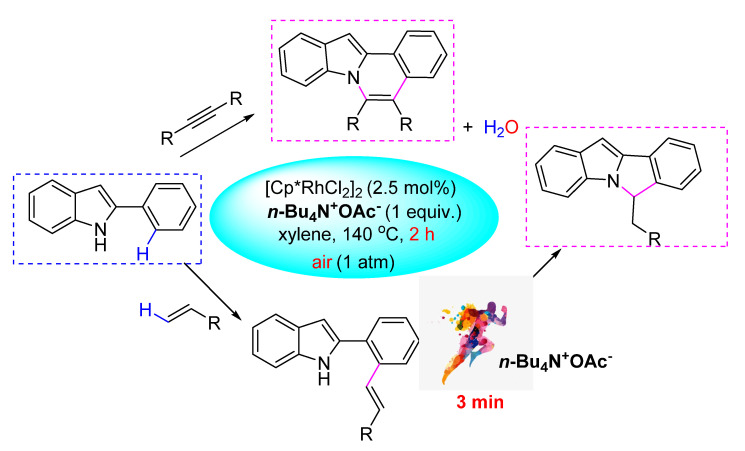
Rhodium-catalyzed oxidative annulation of 2- or 7-phenyl-1*H*-indoles with alkenes or alkynes using molecular oxygen as the sole oxidant enabled by quaternary ammonium salt.

C-H functionalization, including the reaction of a C-H bond with a (pseudo)halide, the reaction of a C-H bond with an organometallic reagent, and cross-coupling between two C-H bonds (CDC reaction), has gained tremendous popularity in recent years as a methodology for the construction of C-C bonds or C-heteroatom bonds [1,2,3,4,5,6,7,8,9,10,11,12]. Among these reactions, the CDC reaction is especially noteworthy because this reaction precludes both coupling partners from pre-functionalization, and as a result has high step economy and atom economy [13,14,15,16,17,18,19,20,21]. In 2007, Miura and Satoh reported on [RhCp*Cl_2_]_2_-catalyzed oxidative coupling of benzoic acids with alkynes [22]. Since then, rhodium-catalyzed oxidative C-H coupling has drawn increasing attention, and many important organic building blocks have been produced [23,24,25,26,27,28,29,30]. However, despite indisputable advances, all rhodium-catalyzed C-H oxidative coupling reactions are extremely limited to hazardous and stoichiometric oxidants such as AgOAc [31,32,33,34,35,36,37] and Cu(OAc)_2_ [38,39,40,41,42,43,44,45,46,47]. The use of molecular oxygen is advantageous over other oxidants because only water is generated as a by-product [48,49,50,51,52,53,54]. So far, in sharp contrast to aerobic palladium-catalyzed CDC reactions [55,56,57,58,59,60,61,62,63,64,65], only very limited examples of rhodium-catalyzed CDC reaction utilizing molecular oxygen as the sole oxidant have been reported to date [66,67,68,69,70]. Therefore, the development of protocols using molecular oxygen as the oxidant is highly desirable. In continuation of our research on transition metal-catalyzed aerobic CDC reactions [71,72,73], herein we report on the rhodium-catalyzed oxidative annulation of 2-arylindoles or 7-arylindoles with alkenes or alkynes using molecular oxygen as the sole oxidant enabled by quaternary ammonium salt (Scheme 1).

## 2. Results and Discussion

Our investigation on the aerobic rhodium-catalyzed CDC reaction began with the *NH*-indole-directed *ortho*-C-H alkenylation of 2-phenyl-1*H*-indole (**1a**) with *n*-butyl acrylate. The catalytic system consisting of [Cp*RhCl_2_]_2_ (2.5 mol%) and *n*-Bu_4_NOAc (1 equiv.) promoted the reaction at 140 °C under air atmosphere in xylenes to afford 6*H*-isoindolo[2,1-*a*]indole (**4a**) in 93% yield (Table 1, entry 2), derived from *ortho*-C-H olefination and the subsequent intramolecular aza-Michael addition. The addition of *n*-Bu_4_NOAc was indispensable as the reaction became very sluggish in its absence in various solvents such as xylenes, DMF, THF, EtOAc, and 1,4-dioxane (entry 1). A similar yield was obtained when Me_4_NOAc (1 equiv.) was added (entry 3), while other quaternary ammonium salts gave inferior results (entries 4–11). Control experiments have shown that no reaction occurred in the absence of rhodium catalyst or molecular oxygen (entry 12).

**Figure 1:**
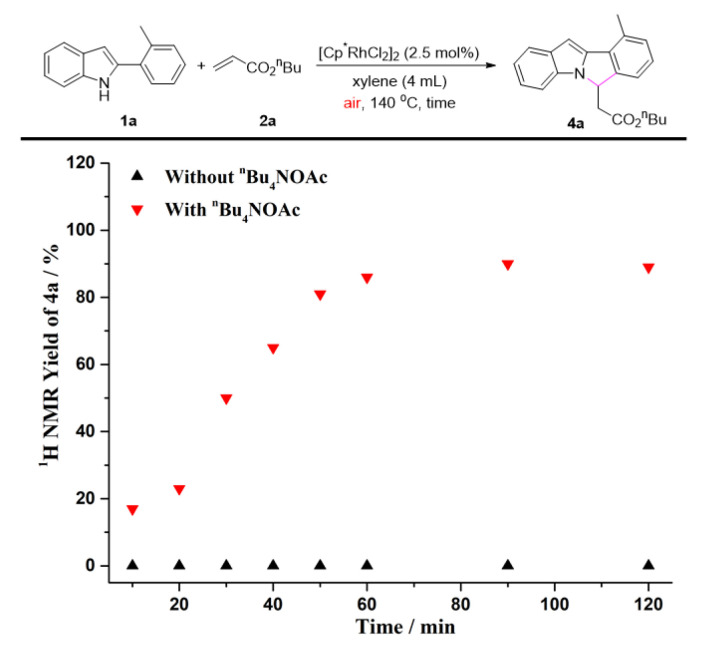
The one-pot C-H olefination/aza-Michael reaction of **1a** with **2a** under air.

**Figure 2:**
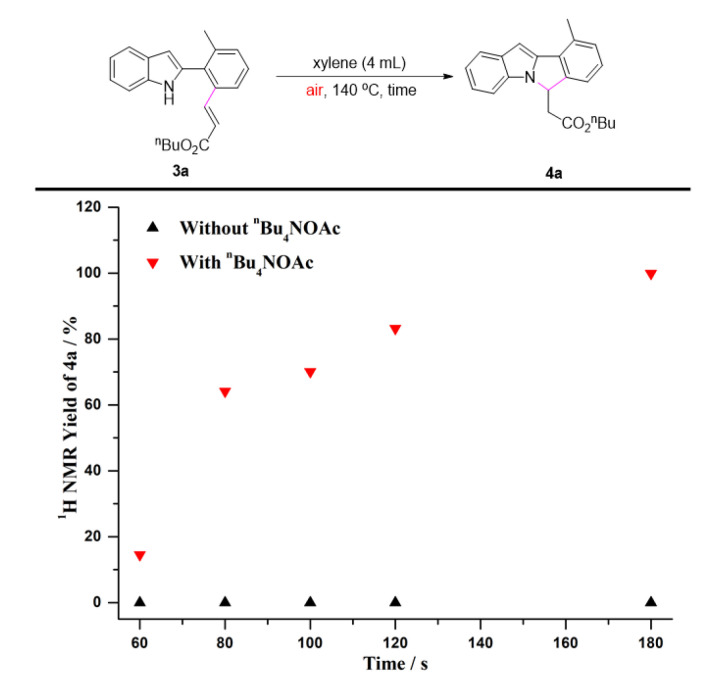
The intramolecular aza-Michael reaction of **3a** under air.

**Figure 3:**
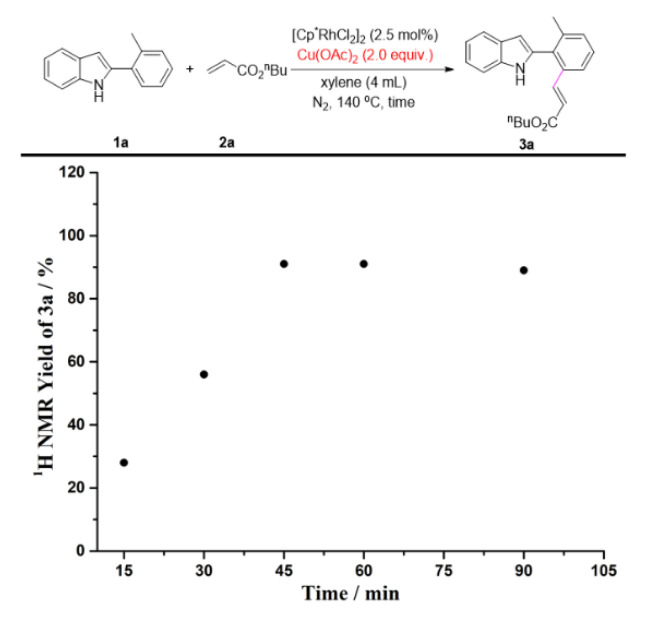
The C-H olefination of **1a** with **2a** using Cu(OAc)_2_ instead of O_2_ as the terminal oxidant.

**Figure 4:**
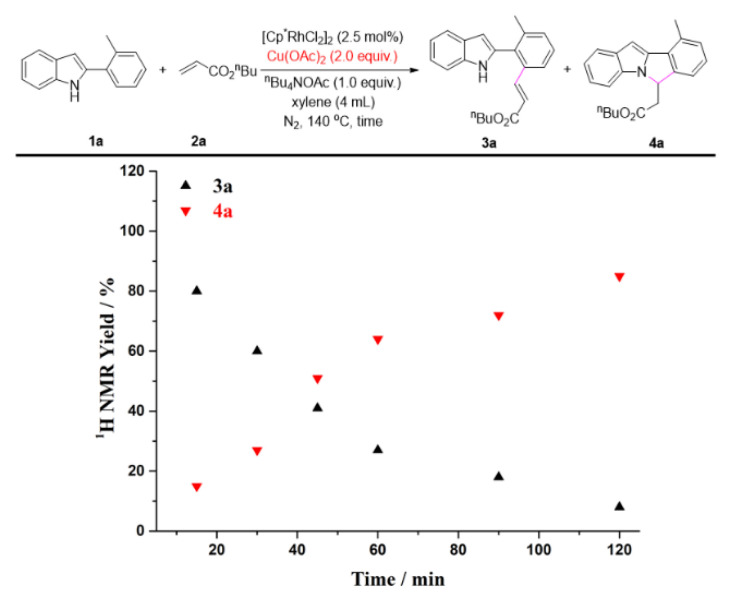
The C-H olefination/aza-Michael reaction of **1a** with **2a** using Cu(OAc)_2_ as the terminal oxidant.

**Figure 5:**
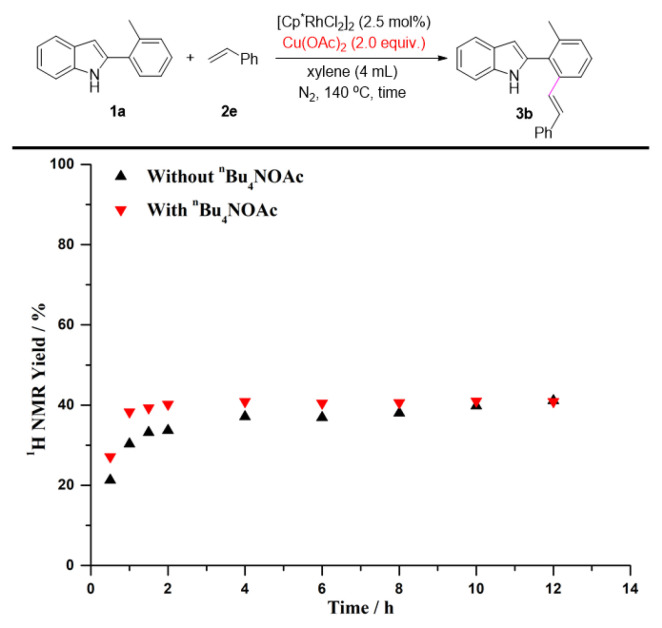
The C-H olefination of **1a** with **2e** using Cu(OAc)_2_ as the terminal oxidant.

To gain further insights into the impact of quaternary ammonium salts in the present transformation, we conducted several kinetic studies via ^1^H NMR spectroscopy. The time study shown in Figure 1 revealed that the one-pot C-H olefination/aza-Michael reaction under air atmosphere afforded 50% yield of **4a** after 30 min and was completed within 2 h by adding *n*-Bu_4_NOAc. It must be pointed out that the C-H olefinated product was not detected during monitoring period. Without *n*-Bu_4_NOAc, **4a** was not obtained at all, and nor was the C-H olefinated product (**3a**) formed. Quaternary ammonium salts have always been considered to be an effective catalyst for Michael reactions [74,75,76,77,78,79]. As one can see from Figure 2, the intramolecular aza-Michael reaction of *ortho*-alkenylated-2-phenyl-1*H*-indole could indeed be improved by the addition of *n*-Bu_4_NOAc. 1 equiv. of *n*-Bu_4_NOAc, and provided complete conversion and quantitative yield of **4a** after just 3 min. In the absence of *n*-Bu_4_NOAc, no reaction occurred, and the *ortho*-alkenylated-2-phenyl-1*H*-indole was totally recovered. The further kinetic experiments were carried out using Cu(OAc)_2_ instead of O_2_ as the terminal oxidant. As seen in Figure 3, the C-H olefination of 2-phenylindole with with *n*-butyl acrylate completed within 2 h in the absence of *n*-Bu_4_NOAc, affording 90% yield of **3a**. By adding *n*-Bu_4_NOAc, the C-H olefinated product (**3a**) was totally transformed into aza-Michael product **4a** within 2 h (Figure 4). In order to illustrate the impact of *n*-Bu_4_NOAc in the C-H olefination step, styrene was chosen as the coupling partner because it is not a Michael acceptor, and the reaction can stop after C-H olefination. As shown in Figure 5, no significant differences were observed between experiments performed with or without *n*-Bu_4_NOAc. These observations suggest that quaternary ammonium salt plays at least two roles in the oxidative annuation of 2-phenyl-1*H*-indole with with alkenes: (a) It promotes the intramolecular aza-Michael reaction of the C-H olefinated product; and (b) It promotes aerobic reoxidation of Rh(I) to Rh(III). The second role was partly validated by the fact that the current catalytic system ([Cp*RhCl_2_]_2_/*n*-Bu_4_NOAc/O_2_) was also effective for the oxidative annulation of 2-phenylindoles with alkynes to assemble indolo[2,1-*a*]isoquinoline skeletons. One reason why quaternary ammonium salt can speed up aerobic reoxidation is probably due to the increased dissolved quantity of O_2_ from adding quaternary ammonium salt [80,81,82].

Reaction condition: Figure 1. A solution of **1a** (0.2 mmol), **2a** (0.4 mmol), [Cp*RhCl_2_]_2_ (2.5 mol%), and *n*-Bu_4_NOAc (1.0 equiv.) in xylenes (4 mL) at 140 °C under air. Figure 2. A solution of **3a** (0.2 mmol) and *n*-Bu_4_NOAc (1.0 equiv.) in xylenes (4 mL) at 140 °C under air. Figure 3. A solution of **1a** (0.2 mmol), **2a** (0.4 mmol), [Cp*RhCl_2_]_2_ (2.5 mol%), and Cu(OAc)_2_ (2.0 equiv.) in xylenes (4 mL) at 140 °C under N_2_. Figure 4. A solution of **1a** (0.2 mmol), **2a** (0.4 mmol), [Cp*RhCl_2_]_2_ (2.5 mol%), *n*-Bu_4_NOAc (1.0 equiv.), and Cu(OAc)_2_ (2.0 equiv.) in xylenes (4 mL) at 140 °C under N_2_. Figure 5. A solution of **1a** (0.2 mmol), **2e** (0.4 mmol), [Cp*RhCl_2_]_2_ (2.5 mol%), *n*-Bu_4_NOAc (1.0 equiv.), and Cu(OAc)_2_ (2.0 equiv.) in xylenes (4 mL) at 140 °C under N_2_. The yields were determined by the ^1^H NMR yield using CH_2_Br_2_ (0.3 M, 0.2 mmol, 14 mg) as an internal standard.

**Scheme 2:**
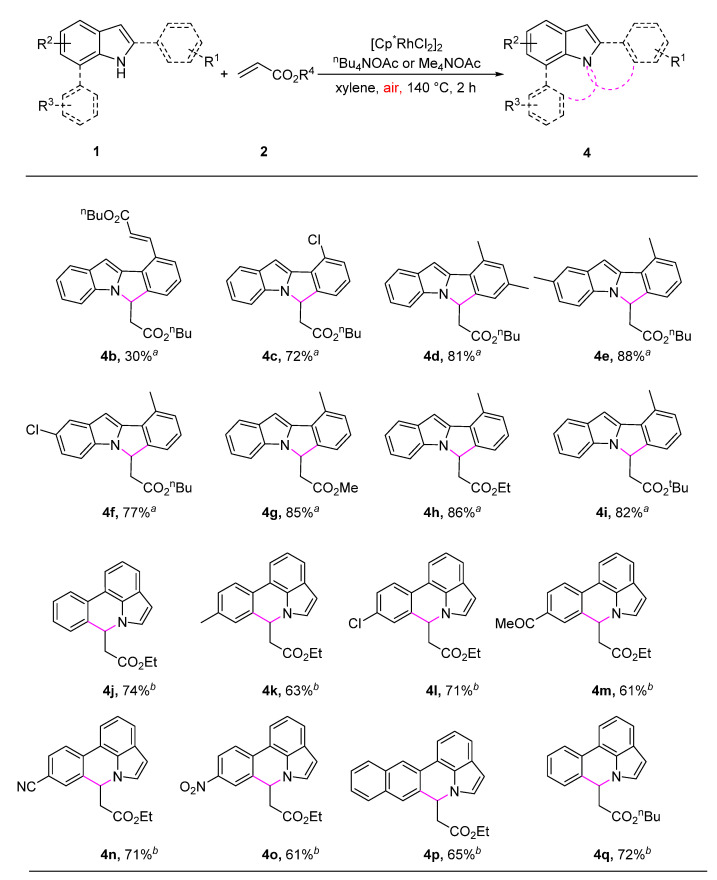
Substrate scope of oxidative annulation of 2- or 7-phenyl-1*H*-indoles with alkenes. *^a^*: Reaction condition: **1a** (0.2 mmol), **2** (0.4 mmol), [Cp*RhCl_2_]_2_ (2.5 mol%), and *n*-Bu_4_NOAc (1 equiv.) in xylenes (4 mL) at 140 °C under air atmosphere for 2 h. Isolated yield. *^b^*: Me_4_NOAc was used.

With the optimized conditions in hand, the generality of the rhodium-catalyzed aerobic C-H olefination/aza-Michael reaction was then explored (Scheme 2). The reaction of 2-phenyl-1*H*-indole, which contains two *ortho*-C-H bonds with *n*-butyl acrylate, provided the desired annulated product **4b** in low yield (30%) with recovered starting material (65%). Therefore, blocking one of the *ortho*-C-H bonds with methyl or chloro is essential for full conversion. 2-phenyl-1*H*-indole derivatives with substituents at the benzene ring or indole ring were delivered the corresponding products in good to excellent yields, showing very limited effect on the reaction efficiency (**4c**–**4f**). As expected, other acrylates bearing methyl, ethyl, or *tert*-butyl all well reacted with **1a** to afford the desired product **4g**–**4i** in good yields. The C-H olefination/aza-Michael reaction of 7-phenyl-1*H*-indoles with ethyl acrylate afforded the corresponding pyrrolo[3,2,1-*de*]phenanthridine derivatives under the reaction conditions by changing *n*-Bu_4_NOAc with Me_4_NOAc. By contrast, only one *ortho*-C-H bond was cleaved, showing good chemoselectivity (**4j**–**4q**). 7-phenylindoles and acrylates bearing various substituents, such as chloro (**4l**), ketone (**4m**), CN (**4n**), NO_2_ (**4o**), naphthyl (**4p**), and *n*-butyl (**4q**) coupled well with ethyl acrylate or ethyl acrylate, showing good functional group tolerance. The experiment results also showed no electronic effect on the reaction efficiency.

**Scheme 3:**
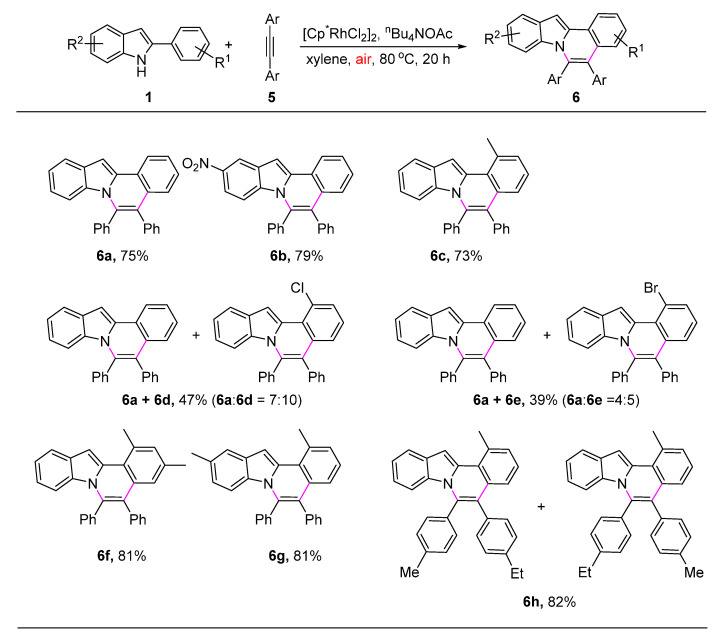
Substrate scope of oxidative annulation of 2-phenyl-1*H*-indoles with alkynes *^a^.* *^a^*: Reaction condition: **1a** (0.2 mmol), **5** (0.4 mmol), [Cp*RhCl_2_]_2_ (2.5 mol%), and *n*-Bu_4_NOAc (1 equiv.) in xylenes (4 mL) at 80 °C under air atmosphere for 20 h. Isolated yield.

Next, the scope of oxidative annulation of 2-phenyl-1*H*-indoles with alkynes was briefly investigated. As shown in Scheme 3, the reaction of 2-phenyl-*1H*-indoles **1** bearing an electron-rich or electron-deficient group at the phenyl ring or indole ring proceeded smoothly to give the corresponding products **6a**–**6c**, **6f**–**6g** in 39–81% yields. For 2-(2-chlorophenyl)-1*H*-indole or 2-(2-bromophenyl)-1*H*-indole, both C-H and C-Cl (or C-Br) cleavage occurred. The corresponding C-H oxidative annulation product is difficult to separate from the mixture (**6d** + **6a** or **6e** + **6a**). In the present [4 + 2] oxidative annulation, when an unsymmetrical diarylalkyne was employed, the formation of two possible regioisomers was observed as expected (**6h**). Again, valuable functional groups were well accommodated.

**Scheme 4:**
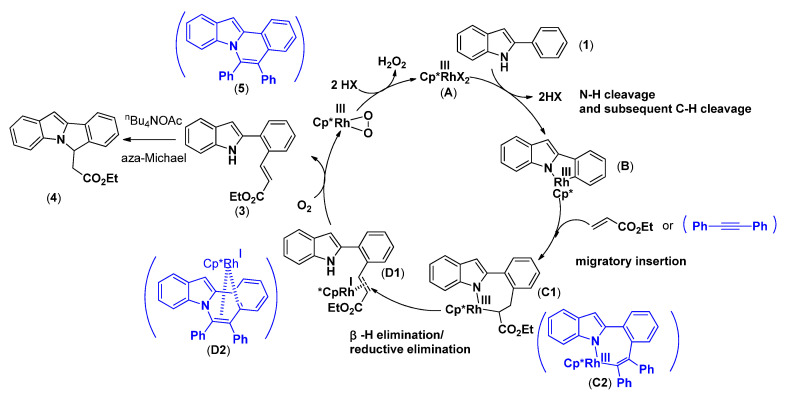
Plausible mechanism for the aerobic rhodium-catalyzed oxidative annulation of 2-phenylindole with alkene or alkyne.

Based on the experimental results obtained above and precedent reports [31,32,34,44], a plausible mechanism for the aerobic rhodium-catalyzed oxidative annulation of 2-phenylindole with alkene or alkyne is postulated in Scheme 4. Coordination of N atom of phenylindole to Rh(III) and the subsequent *ortho*-C-H activation produced the five-membered rhodacycle **B**. **B** inserted into the alkene or alkyne affording the intermediate **C1** or **C2**, and the subsequent β-H elimination/reductive elimination provided Rh(I) sandwich complex **D1** or **D2**. Then **D1** or **D2** was oxidized by oxygen to regenerate the active Rh(III) species and released the corresponding product **3** or **5**. The C-H olefinated product (**3**) can be transformed into aza-Michael product **4** efficiently, and the oxidation step by molecular oxygen will be sped up substantially by adding quaternary ammonium salts.

## 3. Materials and Methods

### 3.1. General Information

Unless otherwise noted, the reagents (chemicals) were purchased from commercial sources and were used without further purification. 2-phenyl-1*H*-indole is commercially available. The other 2-arylindoles were synthesized from phenylhydrazine hydrochlorides via Fisher indole synthesis [44]. 7-phenyl-1*H*-indoles were synthesized from 7-bromo-1*H*-indoles and phenylboronic acid via Suzuki coupling [34,35]. Quaternary ammonium salts were purchased from commercial sources. Their purity was more than 99.0% and they were stored in a glovebox. ^1^H NMR spectra were recorded at 400 MHz and ^13^C NMR spectra at 100 MHz, respectively (Supplementary material). ^1^H chemical shifts (δ) were referenced to TMS, and ^13^C NMR chemical shifts (δ) were referenced to internal solvent resonance. ESI-HRMS spectra were recorded by using a Q-TOF mass spectrometer.

### 3.2. General Procedure for Rhodium-Catalyzed Oxidative Annulation of 2- or 7-Arylindoles with Alkenes/Alkynes

Under air atmosphere, 2- or 7-arylindoles (0.2 mmol), alkenes or alkynes (0.4 mmol), [Cp*RhCl_2_]_2_ (3.2 mg, 0.005 mmol, 2.5 mol%), *n*-Bu_4_NOAc or Me_4_NOAc (0.2 mmol, 1 equiv.), and xylenes (4 mL) were placed in a 25 mL tube. The mixture was heated in oil bath at 140 °C for 2 h or 80 °C for 20 h. After the reaction mixture cooled to room temperature, the crude reaction mixture was diluted with EtOAc to 5 mL, filtered through a celite pad, and then washed with 10 mL EtOAc. The combined mixture was washed with saturated aqueous Na_2_CO_3_ and dried over anhydrous MgSO_4_. After filtration, the volatiles were removed under reduced pressure, and the residue was subjected to silica gel column chromatography (eluting with petroleum ether/dichloromethane = 1/1 or petroleum ether/ethyl acetate = 100/1) to afford the corresponding product.

### 3.3. Analytical Characterization Data of Products

*Butyl 3-(2-(1H-indol-2-yl)-3-methylphenyl)acrylate* (**3a**), 57.3 mg, 85% yield, yellow oil. ^1^H NMR (400 MHz, CDCl_3_) δ 8.22 (s, 1H), 7.68 (d, *J* = 7.6 Hz, 1H), 7.60–7.55 (m, 2H), 7.39–7.32 (m, 3H), 7.24–7.15 (m, 2H), 6.48 (dd, *J* = 2.0, 1.2 Hz, 1H), 6.33 (d, *J* = 16.0 Hz, 1H), 4.06 (t, *J* = 6.4 Hz, 2H), 2.23 (s, 3H), 1.59–1.51 (m, 2H), 1.31–1.25 (m, 2H), 0.86 (t, *J* = 7.2 Hz, 3H). ^13^C NMR and HRMS data for the desired product were in agreement with the previously reported literature data [44].

*2-(2-Methyl-6-styrylphenyl)-1H-indole* (**3b**), 25.3 mg, 41% yield, yellow oil. ^1^H NMR (400 MHz, CDCl_3_) δ 7.98 (s, 1H), 7.73–7.71 (m, 1H), 7.67 (d, *J* = 8.0 Hz, 1H), 7.42–7.36 (m, 2H), 7.32–7.25 (m, 4H), 7.24–7.18 (m, 4H), 7.03 (d, *J* = 8.4 Hz, 2H), 6.54 (dd, *J* = 2.0, 0.8 Hz, 1H), 2.26 (s, 3H). ^13^C NMR and HRMS data for the desired product were in agreement with the previously reported literature data [44].

*Butyl 2-(10-methyl-6H-isoindolo[2,1-a]indol-6-yl)acetate* (**4a**), 61.4 mg, 93% yield, yellow oil. ^1^H NMR (400 MHz, CDCl_3_) δ 7.69 (d, *J* = 8.0 Hz, 1H), 7.40 (dd, *J* = 8.4, 0.8 Hz, 1H), 7.30 (t, *J* = 4.4 Hz, 1H), 7.23–7.19 (m, 3H), 7.13 (td, *J* = 8.0, 0.8 Hz, 1H), 6.61 (s, 1H), 5.75 (dd, *J* = 8.0, 4.4 Hz, 1H), 4.21–4.14 (m, 2H), 3.30 (dd, *J* = 16.4, 4.8 Hz, 1H), 2.76 (dd, *J* = 16.4, 8.0 Hz, 1H), 2.63 (s, 3H), 1.62–1.55 (m, 2H), 1.35–1.30 (m, 2H), 0.92 (t, *J* = 7.2 Hz, 3H). ^13^C NMR (100 MHz, CDCl_3_) δ 171.1, 145.8, 143.1, 133.5, 133.2, 132.5, 131.3, 129.9, 127.5, 121.9, 120.7, 119.8, 109.6, 94.2, 65.2, 56.8, 39.9, 30.6, 19.5, 19.2, 13.8. HRMS (ESI) calcd for C_22_H_24_NO_2_ [M + H]^+^: 334.1807, found: 334.1808.

*Butyl 3-(6-(2-butoxy-2-oxoethyl)-6H-isoindolo[2,1-a]indol-10-yl)acrylate* (**4b**), 29.7 mg, 30% yield, red solid. ^1^H NMR (400 MHz, CDCl_3_) δ 8.32 (d, *J* = 16.0 Hz, 1H), 7.70 (dt, *J* = 8.0, 0.8 Hz, 1H), 7.64 (d, *J* = 7.6 Hz, 1H), 7.46 (dt, *J* = 7.2, 1.2 Hz, 1H), 7.39 (dd, *J* = 8.0, 0.8 Hz, 1H), 7.30 (t, *J* = 7.6 Hz, 1H), 7.23 (td, *J* = 7.2, 1.2 Hz, 1H), 7.16–7.12 (m, 1H), 6.79 (s, 1H), 6.56 (d, *J* = 15.6 Hz, 1H), 5.74 (dd, *J* = 8.0, 4.4 Hz, 1H), 4.29 (t, *J* = 6.8 Hz, 2H), 4.19–4.12 (m, 2H), 3.31 (dd, *J* = 16.4, 4.4 Hz, 1H), 2.77 (dd, *J* = 16.0, 8.0 Hz, 1H), 1.80–1.73 (m, 2H), 1.56–1.48 (m, 4H), 1.33–1.26 (m, 2H), 1.02 (t, *J* = 7.6 Hz, 3H), 0.90 (t, *J* = 7.6 Hz, 3H). ^13^C NMR and HRMS data for the desired product were in agreement with the previously reported literature data [44].

*Butyl 2-(10-chloro-6H-isoindolo[2,1-a]indol-6-yl)acetate* (**4c**), 50.6 mg, 72% yield, yellow oil. ^1^H NMR (400 MHz, CDCl_3_) δ 7.71 (dt, *J* = 8.0, 0.8 Hz, 1H), 7.40–7.36 (m, 3H), 7.23 (td, *J* = 8.0, 1.2 Hz, 2H), 7.16–7.12 (m, 1H), 6.91 (s, 1H), 5.77 (dd, *J* = 8.0, 4.4 Hz, 1H), 4.19–4.13 (m, 2H), 3.31 (dd, *J* = 16.4, 4.4 Hz, 1H), 2.77 (dd, *J* = 16.4, 8.4 Hz, 1H), 1.59–1.55 (m, 2H), 1.34–1.28 (m, 2H), 0.91 (t, *J* = 7.6 Hz, 3H). ^13^C NMR (100 MHz, CDCl_3_) δ 170.7, 147.4, 140.6, 133.3, 133.2, 131.0, 129.4, 128.3, 128.0, 122.5, 121.8, 120.1, 109.6, 96.0, 65.3, 57.0, 39.6, 30.6, 19.2, 13.8. HRMS (ESI) calcd for C_21_H_21_NO_2_Cl [M + H]^+^: 354.1261, found: 354.1257.

*Butyl 2-(8,10-dimethyl-6H-isoindolo[2,1-a]indol-6-yl)acetate* (**4d**), 56.2 mg, 81% yield, yellow oil. ^1^H NMR (400 MHz, CDCl_3_) δ 7.69 (dt, *J* = 7.6, 1.2 Hz, 1H), 7.39 (d, *J* = 8.0 Hz, 1H), 7.23–7.19 (m, 1H), 7.15–7.11 (m, 2H), 7.05 (s, 1H), 6.56 (s, 1H), 5.70 (dd, *J* = 8.0, 4.4 Hz, 1H), 4.25–4.16 (m, 2H), 3.29 (dd, *J* = 16.0, 4.4 Hz, 1H), 2.76 (dd, *J* = 16.0, 8.0 Hz, 1H), 2.59 (s, 3H), 2.40 (s, 3H), 1.63–1.59 (m, 2H), 1.38–1.33 (m, 2H), 0.94 (t, *J* = 7.6 Hz, 3H). ^13^C NMR (100 MHz, CDCl_3_) δ 171.1, 146.1, 143.2, 137.6, 133.6, 133.1, 132.2, 130.8, 128.6, 121.7, 121.6, 121.4, 119.6, 109.4, 93.4, 65.1, 56.7, 39.9, 30.7, 21.7, 19.4, 19.2, 13.8. HRMS (ESI) calcd for C_23_H_26_NO_2_ [M + H]^+^: 348.1964, found: 348.1960.

*Butyl 2-(2,10-dimethyl-6H-isoindolo[2,1-a]indol-6-yl)acetate* (**4e**), 60.7 mg, 88% yield, yellow oil. ^1^H NMR (400 MHz, CDCl_3_) δ 7.47 (s, 1H), 7.30–7.27 (m, 2H), 7.22–7.20 (m, 2H), 7.04 (dd, *J* = 8.4, 1.2 Hz, 1H), 6.52 (s, 1H), 5.72 (dd, *J* = 8.0, 4.8 Hz, 1H), 4.21–4.14 (m, 2H), 3.27 (dd, *J* = 16.4, 4.8 Hz, 1H), 2.74 (dd, *J* = 16.0, 8.0 Hz, 1H), 2.62 (s, 3H), 2.47 (s, 3H), 1.61–1.57 (m, 2H), 1.36–1.30 (m, 2H), 0.92 (t, *J* = 7.6 Hz, 3H). ^13^C NMR (100 MHz, CDCl_3_) δ 171.1, 145.8, 143.1, 133.8, 132.4, 131.6, 131.5, 129.9, 129.0, 127.3, 123.5, 121.6, 120.7, 109.2, 93.7, 65.1, 56.8, 39.9, 30.7, 21.6, 19.5, 19.2, 13.8. HRMS (ESI) calcd for C_23_H_26_NO_2_ [M + H]^+^: 348.1964, found: 348.1964.

*Butyl 2-(2-chloro-10-methyl-6H-isoindolo[2,1-a]indol-6-yl)acetate* (**4f**), 57.2 mg, 77% yield, yellow oil. ^1^H NMR (400 MHz, CDCl_3_) δ 7.63 (dd, *J* = 2.0, 0.4 Hz, 1H), 7.31–7.28 (m, 2H), 7.23–7.22 (m, 2H), 7.14 (dd, *J* = 8.4, 2.0 Hz, 1H), 6.53 (s, 1H), 5.71 (dd, *J* = 7.6, 4.8 Hz, 1H), 4.18–4.12 (m, 2H), 3.19 (dd, *J* = 16.4, 4.8 Hz, 1H), 2.79 (dd, *J* = 16.0, 7.6 Hz, 1H), 2.61 (s, 3H), 1.58–1.54 (m, 2H), 1.33–1.27 (m, 2H), 0.91 (t, *J* = 7.2 Hz, 3H). ^13^C NMR (100 MHz, CDCl_3_) δ 170.8, 145.7, 144.4, 134.5, 132.7, 131.6, 130.9, 130.1, 127.9, 125.4, 122.0, 121.1, 120.7, 110.4, 93.8, 65.2, 57.1, 39.9, 30.6, 19.5, 19.2, 13.8. HRMS (ESI) calcd for C_22_H_23_NO_2_Cl [M + H]^+^: 368.1417, found: 368.1412.

*Methyl 2-(10-methyl-6H-isoindolo[2,1-a]indol-6-yl)acetate* (**4g**), 49.4 mg, 85% yield, yellow oil. ^1^H NMR (400 MHz, CDCl_3_) δ 7.74 (dd, *J* = 7.6, 0.8 Hz, 1H), 7.41 (d, *J* = 8.0 Hz, 1H), 7.33–7.30 (m, 1H), 7.28–7.24 (m, 3H), 7.19–7.15 (m, 1H), 6.64 (s, 1H), 5.74 (dd, *J* = 8.4, 4.8 Hz, 1H), 3.82 (s, 3H), 3.32 (dd, *J* = 16.4, 4.8 Hz, 1H), 2.73 (dd, *J* = 16.0, 8.0 Hz, 1H), 2.64 (s, 3H). ^13^C NMR (100 MHz, CDCl_3_) δ 171.4, 145.7, 142.9, 133.5, 133.1, 132.5, 131.1, 129.9, 127.4, 121.8, 120.7, 119.7, 109.5, 94.2, 56.7, 52.2, 39.6, 19.5. HRMS (ESI) calcd for C_19_H_18_NO_2_ [M + H]^+^: 292.1338, found: 292.1340.

*Ethyl 2-(10-methyl-6H-isoindolo[2,1-a]indol-6-yl)acetate* (**4h**), 51.9 mg, 86% yield, yellow oil. ^1^H NMR (400 MHz, CDCl_3_) δ 7.69 (dt, *J* = 7.6, 1.2 Hz, 1H), 7.40 (dq, *J* = 8.0, 0.8 Hz, 1H), 7.32–7.29 (m, 1H), 7.23–7.19 (m, 3H), 7.14–7.10 (m, 1H), 6.61 (s, 1H), 5.75 (dd, *J* = 8.0, 4.4 Hz, 1H), 4.24 (qd, *J* = 7.2, 2.4 Hz, 2H), 3.29 (dd, *J* = 16.4, 4.8 Hz, 1H), 2.74 (dd, *J* = 16.0, 8.0 Hz, 1H), 2.62 (s, 3H), 1.25 (t, *J* = 6.8 Hz, 3H). ^13^C NMR (100 MHz, CDCl_3_) δ 171.0, 145.8, 143.1, 133.5, 133.2, 132.5, 131.3, 129.9, 127.5, 121.9, 120.8, 119.8, 109.6, 94.2, 61.2, 56.8, 39.9, 19.5, 14.2. HRMS (ESI) calcd for C_20_H_20_NO_2_ [M + H]^+^: 306.1494, found: 306.1493.

*Tert-butyl 2-(10-methyl-6H-isoindolo[2,1-a]indol-6-yl)acetate* (**4i**), 53.6 mg, 81% yield, yellow oil. ^1^H NMR (400 MHz, CDCl_3_) δ 7.69 (dt, *J* = 8.0, 0.8 Hz, 1H), 7.44 (dq, *J* = 8.0, 0.8 Hz, 1H), 7.35–7.32 (m, 1H), 7.24–7.19 (m, 3H), 7.15–7.10 (m, 1H), 6.61 (s, 1H), 5.71 (dd, *J* = 7.6, 4.4 Hz, 1H), 3.21 (dd, *J* = 16.0, 4.4 Hz, 1H), 2.77 (dd, *J* = 16.0, 7.6 Hz, 1H), 2.63 (s, 3H), 1.39 (s, 9H). ^13^C NMR (100 MHz, CDCl_3_) δ 170.0, 145.9, 143.2, 133.5, 133.2, 132.4, 131.4, 129.8, 127.4, 121.8, 120.8, 119.7, 109.7, 105.1, 94.0, 81.6, 57.0, 40.8, 28.0, 19.5. HRMS (ESI) calcd for C_22_H_24_NO_2_ [M + H]^+^: 334.1807, found: 334.1804.

*Ethyl 2-(7H-pyrrolo[3,2,1-de]phenanthridin-7-yl)acetate* (**4j**), 43.5 mg, 74% yield, yellow oil. ^1^H NMR (400 MHz, CDCl_3_) δ 7.97 (d, *J* = 7.6 Hz, 1H), 7.59 (d, *J* = 7.6 Hz, 1H), 7.55 (dd, *J* = 7.6, 0.4 Hz, 1H), 7.41–7.37 (m, 1H), 7.32–7.30 (m, 2H), 7.25 (d, *J* = 3.2 Hz, 1H), 7.16 (t, *J* = 7.6 Hz, 1H), 6.56 (d, *J* = 3.2 Hz, 1H), 6.14 (dd, *J* = 7.2, 5.2 Hz, 1H), 4.19–4.03 (m, 2H), 2.77 (dd, *J* = 7.2, 4.8 Hz, 2H), 1.16 (t, *J* = 7.2 Hz, 3H). ^13^C NMR and HRMS data for the desired product were in agreement with the previously reported literature data [35].

*Ethyl 2-(9-methyl-7H-pyrrolo[3,2,1-de]phenanthridin-7-yl)acetate* (**4k**), 39.1 mg, 63% yield, yellow soild. ^1^H NMR (400 MHz, CDCl_3_) δ 7.85 (d, *J* = 7.6 Hz, 1H), 7.54 (d, *J* = 7.2 Hz, 1H), 7.51 (dd, *J* = 8.0, 0.8 Hz, 1H), 7.23 (d, *J* = 3.2 Hz, 1H), 7.21–7.11 (m, 3H), 6.54 (d, *J* = 3.2 Hz, 1H), 6.08 (dd, *J* = 6.8, 5.6 Hz, 1H), 4.15–4.07 (m, 2H), 2.76 (d, *J* = 1.6 Hz, 1H), 2.75 (s, 1H), 2.38 (s, 3H), 1.16 (t, *J* = 7.2 Hz, 3H). ^13^C NMR and HRMS data for the desired product were in agreement with the previously reported literature data [35].

*Ethyl 2-(9-chloro-7H-pyrrolo[3,2,1-de]phenanthridin-7-yl)acetate* (**4l**), 48.4 mg, 71% yield, yellow oil. ^1^H NMR (400 MHz, CDCl_3_) δ 7.87 (d, *J* = 8.4 Hz, 1H), 7.55–7.52 (m, 2H), 7.35 (dd, *J* = 8.4, 2.0 Hz, 1H), 7.30 (d, *J* = 2.0 Hz, 1H), 7.23 (d, *J* = 3.2 Hz, 1H), 7.14 (t, *J* = 7.6 Hz, 1H), 6.56 (d, *J* = 3.2 Hz, 1H), 6.08 (dd, *J* = 6.8, 5.2 Hz, 1H), 4.11 (q, *J* = 7.2 Hz, 2H), 2.76 (dd, *J* = 7.2, 4.8 Hz, 2H), 1.17 (t, *J* = 6.8 Hz, 3H). ^13^C NMR and HRMS data for the desired product were in agreement with the previously reported literature data [35].

*Ethyl 2-(9-acetyl-7H-pyrrolo[3,2,1-de]phenanthridin-7-yl)acetate* (**4m**), 40.9 mg, 61% yield, yellow oil. ^1^H NMR (400 MHz, CDCl_3_) δ 8.00–7.91 (m, 3H), 7.60 (t, *J* = 6.4 Hz, 2H), 7.25 (s, 1H), 7.16 (t, *J* = 8.0 Hz, 1H), 6.57 (d, *J* = 3.2 Hz, 1H), 6.15 (dd, *J* = 7.6, 5.2 Hz, 1H), 4.14–4.05 (m, 2H), 2.82–2.74 (m, 2H), 2.62 (s, 3H), 1.15 (t, *J* = 7.2 Hz, 3H). ^13^C NMR (100 MHz, CDCl_3_) δ 197.1, 170.4, 136.2, 134.9, 134.0, 133.3, 128.6, 127.7, 127.1, 126.4, 122.9, 122.4, 120.9, 117.0, 115.1, 103.8, 61.2, 55.4, 46.4, 26.7, 14.1. HRMS (ESI) calcd for C_21_H_20_NO_3_ [M + H]^+^: 334.1443, found: 334.1446.

*Ethyl 2-(9-cyano-7H-pyrrolo[3,2,1-de]phenanthridin-7-yl)acetate* (**4n**), 44.7 mg, 71% yield, yellow solid. ^1^H NMR (400 MHz, CDCl_3_) δ 8.02 (d, *J* = 8.0 Hz, 1H), 7.66 (dd, *J* = 8.0, 1.6 Hz, 1H), 7.63–7.58 (m, 3H), 7.25 (d, *J* = 3.2 Hz, 1H), 7.18 (t, *J* = 7.6 Hz, 1H), 6.59 (d, *J* = 3.2 Hz, 1H), 6.13 (dd, *J* = 7.6, 5.2 Hz, 1H), 4.10 (q, *J* = 7.2 Hz, 2H), 2.77 (qd, *J* = 16.0, 7.6 Hz, 2H), 1.16 (t, *J* = 7.2 Hz, 3H). ^13^C NMR and HRMS data for the desired product were in agreement with the previously reported literature data [35].

*Ethyl 2-(9-nitro-7H-pyrrolo[3,2,1-de]phenanthridin-7-yl)acetate* (**4o**), 41.3 mg, 61% yield, yellow oil. ^1^H NMR (400 MHz, CDCl_3_) δ 8.24 (dd, *J* = 8.8, 2.4 Hz, 1H), 8.20 (d, *J* = 2.0 Hz, 1H), 8.06 (d, *J* = 8.4 Hz, 1H), 7.64 (d, *J* = 3.2 Hz, 1H), 7.62 (d, *J* = 2.4 Hz, 1H), 7.27 (d, *J* = 3.2 Hz, 1H), 7.19 (t, *J* = 7.6 Hz, 1H), 6.60 (d, *J* = 3.2 Hz, 1H), 6.20 (dd, *J* = 7.2, 4.8 Hz, 1H), 4.10 (qd, *J* = 7.2, 2.0 Hz, 2H), 2.82 (qd, *J* = 16.0, 7.2 Hz, 2H), 1.16 (t, *J* = 7.2 Hz, 3H). ^13^C NMR and HRMS data for the desired product were in agreement with the previously reported literature data [35].

*Ethyl 2-(7H-benzo[j]pyrrolo[3,2,1-de]phenanthridin-7-yl)acetate* (**4p**), 44.5 mg, 65% yield, yellow solid. ^1^H NMR (400 MHz, CDCl_3_) δ 8.40 (s, 1H), 7.90 (d, *J* = 7.6 Hz, 1H), 7.80 (s, 1H), 7.78 (d, *J* = 2.8 Hz, 2H), 7.59 (dd, *J* = 8.0, 0.8 Hz, 1H), 7.55–7.44 (m, 2H), 7.30 (d, *J* = 3.2 Hz, 1H), 7.23 (t, *J* = 7.6 Hz, 1H), 6.60 (d, *J* = 3.2 Hz, 1H), 6.26 (t, *J* = 6.4 Hz, 1H), 4.14–4.06 (m, 2H), 2.82 (qd, *J* = 15.6, 7.2 Hz, 2H), 1.14 (t, *J* = 7.2 Hz, 3H). ^13^C NMR and HRMS data for the desired product were in agreement with the previously reported literature data [35].

*Butyl 2-(7H-pyrrolo[3,2,1-de]phenanthridin-7-yl)acetate* (**4q**), 47.5 mg, 72% yield, yellow oil. ^1^H NMR (400 MHz, CDCl_3_) δ 7.97 (d, *J* = 7.6 Hz, 1H), 7.58 (d, *J* = 7.6 Hz, 1H), 7.54 (dd, *J* = 8.0, 0.8 Hz, 1H), 7.41–7.37 (m, 1H), 7.32–7.30 (m, 2H), 7.24 (d, *J* = 3.2 Hz, 1H), 7.16 (t, *J* = 7.6 Hz, 1H), 6.55 (d, *J* = 3.2 Hz, 1H), 6.14 (dd, *J* = 7.2, 5.2 Hz, 1H), 4.11–4.00 (m, 2H), 2.78 (qd, *J* = 16.0, 7.6 Hz, 2H), 1.54–1.47 (m, 2H), 1.30–1.24 (m, 2H), 0.89 (t, *J* = 7.6 Hz, 3H). ^13^C NMR and HRMS data for the desired product were in agreement with the previously reported literature data [35].

*5,6-Diphenylindolo[2,1-a]isoquinoline* (**6a**), 55.8 mg, 75% yield, yellow solid. ^1^H NMR (400 MHz, CDCl_3_) δ 8.31 (d, *J* = 8.0 Hz, 1H), 7.81 (d, *J* = 8.0 Hz, 1H), 7.52 (t, *J* = 7.6 Hz, 1H), 7.43 (s, 1H), 7.41–7.25 (m, 7H), 7.26–7.13 (m, 6H), 6.83 (t, *J* = 8.0 Hz, 1H), 6.01 (d, *J* = 8.8 Hz, 1H). ^13^C NMR (100 MHz, CDCl_3_) δ 136.9, 136.1, 135.5, 132.9, 132.0, 131.0, 130.4, 129.8, 128.8, 128.7, 128.0, 127.5, 127.2, 126.9, 126.3, 125.5, 123.4, 121.8, 120.3, 120.2, 114.7, 94.3. HRMS data for the desired product were in agreement with the previously reported literature data [40].

*10-Nitro-5,6-diphenylindolo[2,1-a]isoquinoline* (**6b**), 64.8 mg, 79% yield, orange solid. ^1^H NMR (400 MHz, CDCl_3_) δ 8.70 (d, *J* = 2.4 Hz, 1H), 8.32 (d, *J* = 8.0 Hz, 1H), 7.66 (dd, *J* = 9.6, 2.4 Hz, 1H), 7.59–7.54 (m, 2H), 7.44–7.36 (m, 4H), 7.31–7.27 (m, 3H), 7.25–7.17 (m, 5H), 5.99 (d, *J* = 9.6 Hz, 1H). ^13^C NMR and HRMS data for the desired product were in agreement with the previously reported literature data [40].

*1-Methyl-5,6-diphenylindolo[2,1-a]isoquinoline* (**6c**), 55.3 mg, 73% yield, yellow solid, m.p. 173.7–174.0 °C. ^1^H NMR (400 MHz, CDCl_3_) δ 7.84 (d, *J* = 8.0 Hz, 1H), 7.56 (s, 1H), 7.39 (d, *J* = 7.6 Hz, 1H), 7.36–7.26 (m, 6H), 7.25–7.16 (m, 6H), 7.04 (d, *J* = 8.0 Hz, 1H), 6.86–6.81 (m, 1H), 6.00 (d, *J* = 8.4 Hz, 1H), 3.03 (s, 3H). ^13^C NMR (100 MHz, CDCl_3_) δ 137.6, 135.9, 135.2, 132.0, 131.9, 131.0, 130.3, 129.6, 128.7, 128.0, 126.8, 126.6, 125.1, 124.5, 121.6, 120.6, 120.5, 114.8, 100.7, 25.4. HRMS (ESI) calcd for C_29_H_22_N [M + H]^+^: 384.1752, found: 384.1751.

*1-Chloro-5,6-diphenylindolo[2,1-a]isoquinoline* (**6d**) and *5,6-diphenylindolo[2,1-a]isoquinoline* (**6a**), 37.5 mg, 47% yield, yellow solid, m.p. 201.1–201.6 °C. ^1^H NMR (400 MHz, CDCl_3_) δ 8.40 (**6d**, d, *J* = 0.8 Hz, 1H), 8.30 (**6a**, dt, *J* = 8.0, 0.8 Hz, 1H), 7.85 (**6d**, dt, *J* = 8.0, 1.2 Hz, 1H), 7.79 (**6a**, dt, *J* = 8.0, 1.2 Hz, 1H), 7.57 (**6d**, dd, *J* = 8.0, 1.2 Hz, 1H), 7.53–7.49 (**6a**, m, 1H), 7.42 (**6a**, d, *J* = 0.4 Hz, 1H), 7.36–7.28 (**6d** + **6a**, m, 11H), 7,24–7.13 (**6d** + **6a**, m, 14H), 7.05 (**6d**, dd, *J* = 8.0, 1.2 Hz, 1H), 6.87–6.79 (**6d** + **6a**, m, 2H), 6.00–5.95 (**6d** + **6a**, m, 2H). ^13^C NMR (100 MHz, CDCl_3_) δ 137.0, 135.4, 132.9, 132.0, 131.0, 130.8, 129.7, 128.9, 128.8, 128.7, 128.1, 128.0, 127.5, 127.2, 127.1, 126.9, 126.3, 125.1, 123.6, 123.4, 121.8, 121.8, 121.3, 121.1, 120.2, 114.7, 102.5, 94.3. HRMS (ESI) calcd for **6d** C_29_H_19_NCl [M + H]^+^: 404.1206, found: 404.1209.

*1-Bromo-5,6-diphenylindolo[2,1-a]isoquinoline* (**6e**) and *5,6-diphenylindolo[2,1-a]isoquinoline* (**6a**), 34.9 mg, 39% yield, yellow solid, m.p. 188.5–188.9 °C. ^1^H NMR (400 MHz, CDCl_3_) δ 8.67 (**6e**, s, 1H), 8.31 (**6a**, d, *J* = 8.0 Hz, 1H), 7.85 (**6e**, d, *J* = 8.0 Hz, 1H), 7.82–7.79 (**6e** + **6a**, m, 2H), 7.53–7.49 (**6a**, m, 1H), 7.43–7.27 (**6e** + **6a**, m, 13H), 7.25–7.10 (**6e** + **6a**, m, 14H), 6.87–6.79 (**6e** + **6a**, m, 2H), 6.00 (**6a**, d, *J* = 8.8 Hz, 1H), 5.97 (**6e**, d, *J* = 8.8 Hz, 1H). **^13^C NMR** (100 MHz, CDCl_3_) δ 133.7, 132.0, 132.0, 131.0, 130.8, 128.9, 128.9, 128.8, 128.8, 128.2, 128.0, 127.5, 127.3, 127.2, 127.1, 127.1, 126.9, 126.3, 125.9, 121.3, 121.1, 120.3, 120.2, 119.7, 114.7, 102.2, 94.3. HRMS (ESI) calcd for **6e** C_29_H_19_NBr [M + H]^+^: 448.0701, found: 448.0705.

*1,3-Dimethyl-5,6-diphenylindolo[2,1-a]isoquinoline* (**6f**), 64.4 mg, 81% yield, orange solid, m.p. 183.2–183.7 °C. ^1^H NMR (400 MHz, CDCl_3_) δ 7.82 (dt, *J* = 8.0, 1.2 Hz, 1H), 7.50 (s, 1H), 7.35–7.27 (m, 5H), 7.25–7.15 (m, 7H), 6.84–6.79 (m, 2H), 5.98 (d, *J* = 8.4 Hz, 1H), 2.99 (s, 3H), 2.31 (s, 3H). ^13^C NMR (100 MHz, CDCl_3_) δ 137.7, 136.5, 136.0, 135.9, 135.7, 135.1, 132.1, 131.9, 131.7, 131.0, 129.7, 128.7, 128.7, 128.0, 126.7, 124.5, 122.7, 122.0, 121.5, 120.3, 120.3, 114.7, 99.9, 25.3, 21.5. HRMS (ESI) calcd for C_30_H_24_N [M + H]^+^: 398.1909, found: 398.1905.

*1,10-Dimethyl-5,6-diphenylindolo[2,1-a]isoquinoline* (**6g**), 64.2 mg, 81% yield, orange solid, m.p. 212.6–213.0 °C. ^1^H NMR (400 MHz, CDCl_3_) δ 7.61 (s, 1H), 7.38 (d, *J* = 7.2 Hz, 1H), 7.36–7.26 (m, 6H), 7.25–7.16 (m, 6H), 7.05 (dd, *J* = 8.0, 1.2 Hz, 1H), 6.68 (dd, *J* = 8.8, 2.0 Hz, 1H), 5.87 (d, *J* = 8.8 Hz, 1H), 3.02 (s, 3H), 2.44 (s, 3H). ^13^C NMR (100 MHz, CDCl_3_) δ 137.7, 136.0, 135.9, 135.6, 135.1, 132.1, 131.9, 131.0, 130.3, 130.2, 130.0, 128.7, 128.0, 126.8, 126.5, 125.1, 124. 5, 122.4, 121.8, 120.0, 114.4, 100.3, 25.4, 21.5. HRMS (ESI) calcd for C_30_H_24_N [M + H]^+^: 398.1909, found: 398.1912.

*5-(4-Ethylphenyl)-1-methyl-6-(p-tolyl)indolo[2,1-a]isoquinoline and 6-(4-ethylphenyl)-1-methyl-5-(p-tolyl)indolo[2,1-a]isoquinoline* (**6h**), 68.6 mg, 82% yield, yellow solid, m.p. 151.6–151.9 °C. ^1^H NMR (400 MHz, CDCl_3_) δ 7.82 (dq, J = 8.0, 1.2 Hz, 1H), 7.54 (s, 1H), 7.37 (d, J = 6.8 Hz, 1H), 7.25–7.12 (m, 6H), 7.07 and 7.05 (a pair of s, 5H), 6.87–6.81 (m, 1H), 6.04 and 5.98 (a pair of dd, J = 8.8, 0.8 Hz, 1H), 3.02 (s, 3H), 2.69 and 2.62 (a pair of q, J = 7.6 Hz, 2H), 2.39 and 2.32 (a pair of s, 3H), 1.27 and 1.22 (a pair of t, J = 7.6 Hz, 3H). ^13^C NMR (100 MHz, CDCl_3_) δ 144.8, 142.5, 138.3, 136.1, 135.6, 135.1, 134.8, 134.6, 133.2, 132.0, 131.8, 130.8, 130.8, 130.1, 129.6, 129.4, 128.7, 128.2, 127.4, 126.5, 125.0, 124.6, 124.5, 122.1, 122.0, 121.4, 120.4, 120.4, 115.0, 100.6, 28.8, 28.6, 25.4, 21.6, 21.4, 15.6, 15.5. HRMS (ESI) calcd for C_32_H_28_N [M + H]^+^: 426.2222, found: 426.2224.

## 4. Conclusions

In conclusion, we have reported on the rhodium-catalyzed oxidative annulation of 2- or 7-phenyl-1*H*-indoles with alkenes or alkynes to assemble valuable 6*H*-isoindolo[2,1-*a*]indoles, pyrrolo[3,2,1-*de*]phenanthridines, or indolo[2,1-*a*]isoquinolines using molecular oxygen as the sole oxidant enable by quaternary ammonium salt. Salient features of present catalytic system comprise (a) the atmospheric pressure of air as the sole oxidant, (b) one catalytic system for three discrete reactions, and (c) mechanistic insights. Mechanistic studies provided support for fast intramolecular aza-Michael reaction and aerobic reoxidation of Rh(I) to Rh(III) by adding quaternary ammonium salt. Additional mechanistic/computational studies will be needed to fully elucidate the unique influence of quaternary ammonium salt on the catalytic cycle, and are in progress in our laboratory.

## Figures and Tables

**Table 1:**
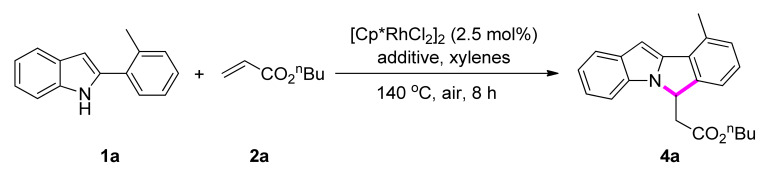
Optimization of the reaction conditions *^a^*.

Entry	Additive	Yield (%) (3a) *^b^*
1 *^c^*	-	0
2	*n*-Bu_4_NOAc	93 (91 *^d^*)
3	Me_4_NOAc	88
4	*n*-Bu_4_NPF_6_	trace
5	*n*-Bu_4_NBF_4_	0
6	*n*-Bu_4_NHSO_4_	0
7	*n*-Bu_4_NCl	trace
8	*n*-Bu_4_NI	0
9	NH_4_Cl	0
10	NH_4_PF_6_	trace
11	Et_4_NBr	0
12 *^e^*	*n*-Bu_4_NOAc	trace

*^a^*: Reaction condition: **1a** (0.2 mmol), **2a** (0.4 mmol), [Cp*RhCl_2_]_2_ (2.5 mol%), and additive (1 equiv.) in xylenes (4 mL) at 140 °C under air for 8 h. *^b^*: Determined by ^1^H NMR yield using CH_2_Br_2_ as an internal standard. *^c^*: Xylenes, DMF, THF, EtOAc, or 1,4-dioxane. *^d^*: Isolated yield. *^e^*: In the absence of [Cp*RhCl_2_]_2_ or under N_2_ atmosphere.

## Data Availability

The data presented in this study are available in Supplementary Materials.

## Supplementary Materials

The following are available online. Figure S1: Copies of the ^1^H NMR, ^13^C NMR charts for compounds.

## Sample Availability

Samples of the compounds are available from the authors.
