# Supplementary material for: Rhodium-Catalyzed Oxidative Annulation of 2- or 7-Arylindoles with Alkenes/Alkynes Using Molecular Oxygen as the Sole Oxidant Enabled by Quaternary Ammonium Salt
Source: Molecules. 2021 Sep 2;26(17):5329. doi: 10.3390/molecules26175329 (PMC8433977; doi:10.3390/molecules26175329)
Supplement: Supplementary file 1 [file molecules-26-05329-s001.zip › molecules-1355692-SI.pdf]

Supplementary Materials

Rhodium-Catalyzed Oxidative Annulation of 2- or 7-Arylindoles with Alkenes/Alkynes Using Molecular Oxygen as the Sole Oxidant Enabled by Quaternary Ammonium Salt

Weihui Zhuang [1], Jiaqi Zhang [1], Yanping Zheng [1] and Qiufeng Huang [1,2,*]

[1] Fujian Key Laboratory of Polymer Materials, College of Chemistry & Materials Science, Fujian Normal University, Fuzhou 350007, China; zwh180652@163.com (W.Z.); jiaqi13206681102@163.com (J.Z.); zhengyanping1108@163.com (Y.Z.)
[2] Fujian Provincial Key Laboratory of Advanced Materials Oriented Chemical Engineering, Fuzhou 35007, China
* Correspondence: qiufenghuang@fjnu.edu.cn; Tel.: +86-135-9908-1672

Contents:

1. Copies of ^{1}H and ^{13}C NMR charts for compounds

(S2 to S44)

molecules

MDPI

Figure S1. Copies of ^{1}H and ^{13}C NMR charts for compounds

(1) The ^{1}H NMR spectrum for **3a**

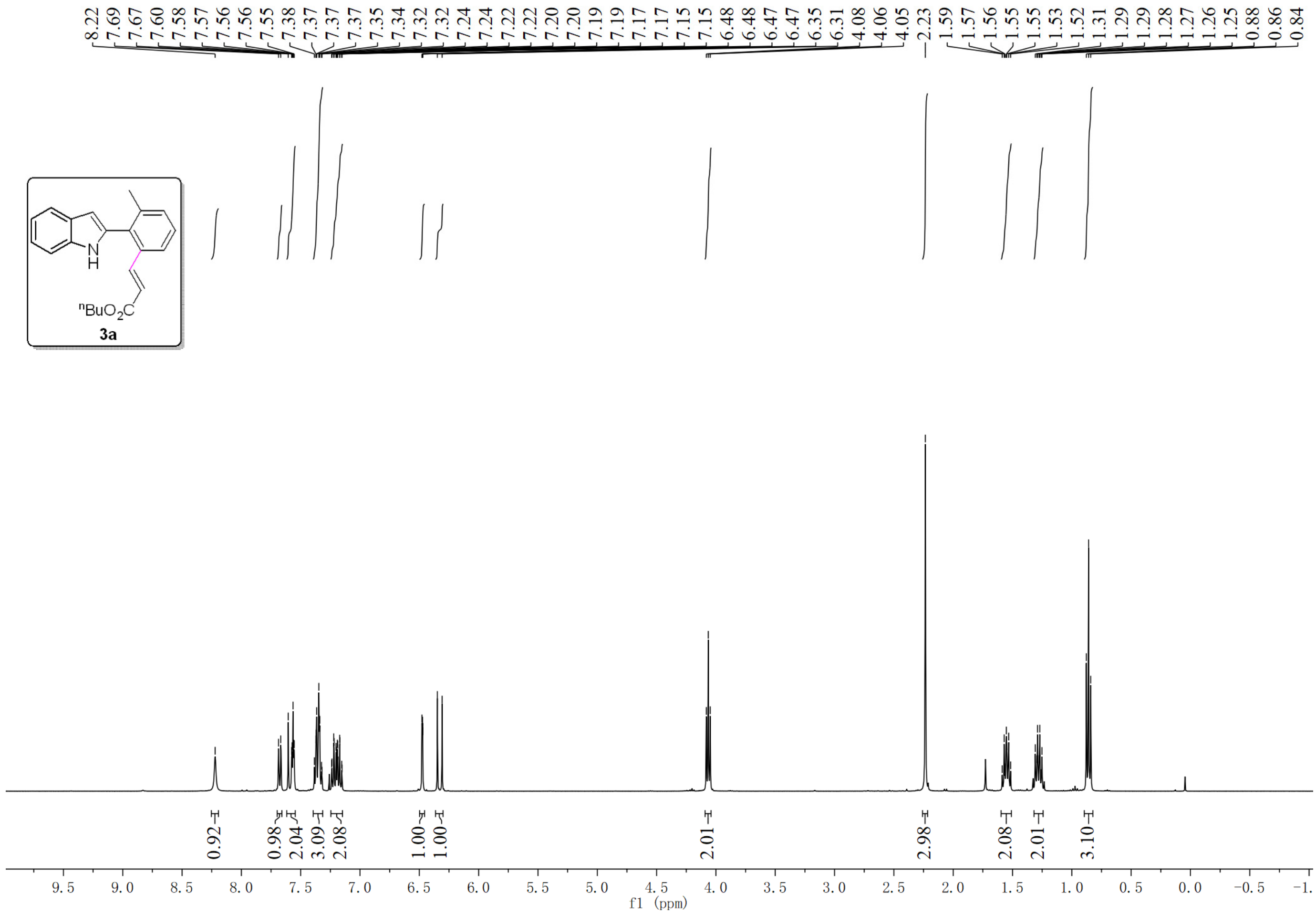

(2) The ^{1}H NMR spectrum for **3b**

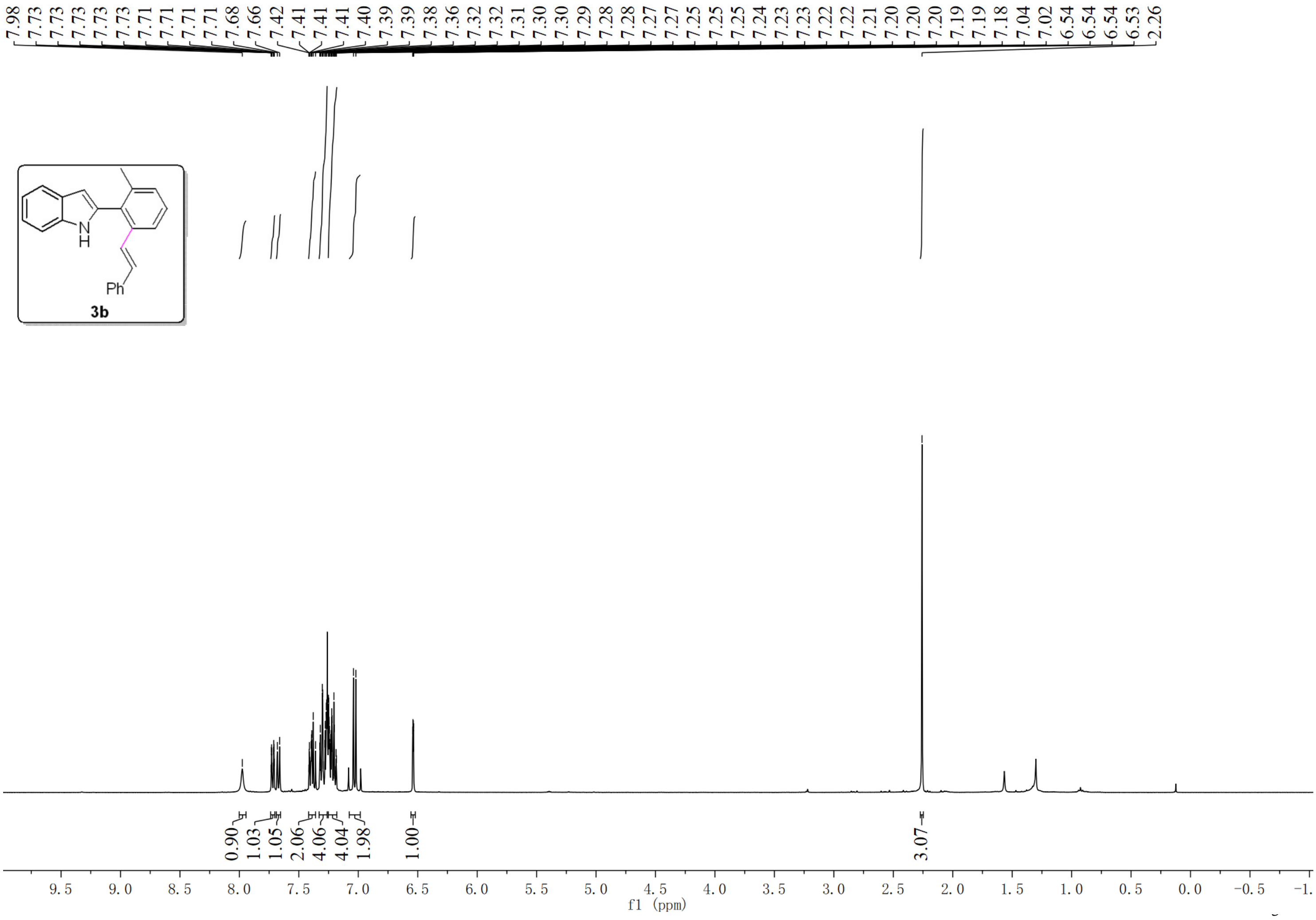

(3) The ^{1}H NMR and ^{13}C NMR spectrum for **4a**

6

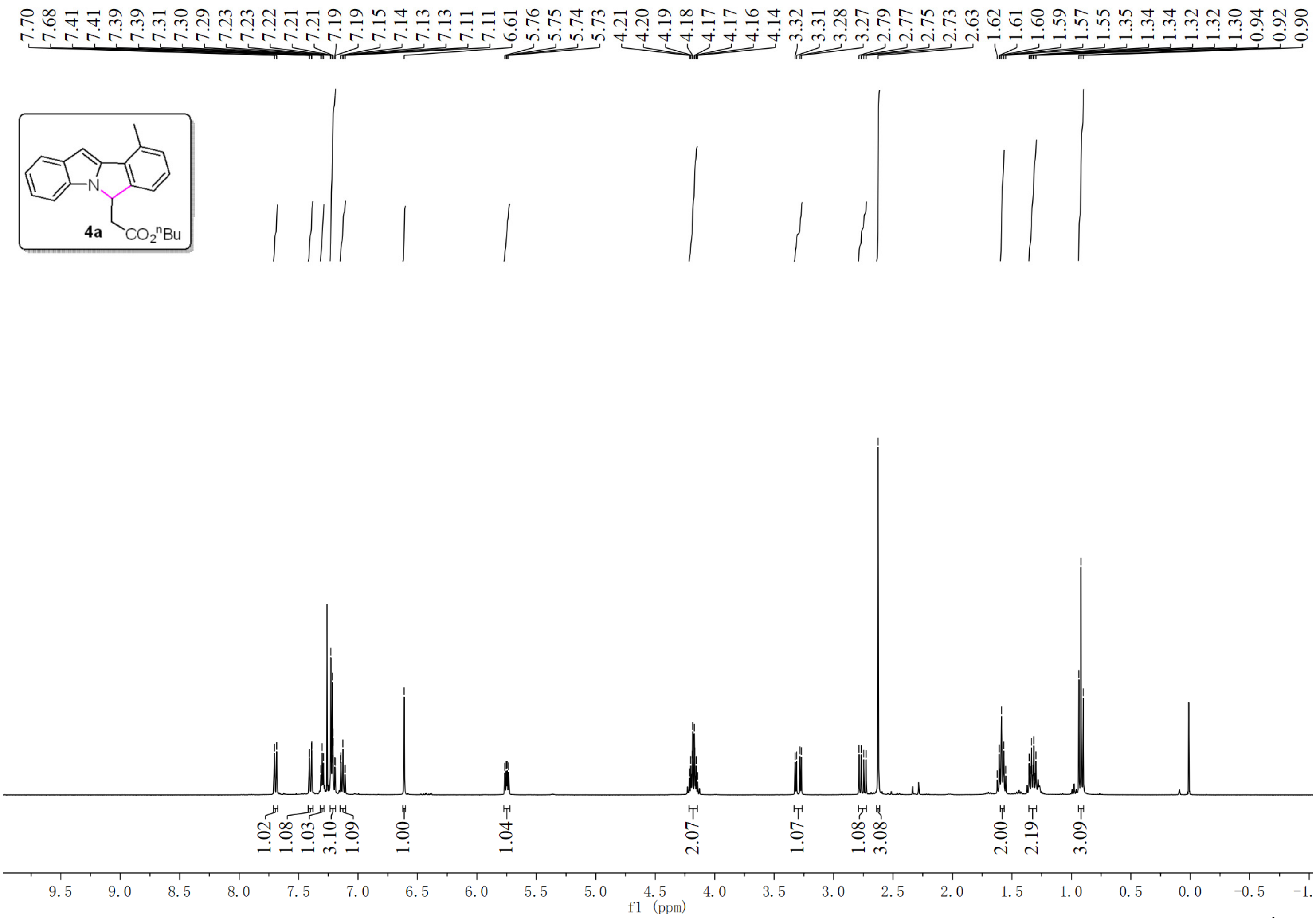
7.70
7.68
7.41
7.41
7.39
7.39
7.31
7.30
7.29
7.23
7.23
7.22
7.21
7.21
7.19
7.19
7.15
7.14
7.13
7.13
7.11
7.11
6.61
5.76
5.75
5.74
5.73
4.21
4.20
4.19
4.18
4.17
4.17
4.16
4.14
3.32
3.31
3.28
3.27
2.79
2.77
2.75
2.73
2.63
1.62
1.61
1.60
1.59
1.57
1.55
1.35
1.34
1.34
1.32
1.32
1.30
0.94
0.92
0.90
4a
CO2nBu
N
1.02
1.08
1.03
3.10
1.09
1.00
1.04
2.07
1.07
1.08
3.08
2.00
2.19
3.09
9.5
9.0
8.5
8.0
7.5
7.0
6.5
6.0
5.5
5.0
4.5
4.0
3.5
3.0
2.5
2.0
1.5
1.0
0.5
0.0
-0.5
-1.
f1 (ppm)

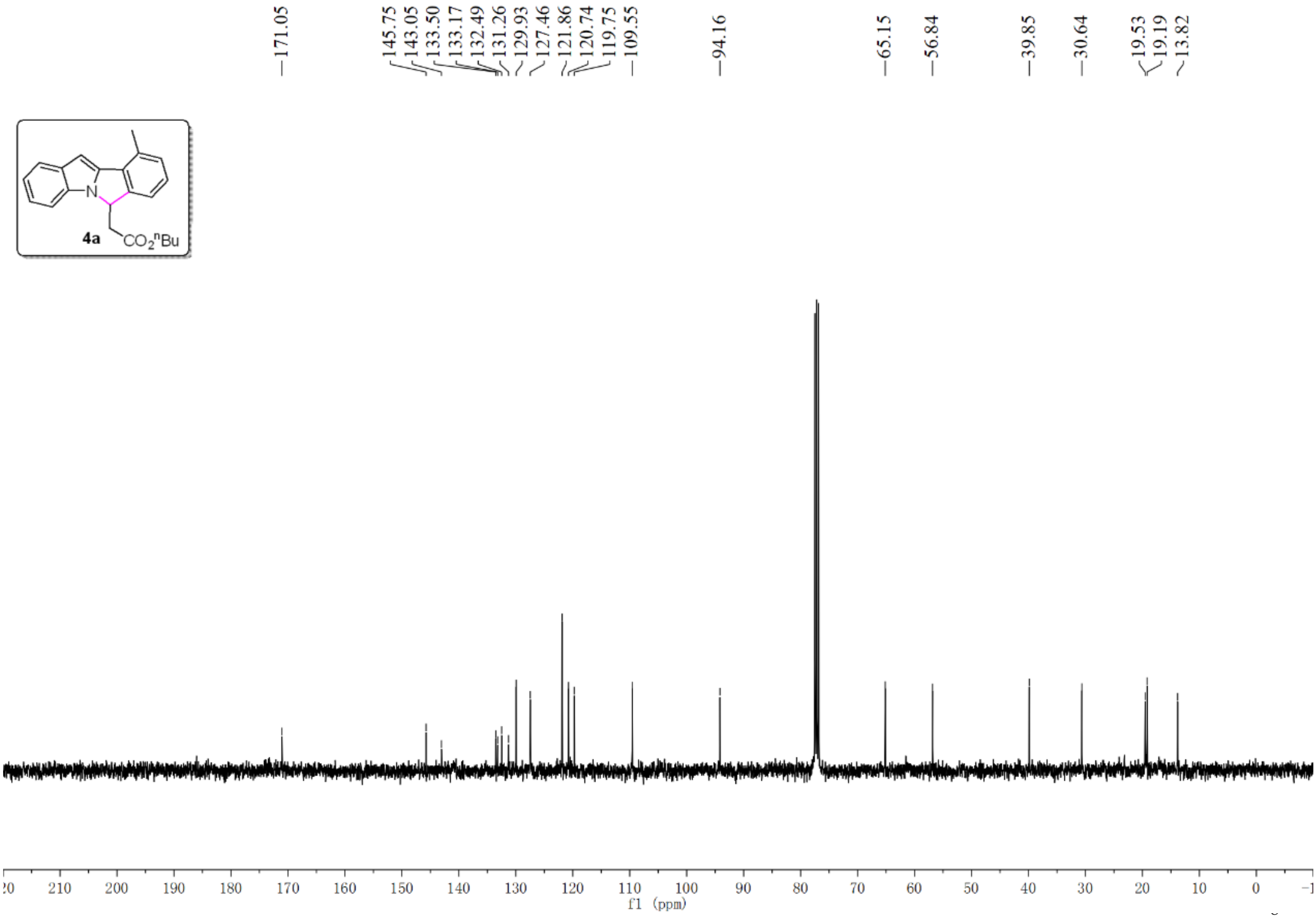

(4) The ^{1}H NMR spectrum for **4b**

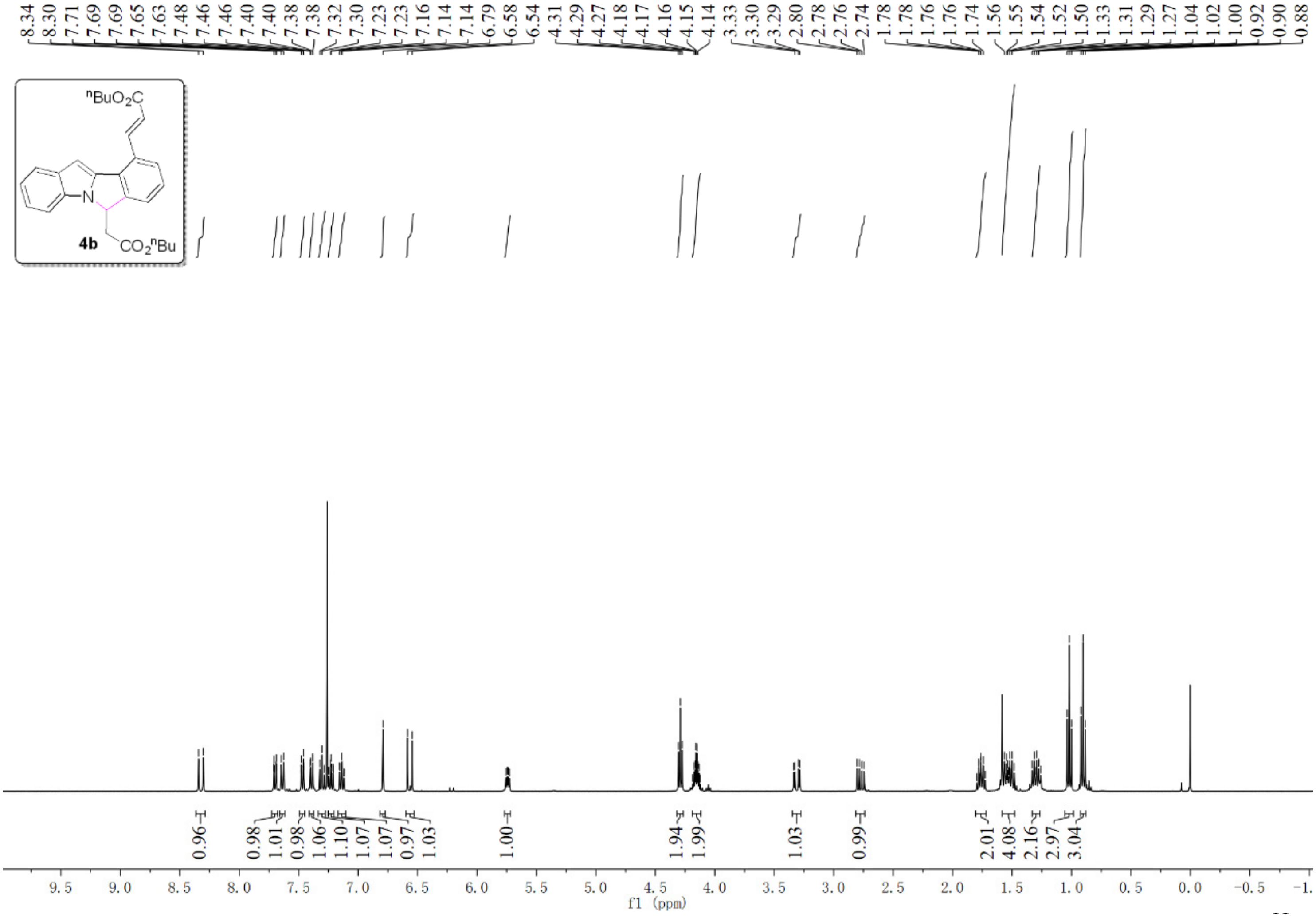

(5) The ^{1}H NMR and ^{13}C NMR spectrum for **4c**

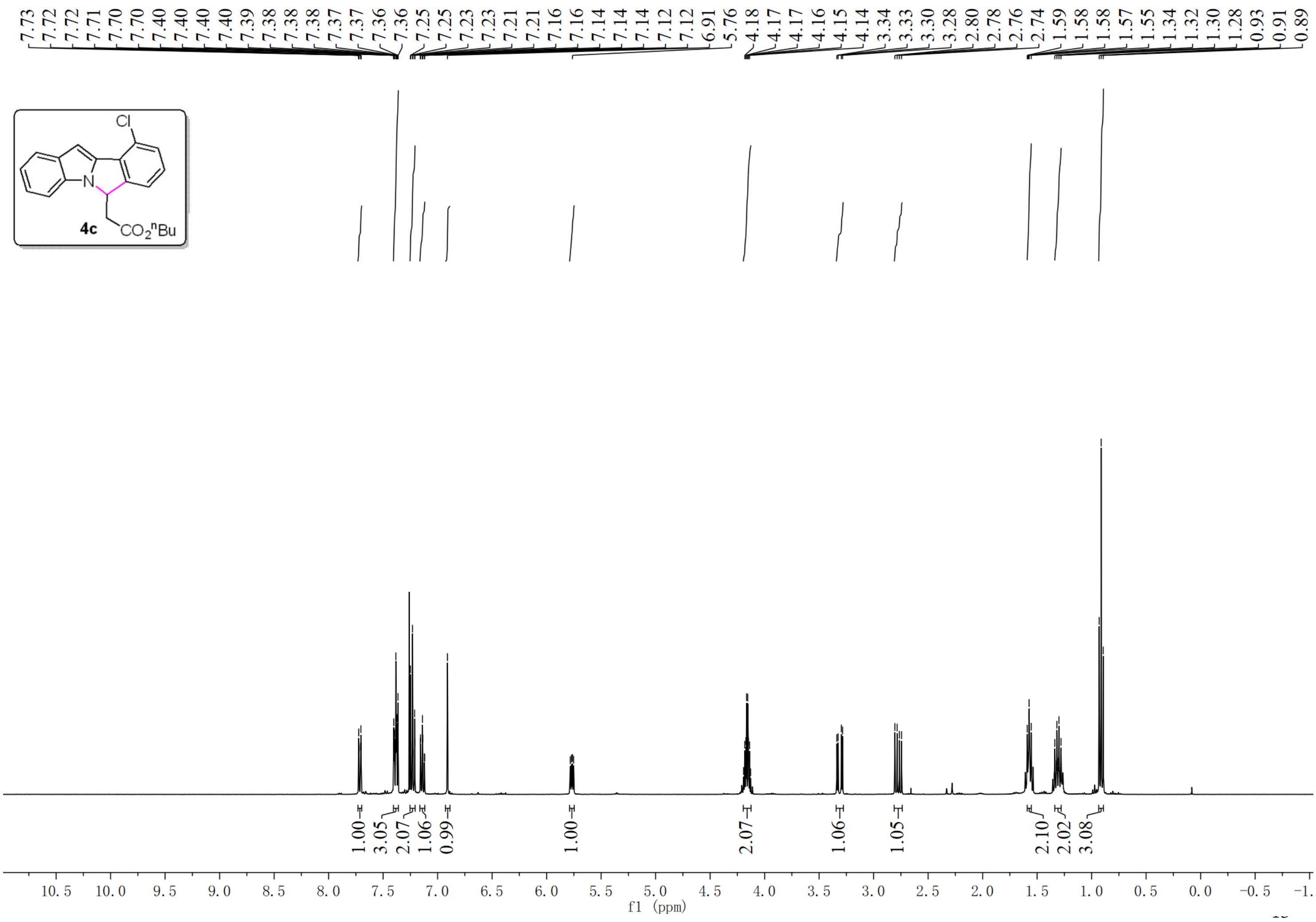
7.73
7.72
7.72
7.71
7.70
7.70
7.40
7.40
7.40
7.40
7.39
7.38
7.38
7.38
7.37
7.37
7.36
7.36
7.25
7.25
7.23
7.23
7.21
7.21
7.16
7.16
7.14
7.14
7.14
7.12
7.12
6.91
5.76
4.18
4.17
4.17
4.16
4.15
4.14
3.34
3.33
3.30
3.28
2.80
2.78
2.76
2.74
1.59
1.58
1.58
1.57
1.55
1.34
1.32
1.30
1.28
0.93
0.91
0.89
Cl
N
4c
CO2nBu
1.00
3.05
2.07
1.06
0.99
1.00
2.07
1.06
1.05
2.10
2.02
3.08
10.5
10.0
9.5
9.0
8.5
8.0
7.5
7.0
6.5
6.0
5.5
5.0
4.5
4.0
3.5
3.0
2.5
2.0
1.5
1.0
0.5
0.0
-0.5
f1 (ppm)

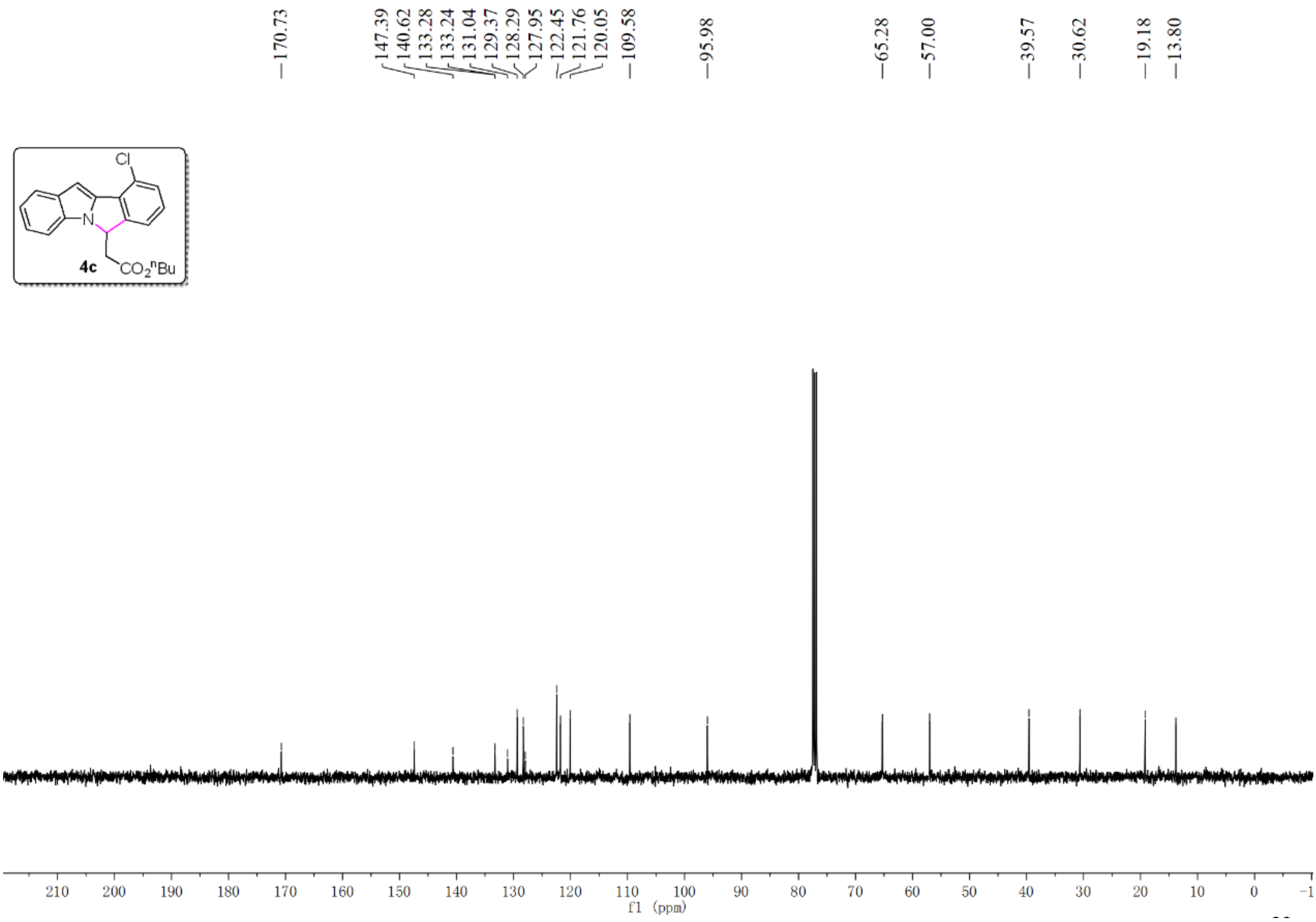

(6) The ^{1}H NMR and ^{13}C NMR spectrum for **4d**

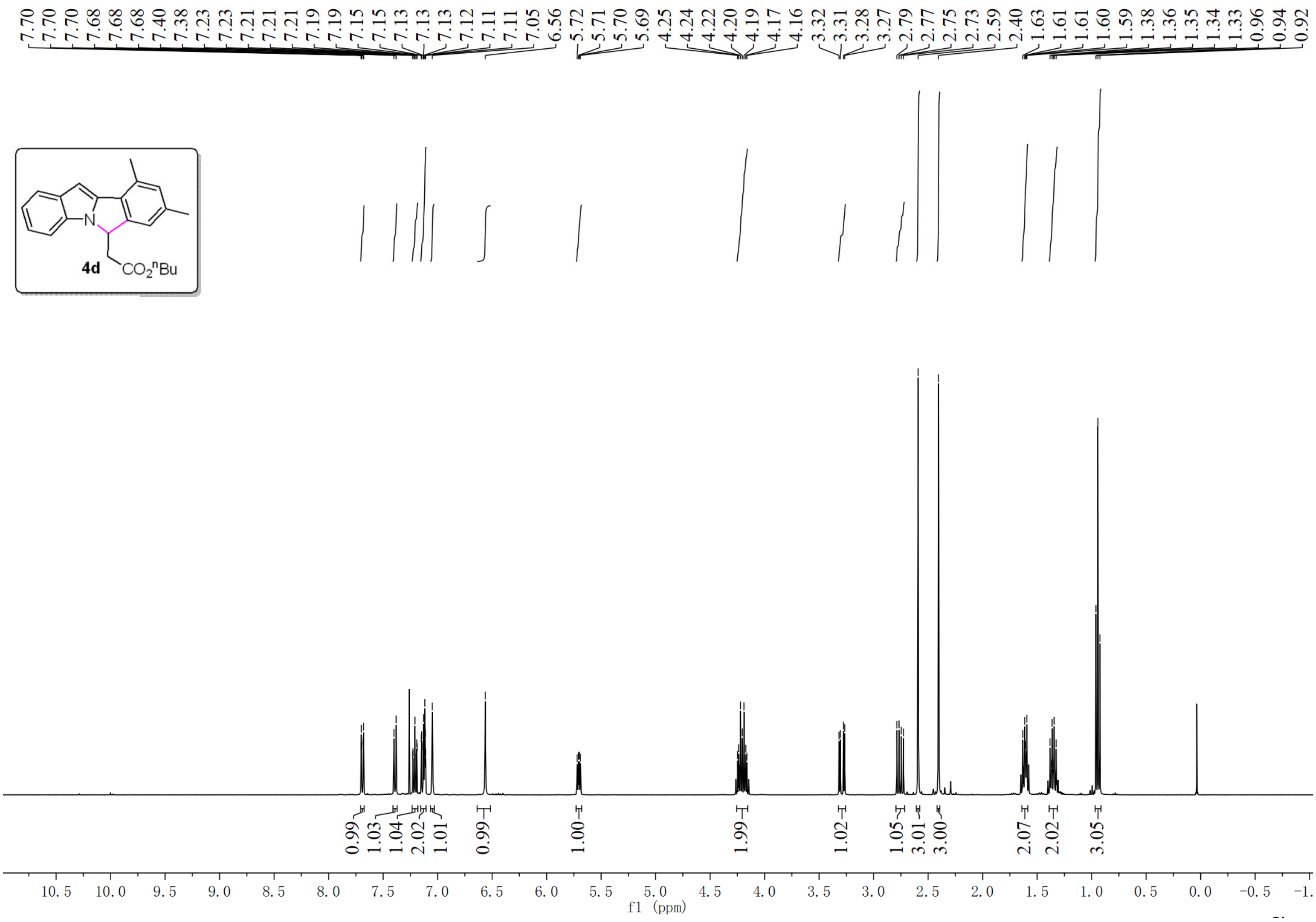
4d
$CO_2{}^nBu$
f1 (ppm)

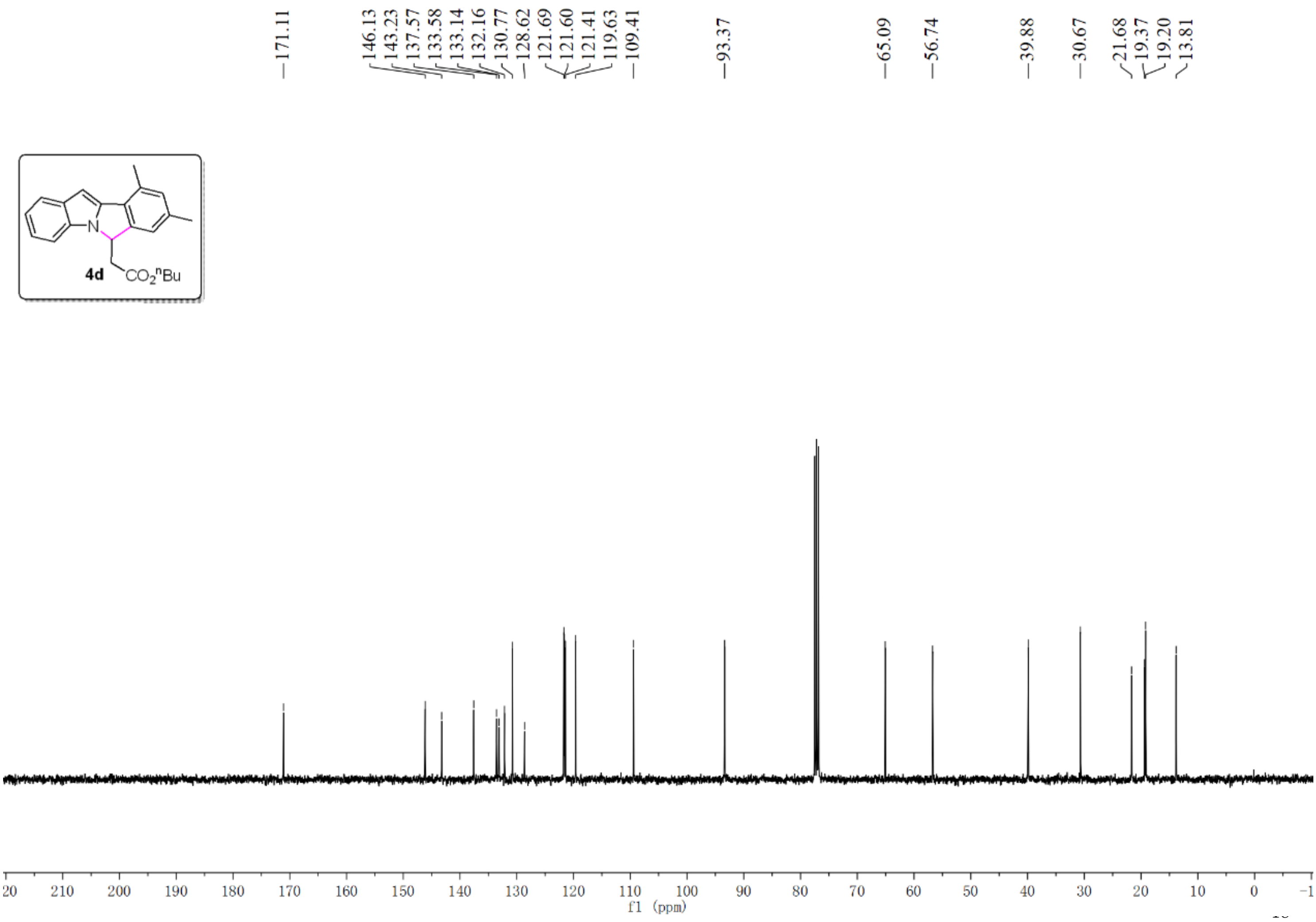

(7) The ^{1}H NMR and ^{13}C NMR spectrum for **4e**

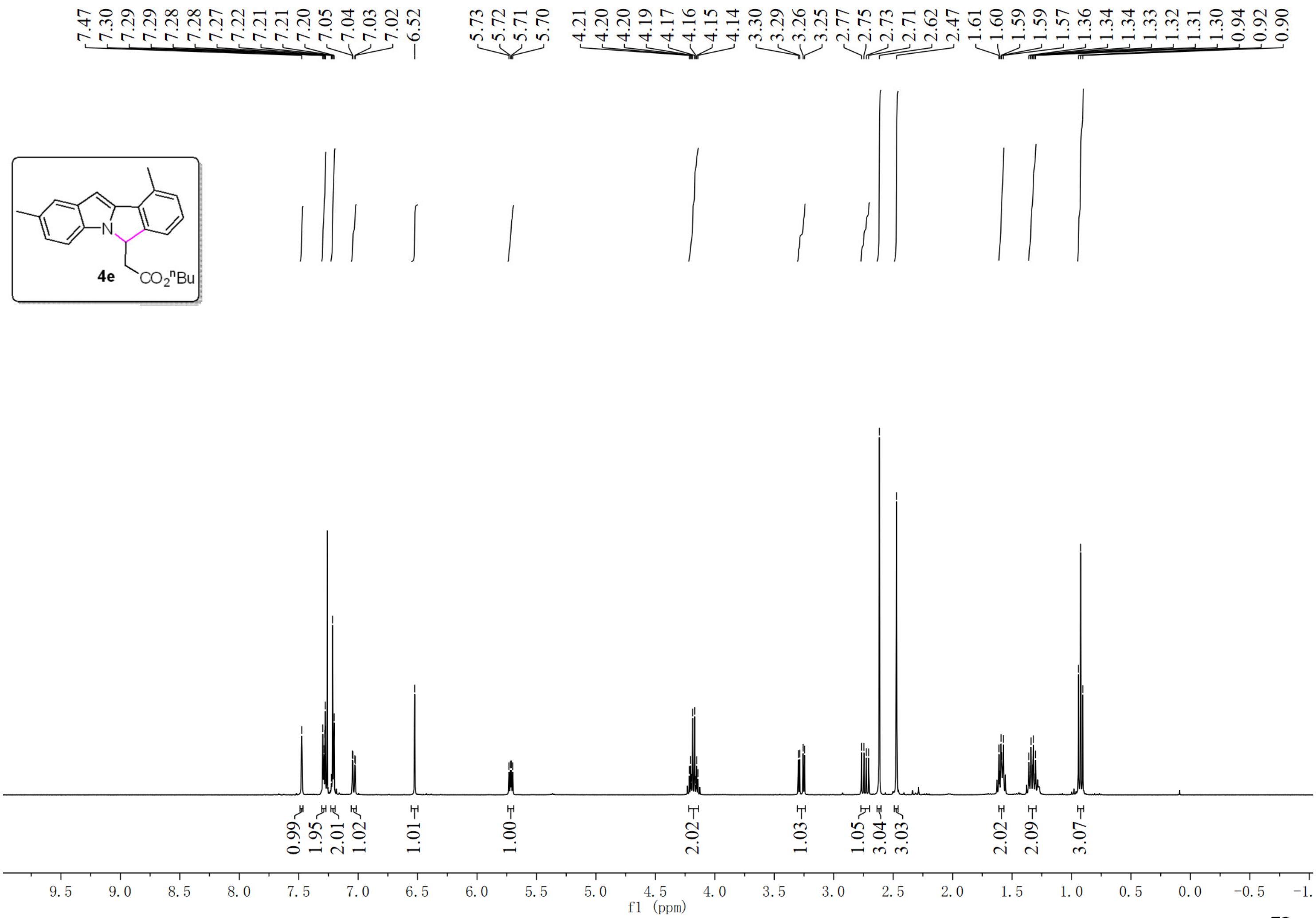
7.47 7.30 7.29 7.29 7.28 7.28 7.27 7.22 7.21 7.21 7.20 7.05 7.04 7.03 7.02 6.52
5.73 5.72 5.71 5.70
4.21 4.20 4.20 4.19 4.17 4.16 4.15 4.14
3.30 3.29 3.26 3.25
2.77 2.75 2.73 2.71 2.62 2.47
1.61 1.60 1.59 1.59 1.57 1.36 1.34 1.34 1.33 1.32 1.31 1.30
0.94 0.92 0.90
N
4e
CO2nBu
0.99 1.95 2.01 1.02 1.01 1.00 2.02 1.03 1.05 3.04 3.03 2.02 2.09 3.07
9.5 9.0 8.5 8.0 7.5 7.0 6.5 6.0 5.5 5.0 4.5 4.0 3.5 3.0 2.5 2.0 1.5 1.0 0.5 0.0 −0.5 −1.
f1 (ppm)

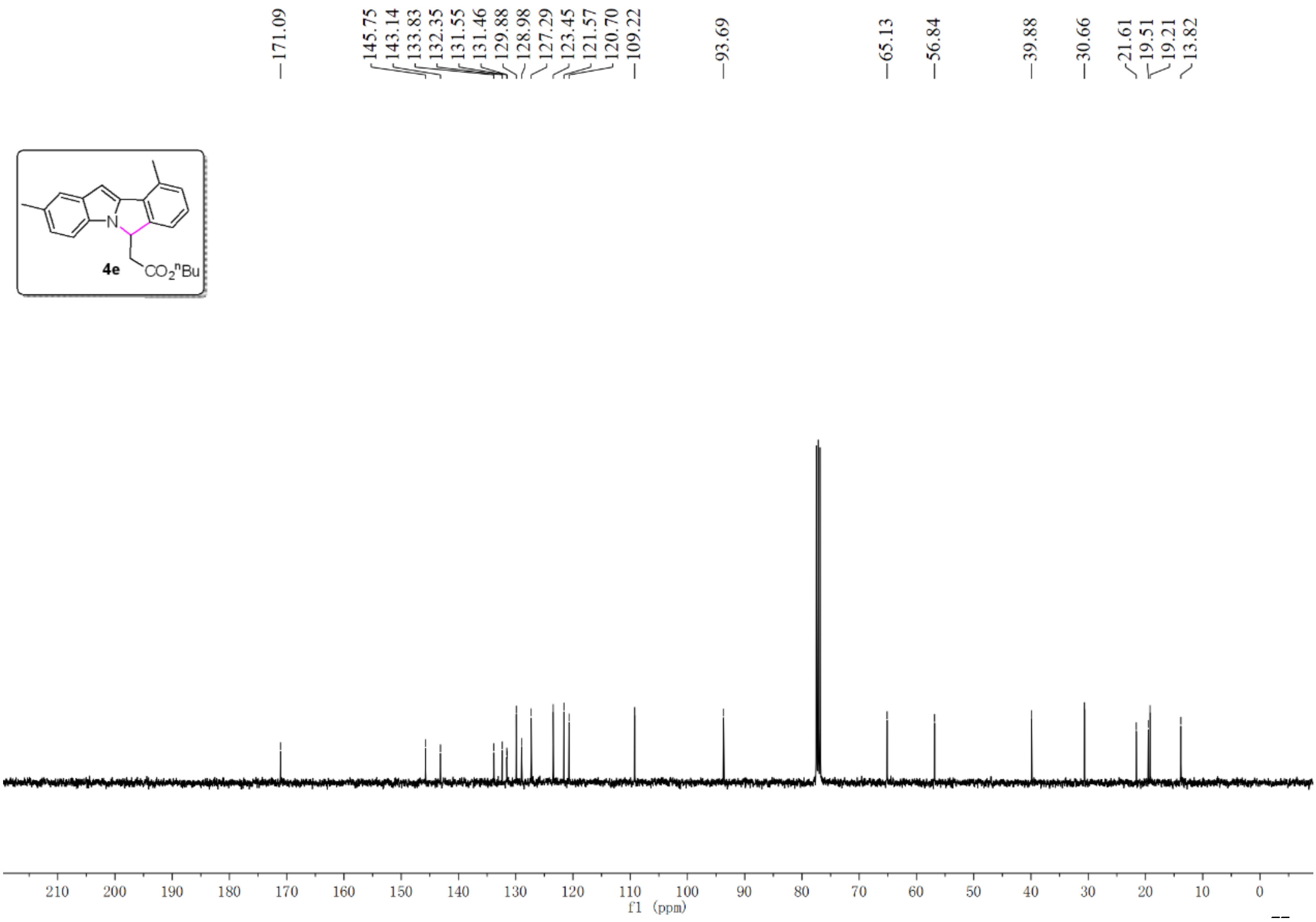

(8) The ^{1}H NMR and ^{13}C NMR spectrum for **4f**

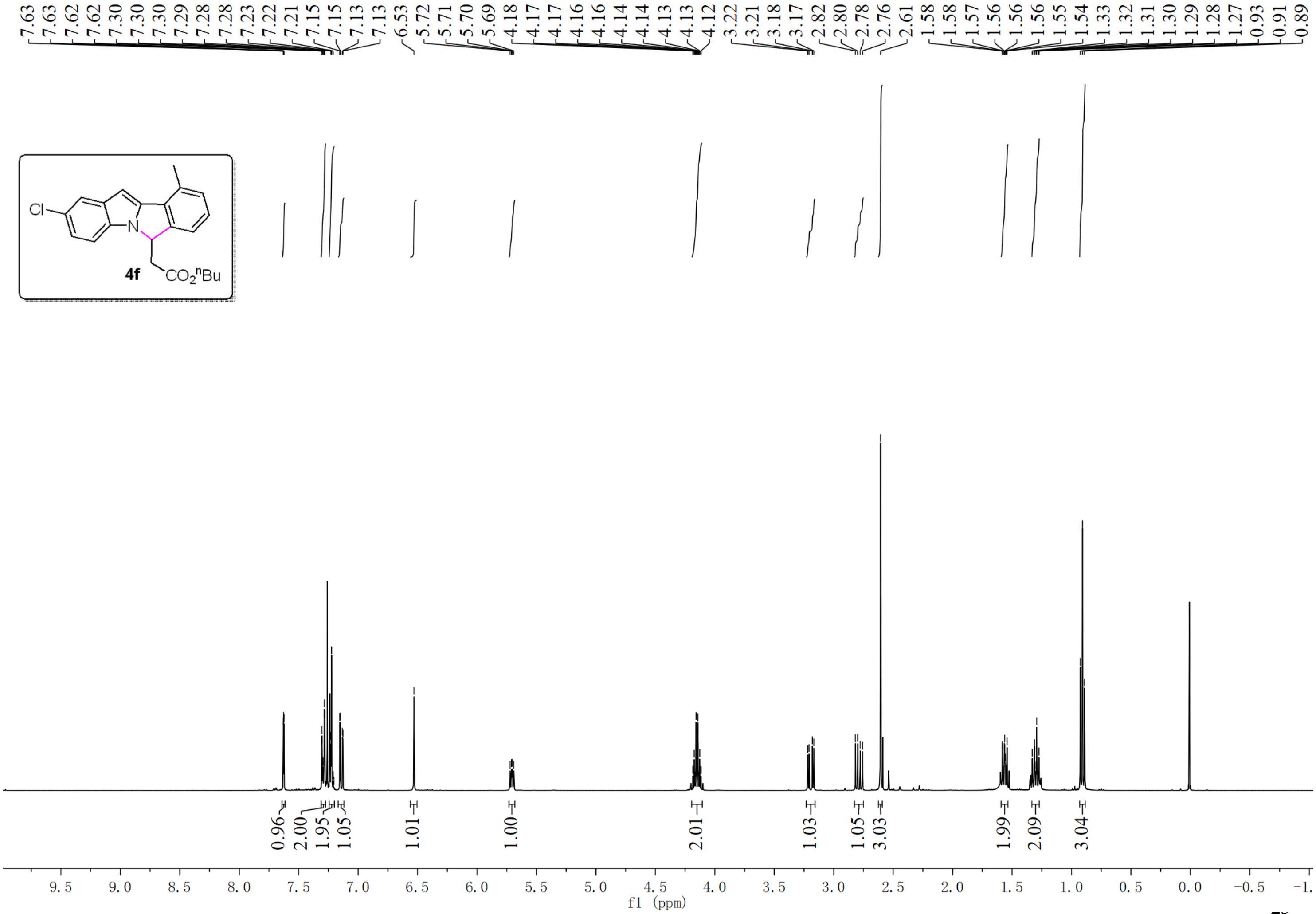
7.63
7.63
7.62
7.62
7.30
7.30
7.29
7.28
7.28
7.23
7.22
7.21
7.15
7.15
7.13
7.13
6.53
5.72
5.71
5.70
5.69
4.18
4.17
4.17
4.16
4.16
4.14
4.14
4.13
4.13
4.12
3.22
3.21
3.18
3.17
2.82
2.80
2.78
2.76
2.61
1.58
1.58
1.57
1.56
1.56
1.56
1.55
1.54
1.33
1.32
1.31
1.30
1.29
1.28
1.27
0.93
0.91
0.89
Cl
N
4f
CO2nBu
0.96
2.00
1.95
1.05
1.01
1.00
2.01
1.03
1.05
3.03
1.99
2.09
3.04
f1 (ppm)

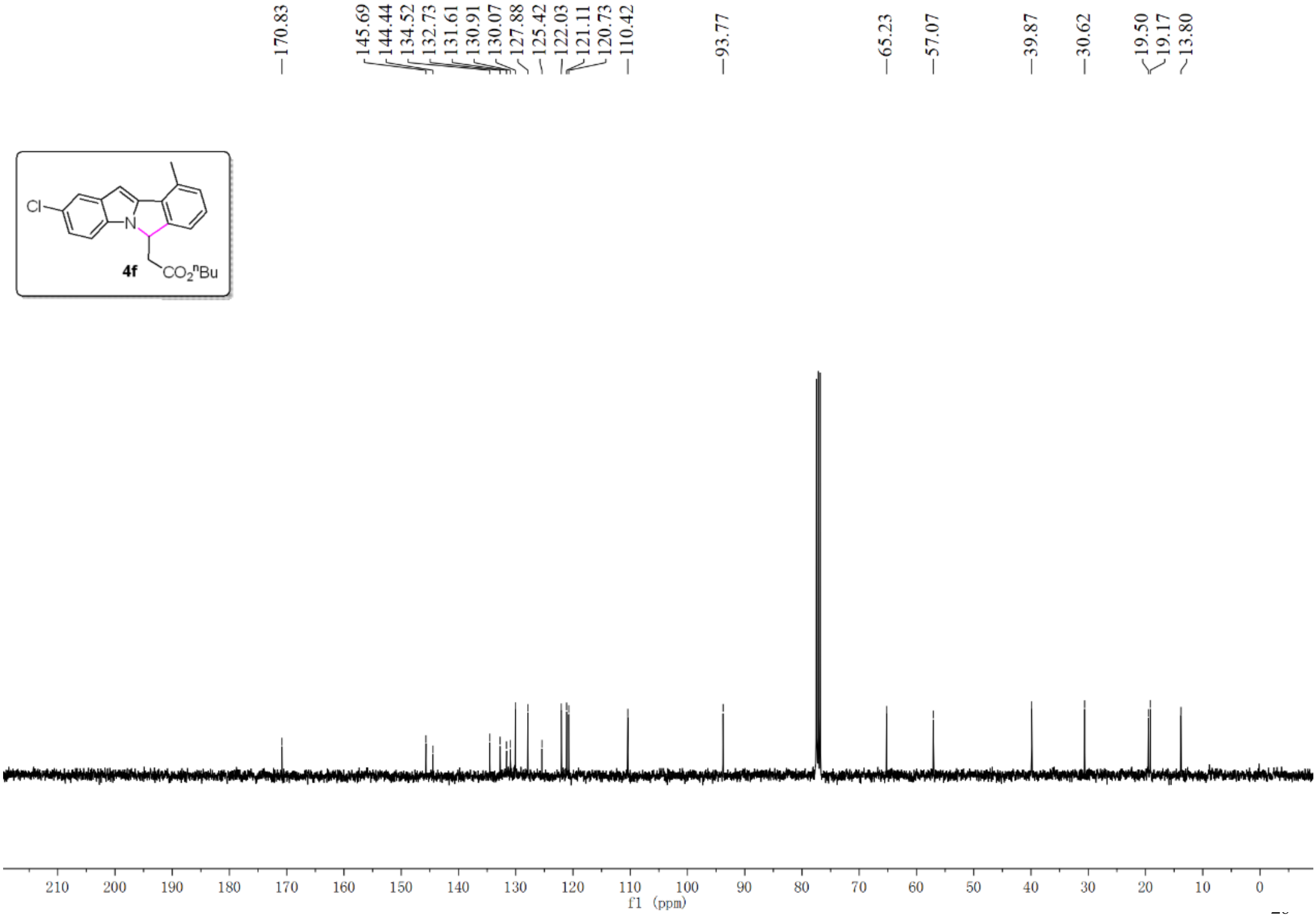

(9) The ^{1}H NMR and ^{13}C NMR spectrum for **4g**

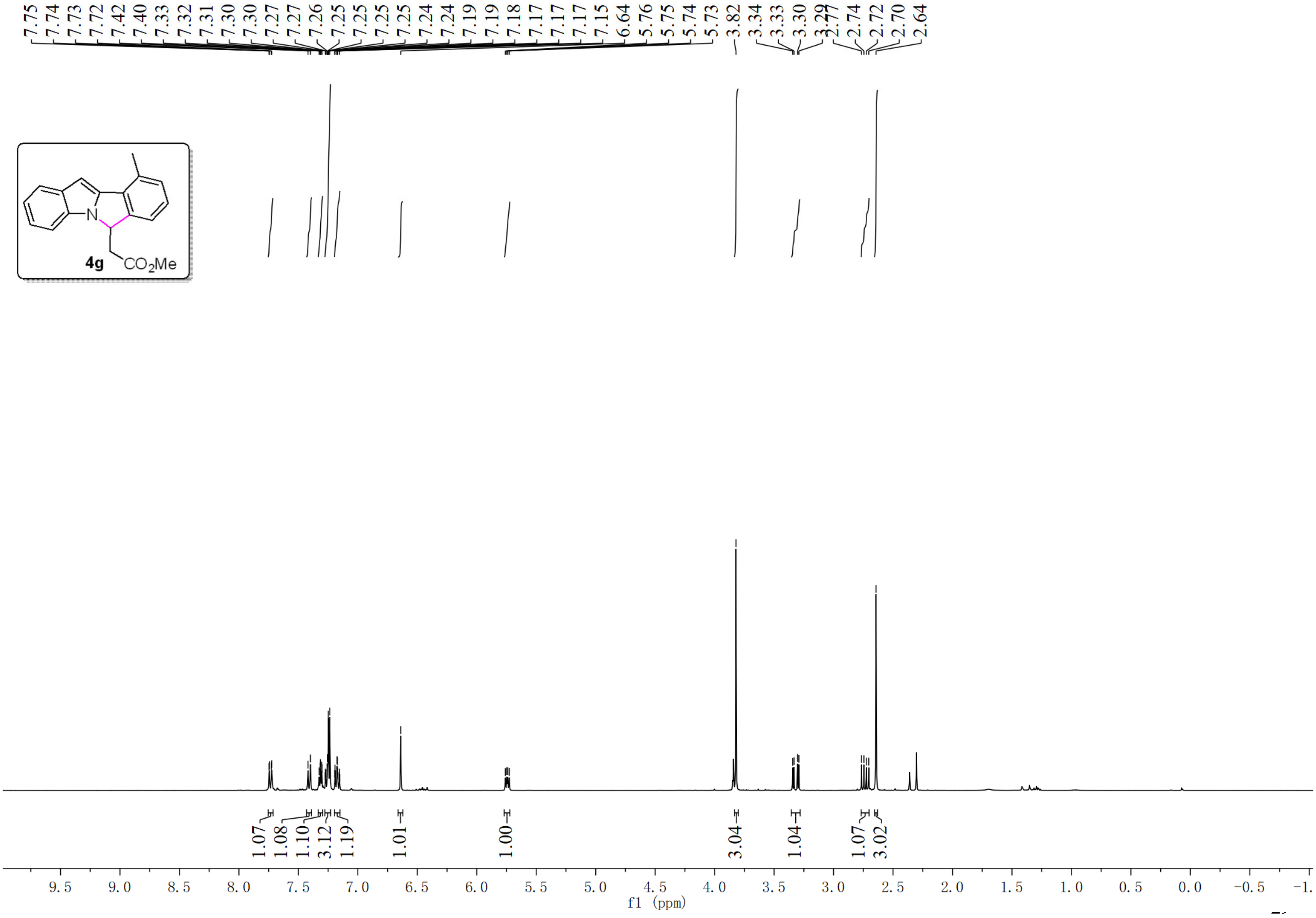
4g
CO2Me
N
7.75
7.74
7.73
7.72
7.42
7.40
7.33
7.32
7.31
7.30
7.30
7.27
7.27
7.26
7.25
7.25
7.25
7.25
7.24
7.24
7.19
7.19
7.18
7.17
7.17
7.17
7.15
6.64
5.76
5.75
5.74
5.73
3.82
3.34
3.33
3.30
3.29
2.77
2.74
2.72
2.70
2.64
1.07
1.08
1.10
3.12
1.19
1.01
1.00
3.04
1.04
1.07
3.02
9.5
9.0
8.5
8.0
7.5
7.0
6.5
6.0
5.5
5.0
4.5
4.0
3.5
3.0
2.5
2.0
1.5
1.0
0.5
0.0
-0.5
f1 (ppm)

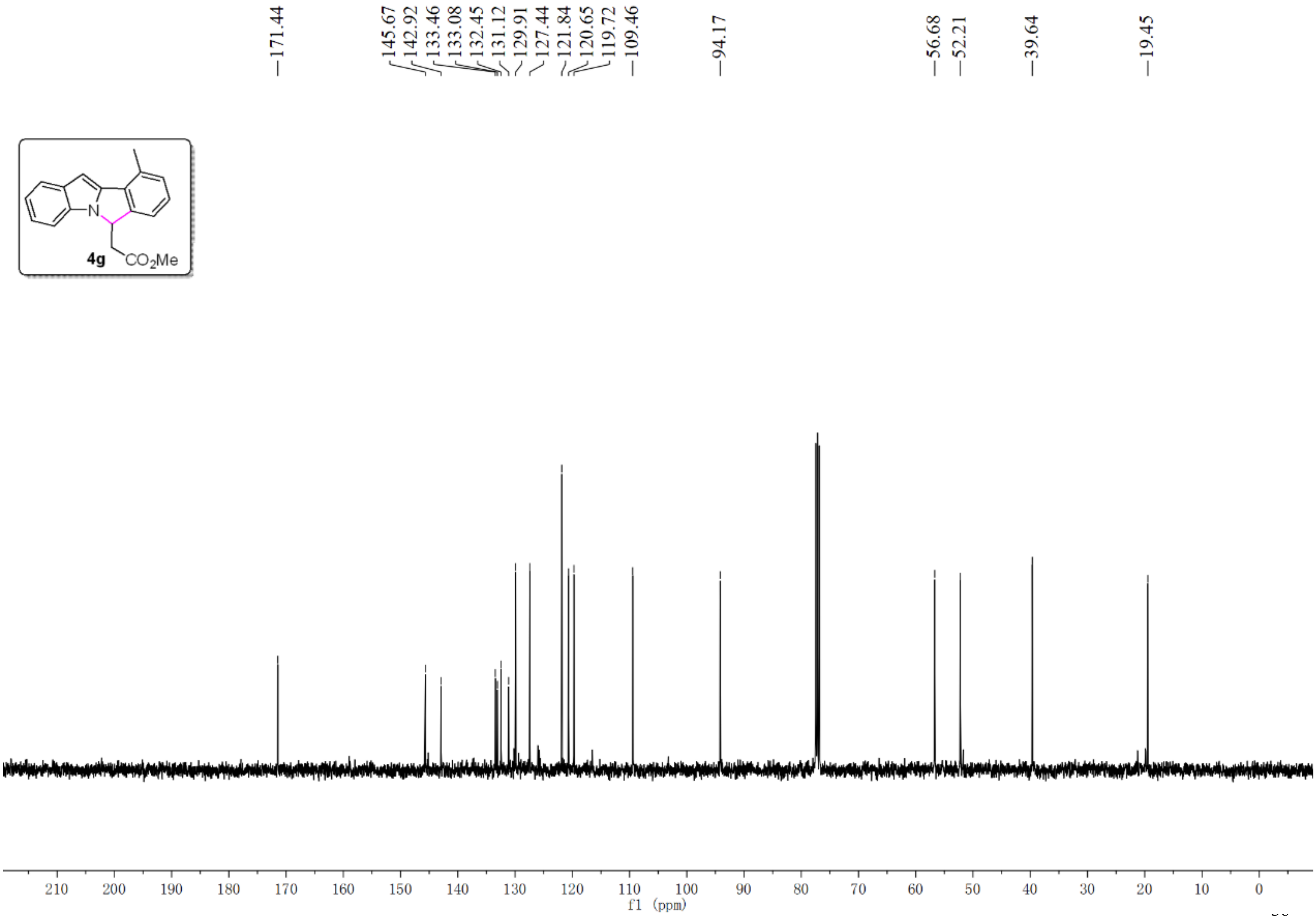

(10) The ^{1}H NMR and ^{13}C NMR spectrum for **4h**

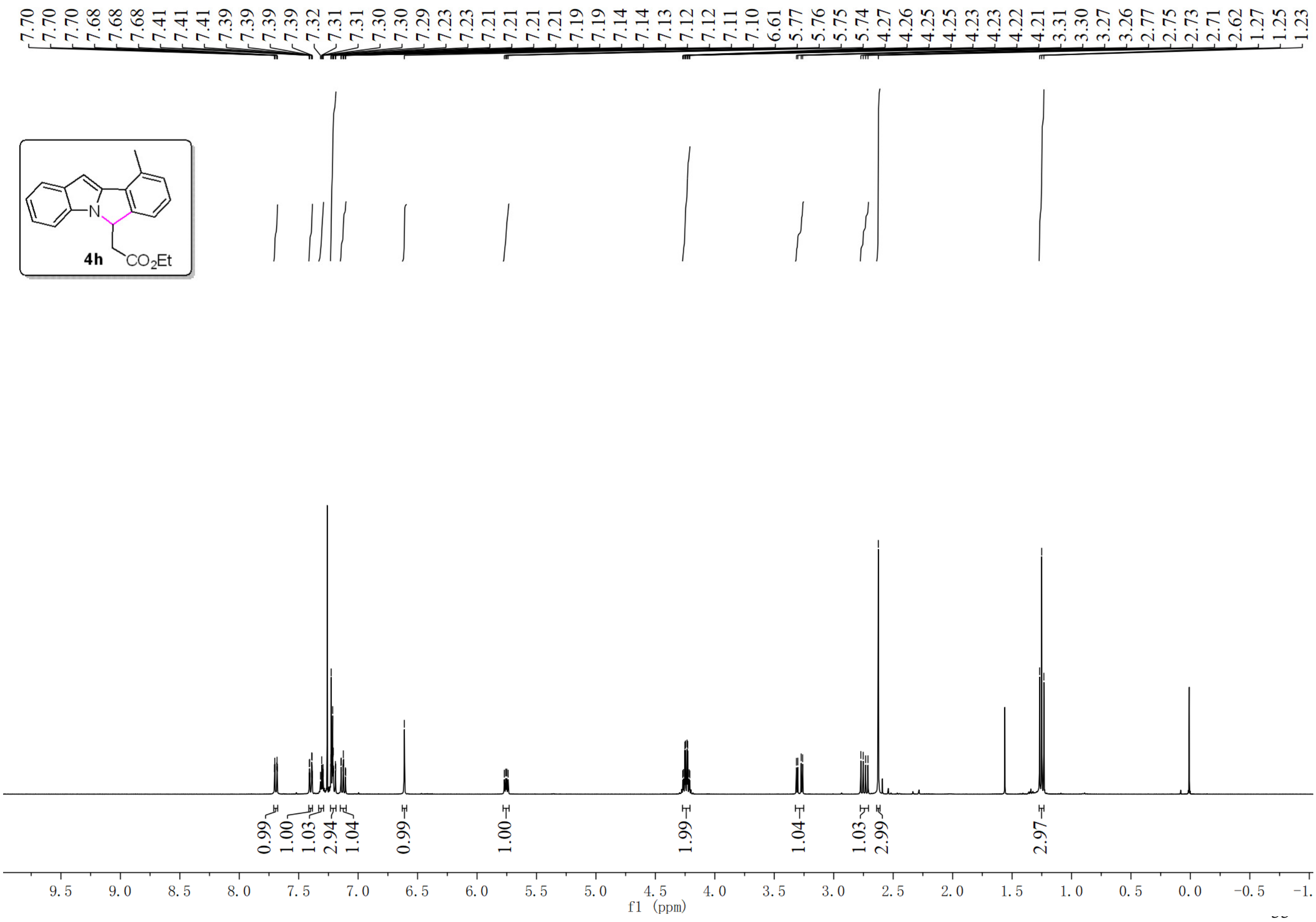
7.70 7.70 7.70 7.68 7.68 7.68 7.41 7.41 7.41 7.39 7.39 7.39 7.39 7.32 7.31 7.31 7.30 7.30 7.29 7.23 7.23 7.21 7.21 7.21 7.21 7.19 7.19 7.14 7.14 7.13 7.12 7.12 7.11 7.10 6.61 5.77 5.76 5.75 5.74 4.27 4.26 4.25 4.25 4.23 4.23 4.22 4.21 3.31 3.30 3.27 3.26 2.77 2.75 2.73 2.71 2.62 1.27 1.25 1.23
4h
CO_2Et
N
0.99 1.00 1.03 2.94 1.04 0.99 1.00 1.99 1.04 1.03 2.99 2.97
9.5 9.0 8.5 8.0 7.5 7.0 6.5 6.0 5.5 5.0 4.5 4.0 3.5 3.0 2.5 2.0 1.5 1.0 0.5 0.0 −0.5 −1.
f1 (ppm)

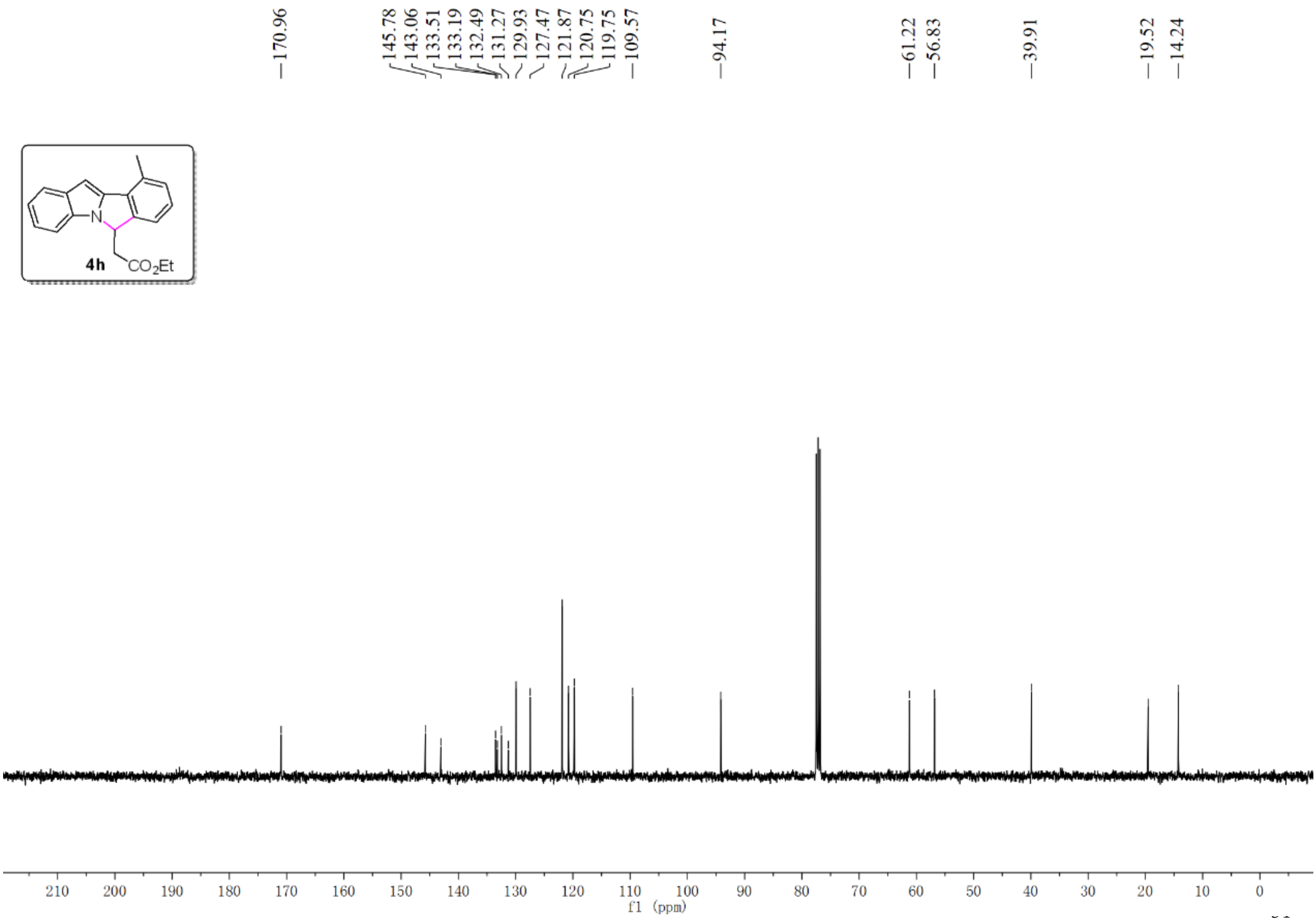

(11) The ^{1}H NMR and ^{13}C NMR spectrum for **4i**

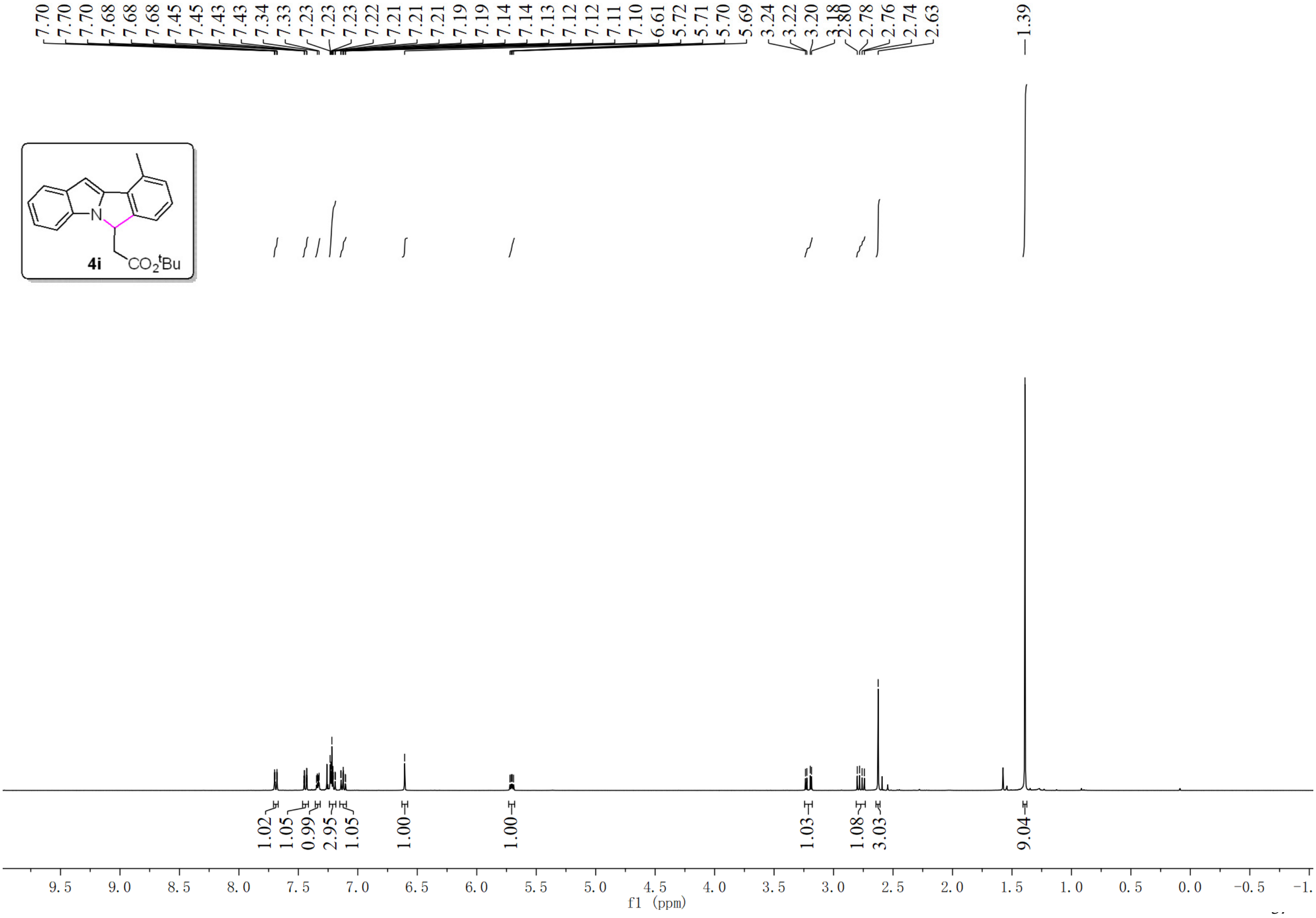
N
4i
CO2tBu
7.70
7.70
7.70
7.68
7.68
7.68
7.45
7.45
7.43
7.43
7.34
7.33
7.23
7.23
7.23
7.22
7.21
7.21
7.21
7.19
7.19
7.14
7.14
7.13
7.12
7.12
7.11
7.10
6.61
5.72
5.71
5.70
5.69
3.24
3.22
3.20
3.18
2.80
2.78
2.76
2.74
2.63
1.39
1.02
1.05
0.99
2.95
1.05
1.00
1.00
1.03
1.08
3.03
9.04
9.5
9.0
8.5
8.0
7.5
7.0
6.5
6.0
5.5
5.0
4.5
4.0
3.5
3.0
2.5
2.0
1.5
1.0
0.5
0.0
−0.5
f1 (ppm)

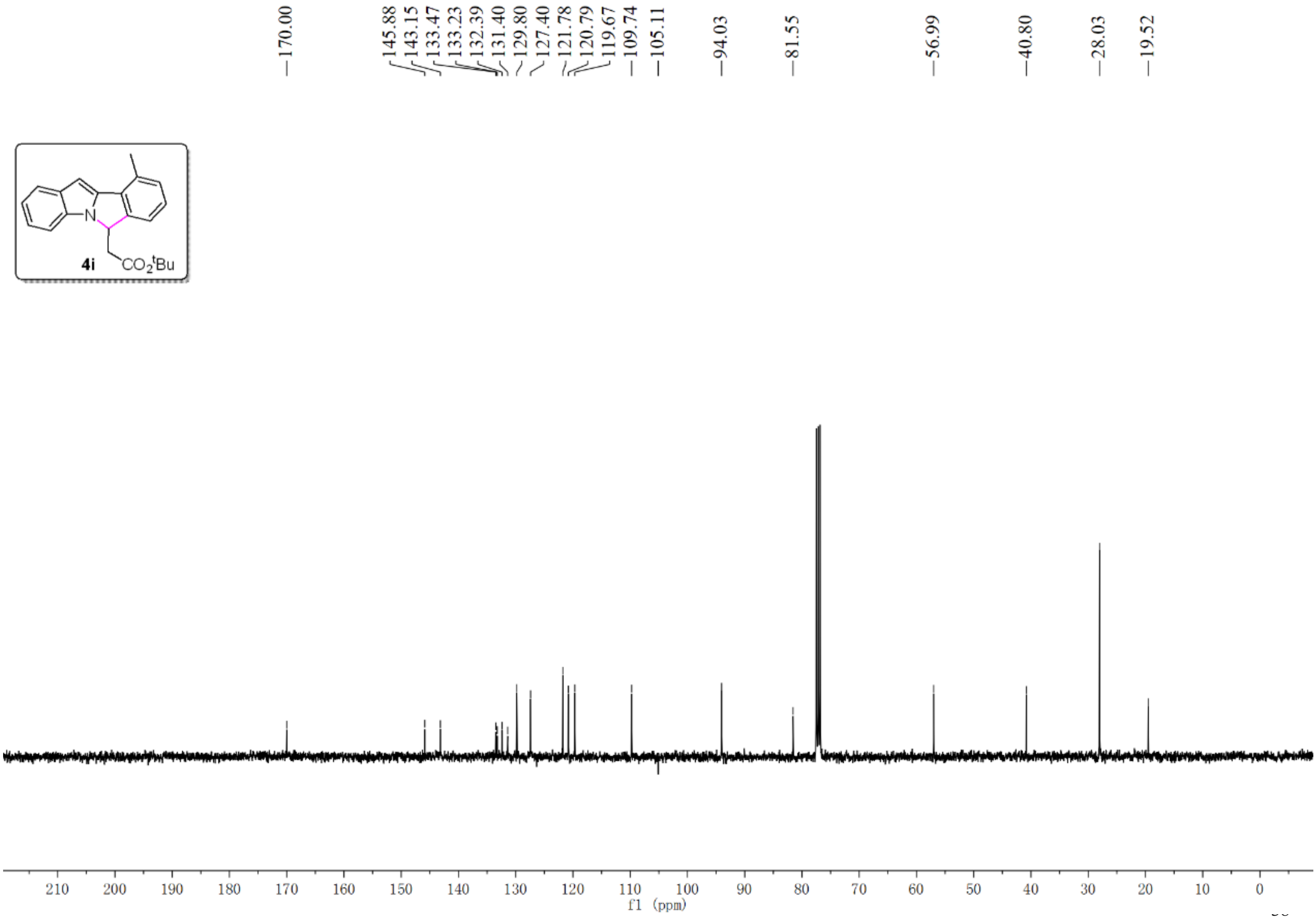

(12) The ^{1}H NMR spectrum for **4j**

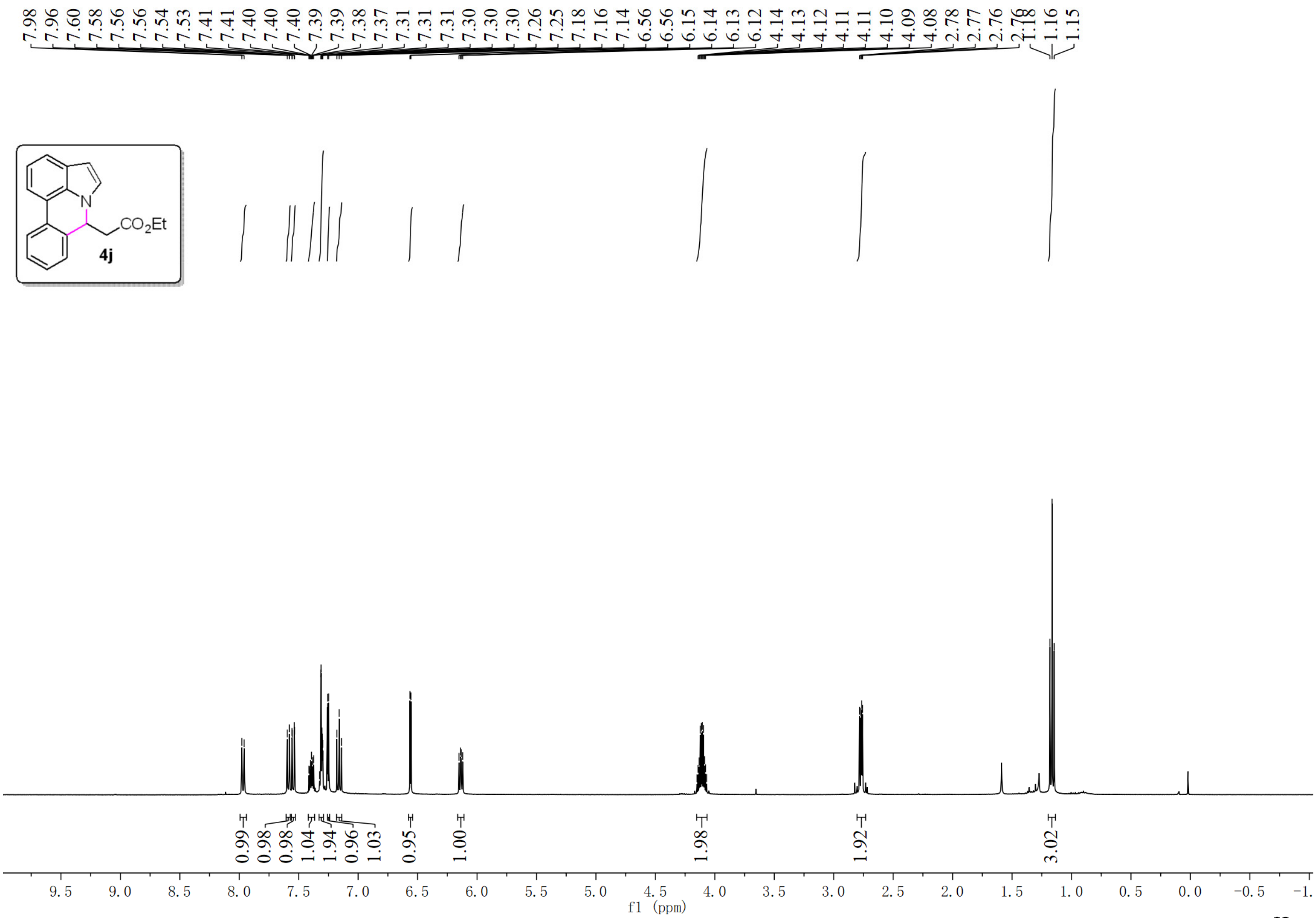

(13) The ^{1}H NMR spectrum for **4k**

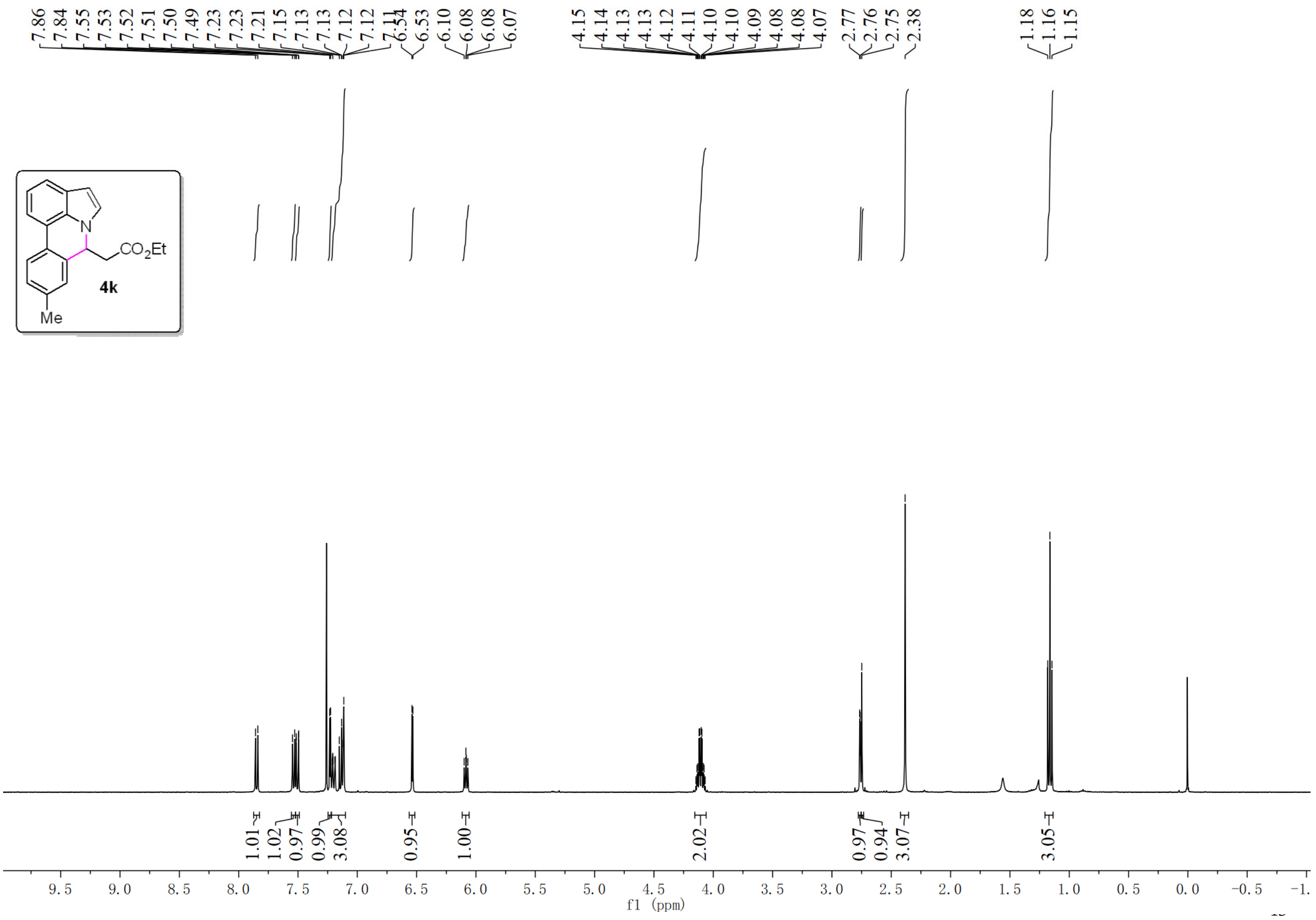

(14) The ^{1}H NMR spectrum for **4l**

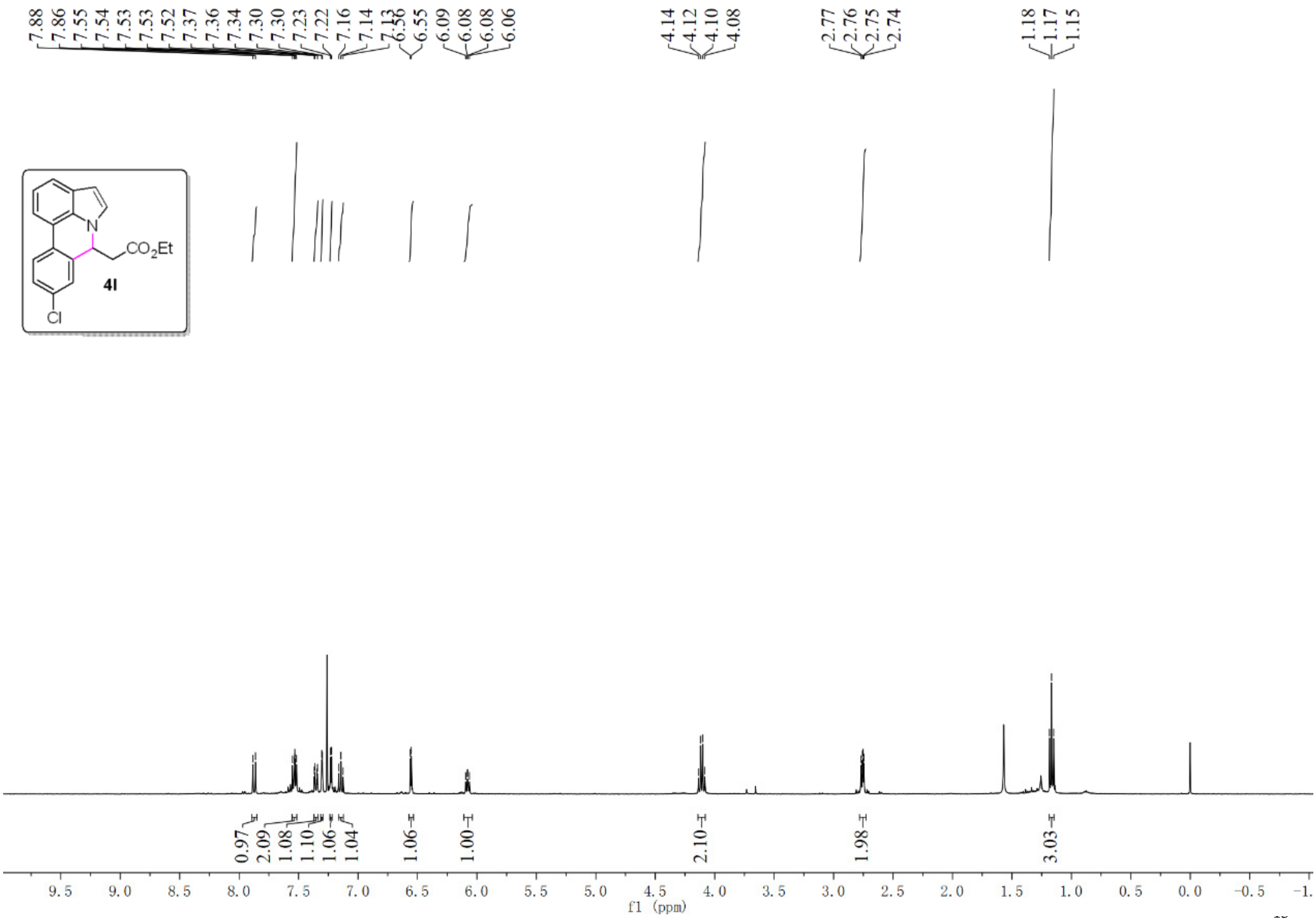

(15) The ^{1}H NMR and ^{13}C NMR spectrum for **4m**

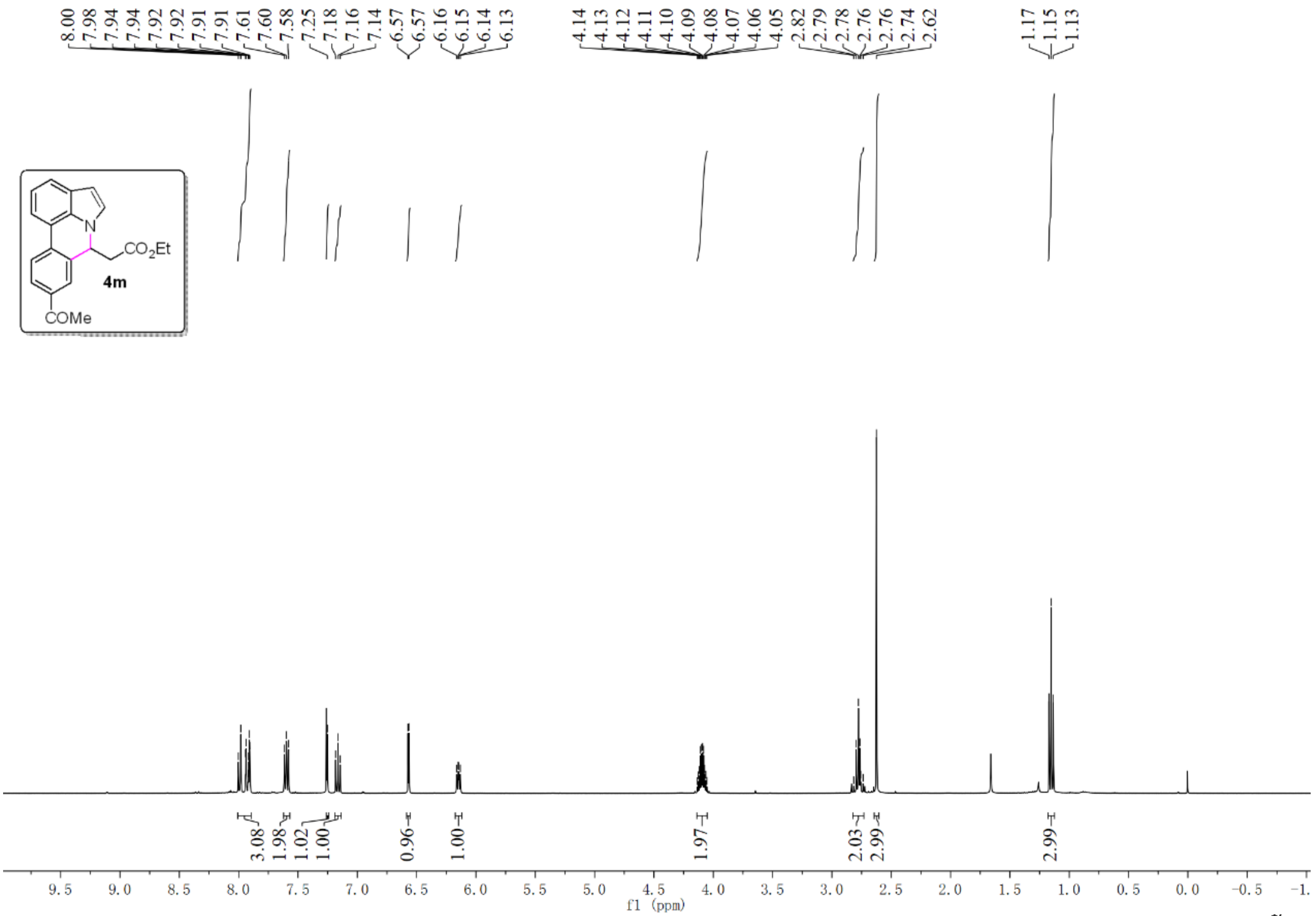
CO2Et
4m
COMe
N
8.00
7.98
7.94
7.94
7.92
7.92
7.91
7.91
7.61
7.60
7.58
7.25
7.18
7.16
7.14
6.57
6.57
6.16
6.15
6.14
6.13
4.14
4.13
4.12
4.11
4.10
4.09
4.08
4.07
4.06
4.05
2.82
2.79
2.78
2.76
2.76
2.74
2.62
1.17
1.15
1.13
3.08
1.98
1.02
1.00
0.96
1.00
1.97
2.03
2.99
2.99
f1 (ppm)

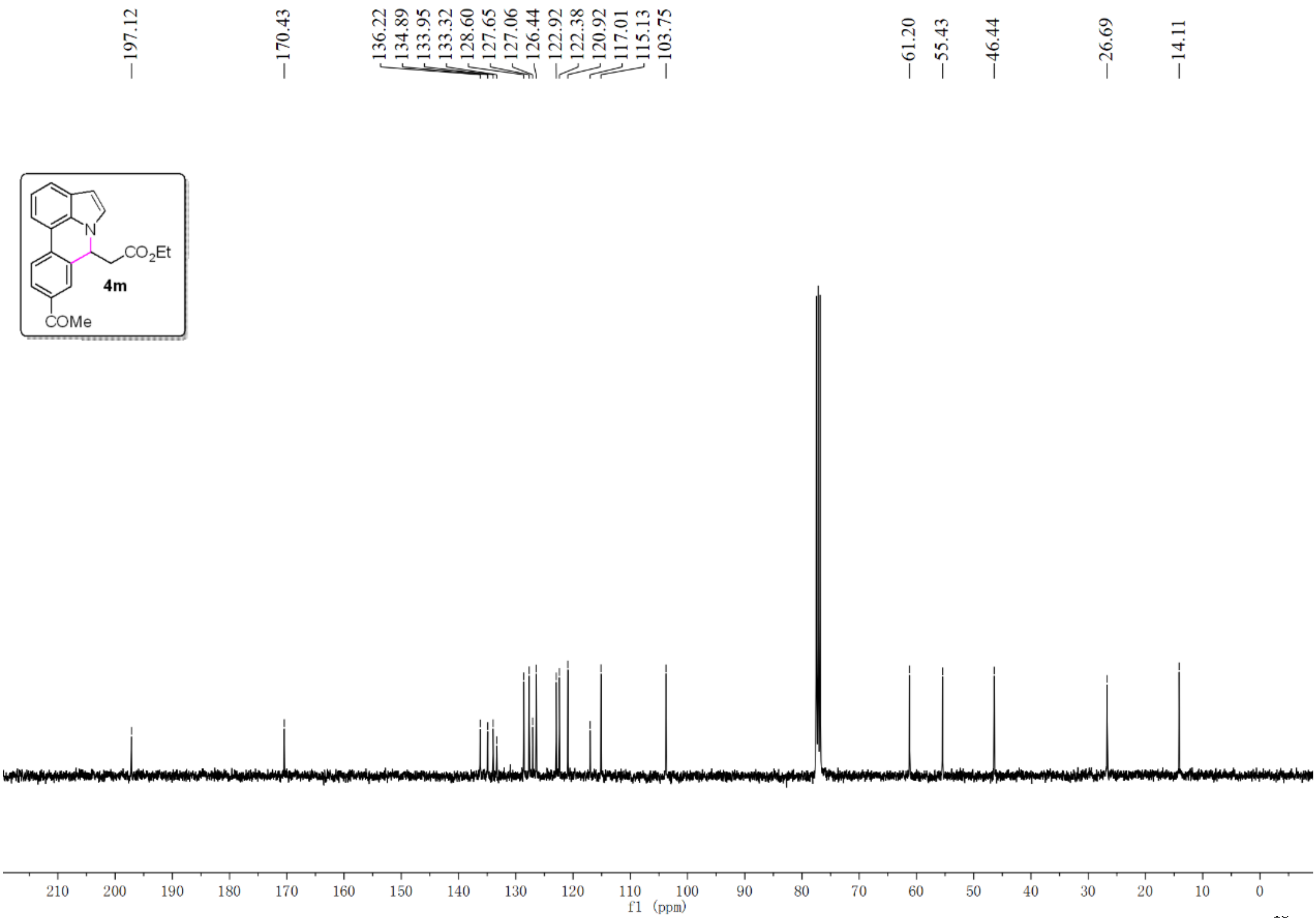

(16) The ^{1}H NMR spectrum for **4n**

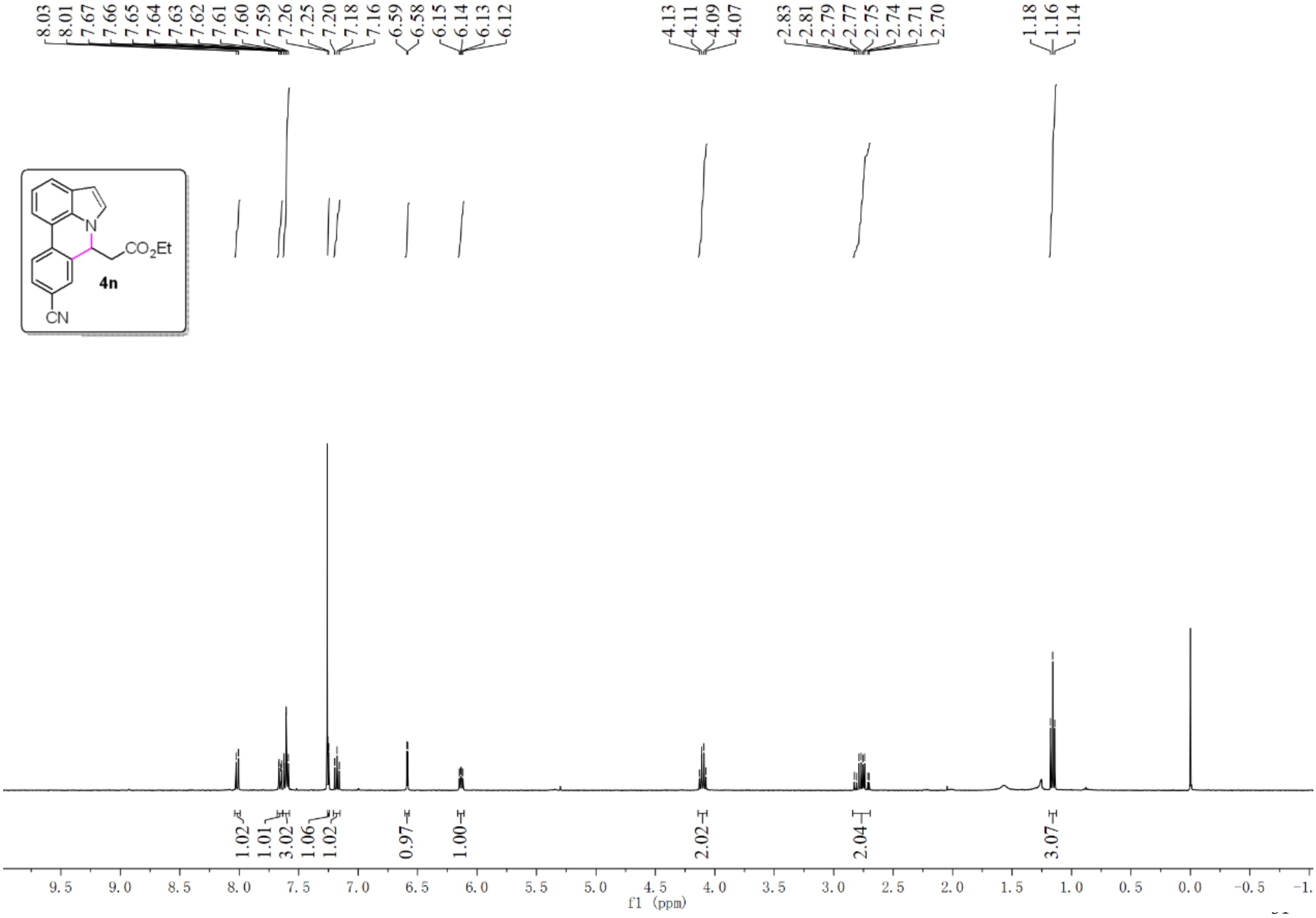

(17) The ^{1}H NMR spectrum for **4o**

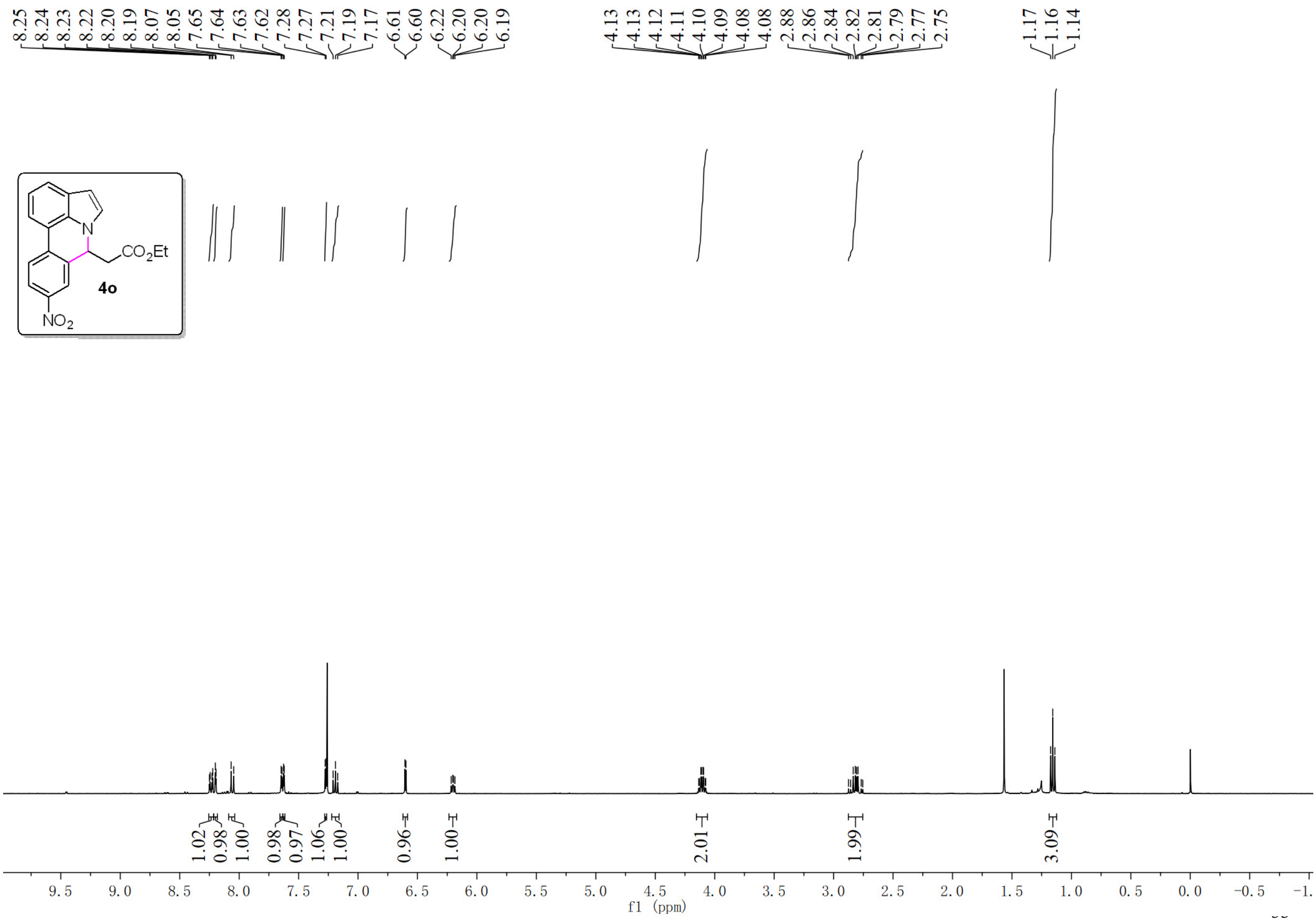

(18) The ^{1}H NMR spectrum for **4p**

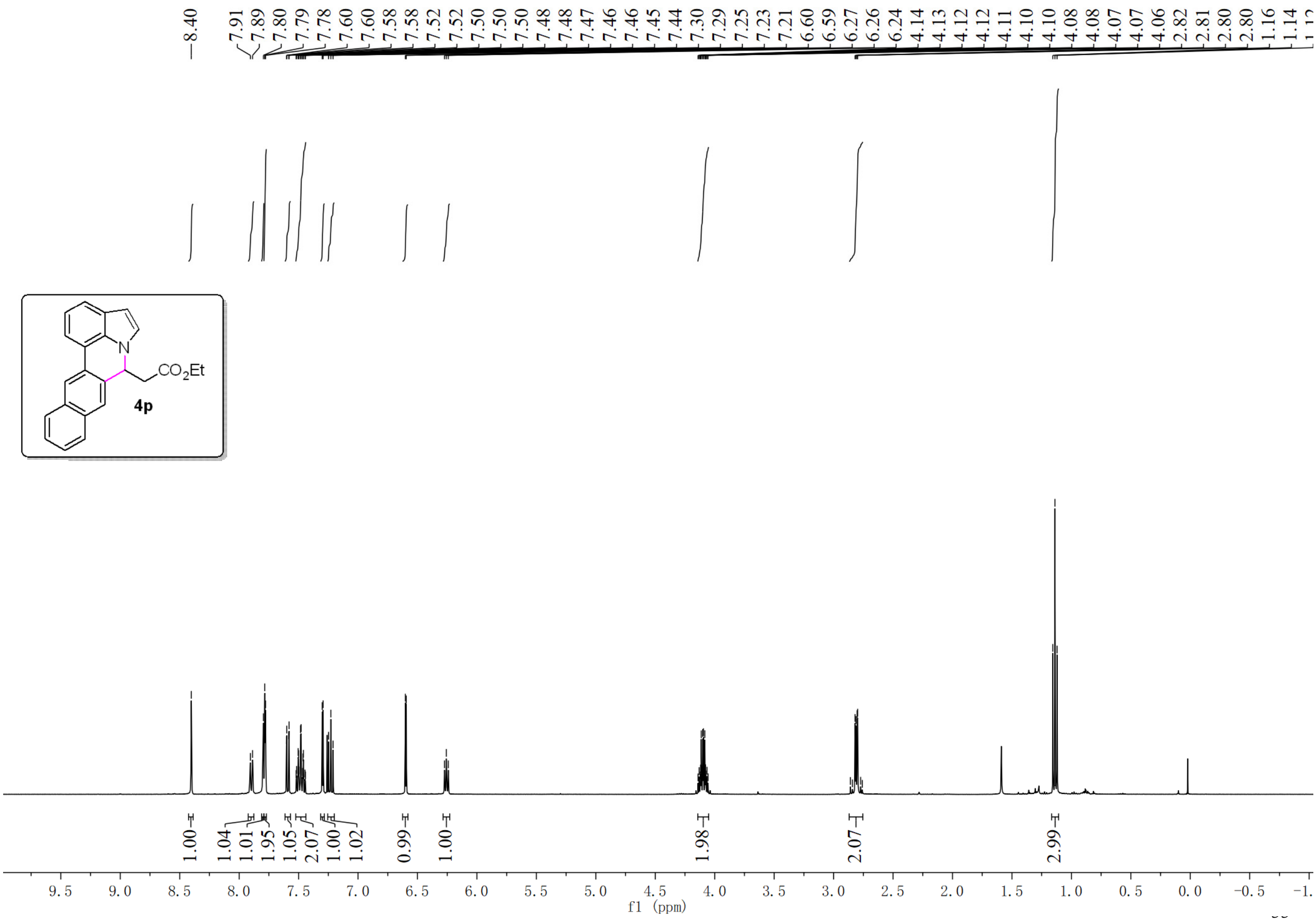

(19) The ^{1}H NMR spectrum for **4q**

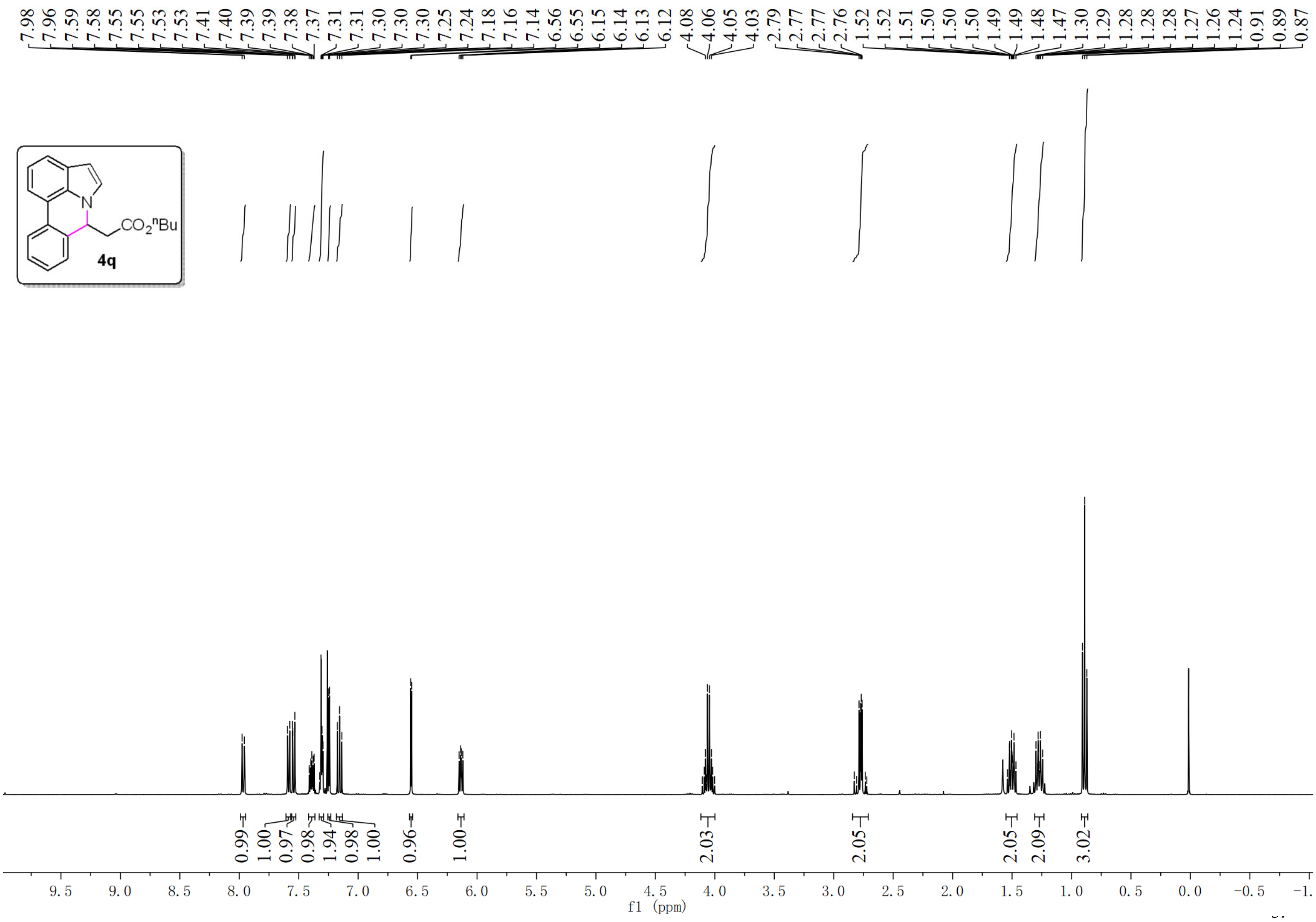

(20) The ^{1}H NMR and ^{13}C NMR spectrum for **6a** 58

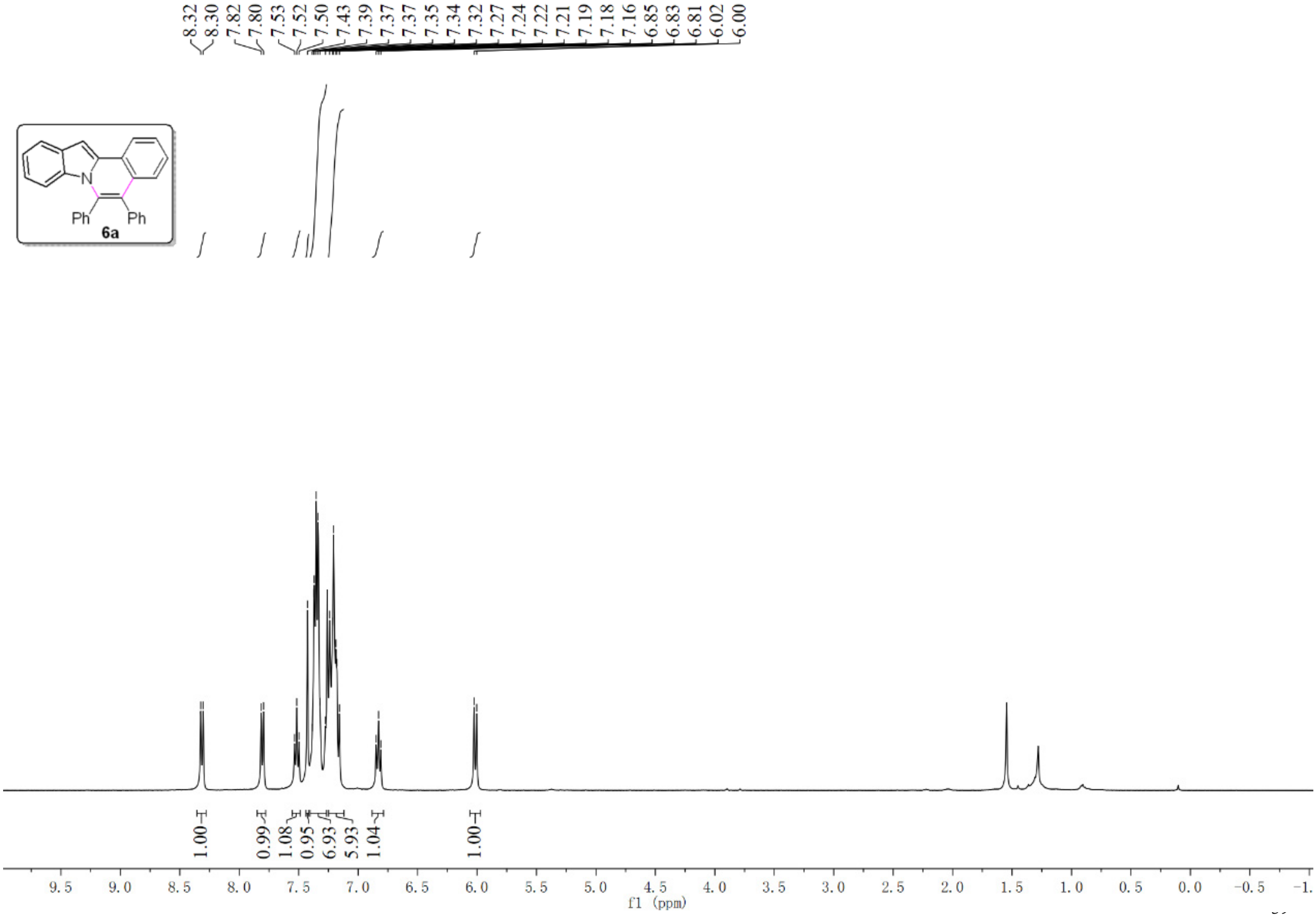
8.32
8.30
7.82
7.80
7.53
7.52
7.50
7.43
7.39
7.37
7.37
7.35
7.34
7.32
7.27
7.24
7.22
7.21
7.19
7.18
7.16
6.85
6.83
6.81
6.02
6.00
N
Ph
Ph
6a
1.00
0.99
1.08
0.95
6.93
5.93
1.04
1.00
9.5 9.0 8.5 8.0 7.5 7.0 6.5 6.0 5.5 5.0 4.5 4.0 3.5 3.0 2.5 2.0 1.5 1.0 0.5 0.0 −0.5
f1 (ppm)

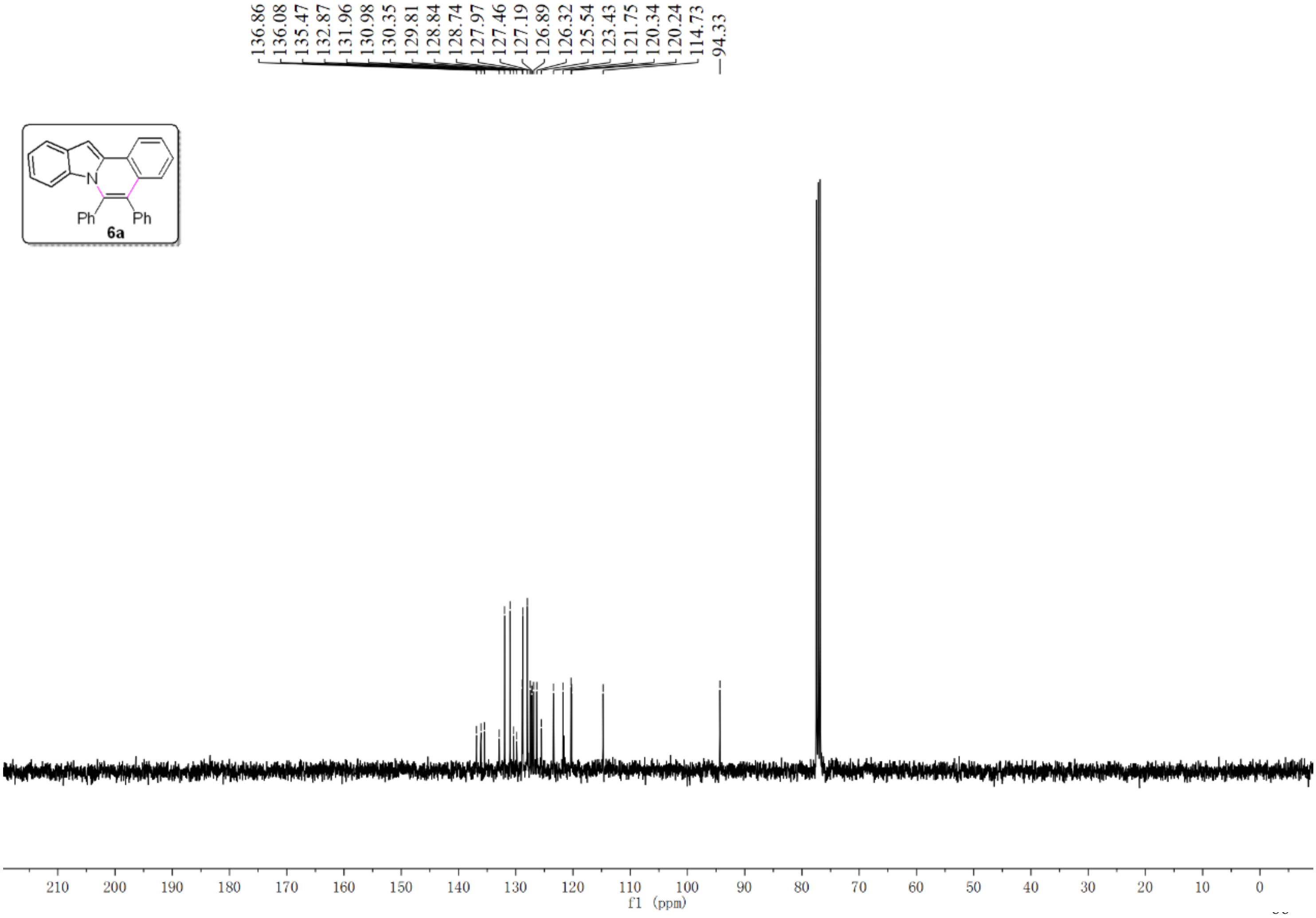

(21) The ^{1}H NMR spectrum for **6b**

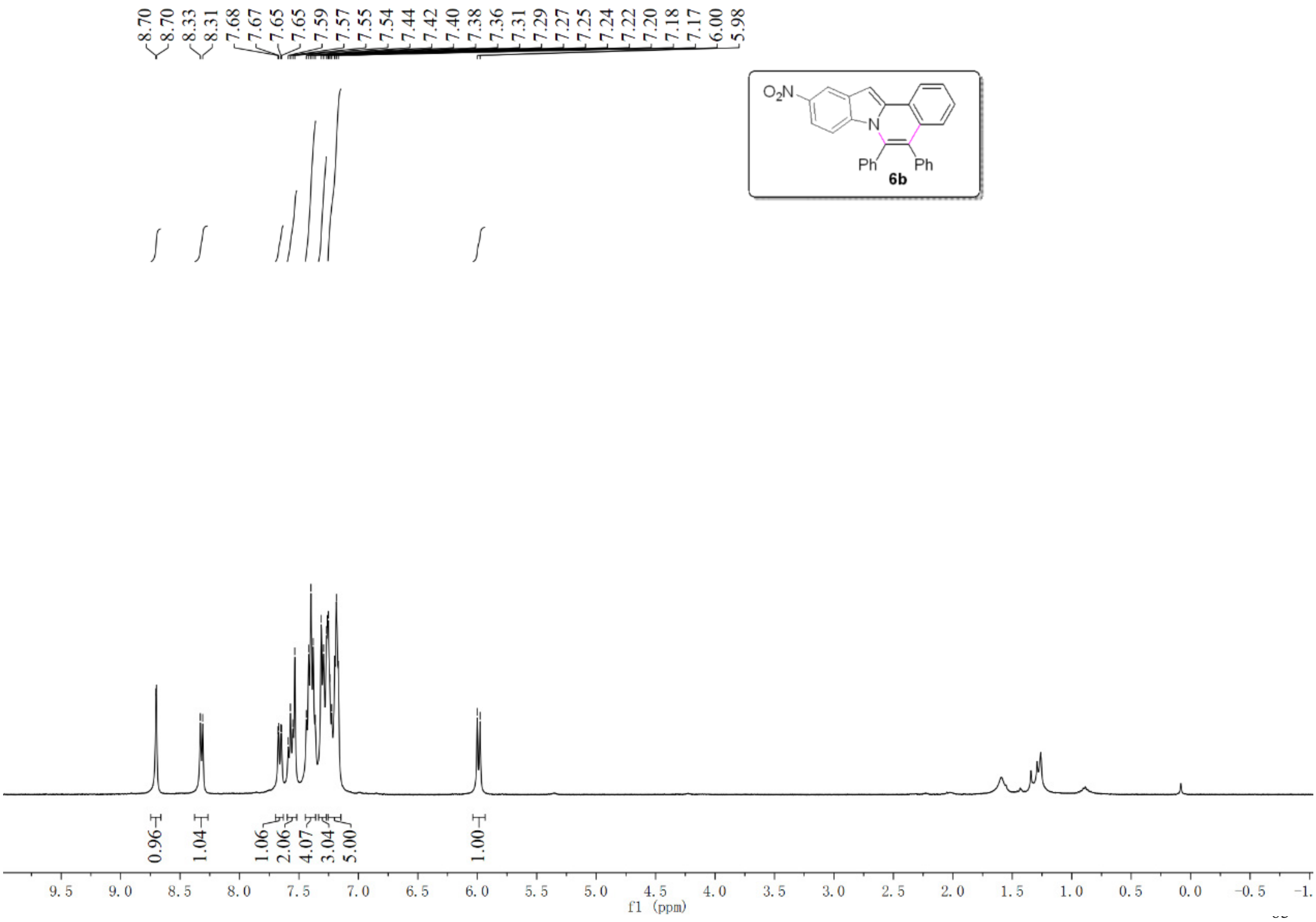

(22) The ^{1}H NMR and ^{13}C NMR spectrum for **6c**

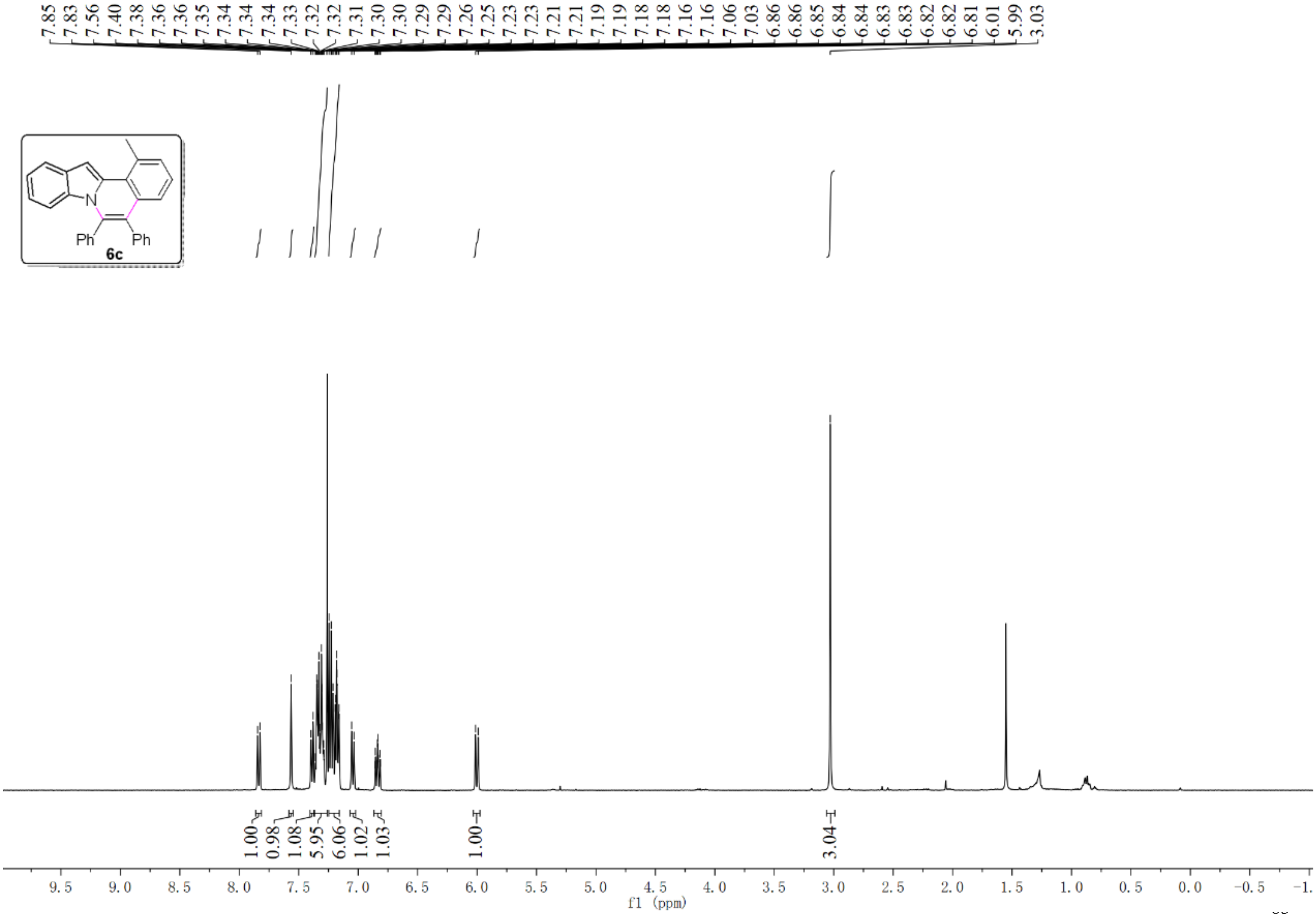
7.85
7.83
7.56
7.40
7.38
7.36
7.36
7.35
7.34
7.34
7.34
7.33
7.32
7.32
7.31
7.30
7.30
7.29
7.29
7.26
7.25
7.23
7.23
7.21
7.21
7.19
7.19
7.18
7.18
7.16
7.16
7.06
7.03
6.86
6.86
6.85
6.84
6.84
6.83
6.83
6.82
6.82
6.81
6.01
5.99
3.03
N
Ph
Ph
6c
1.00
0.98
1.08
5.95
6.06
1.02
1.03
1.00
3.04
9.5 9.0 8.5 8.0 7.5 7.0 6.5 6.0 5.5 5.0 4.5 4.0 3.5 3.0 2.5 2.0 1.5 1.0 0.5 0.0 −0.5
f1 (ppm)

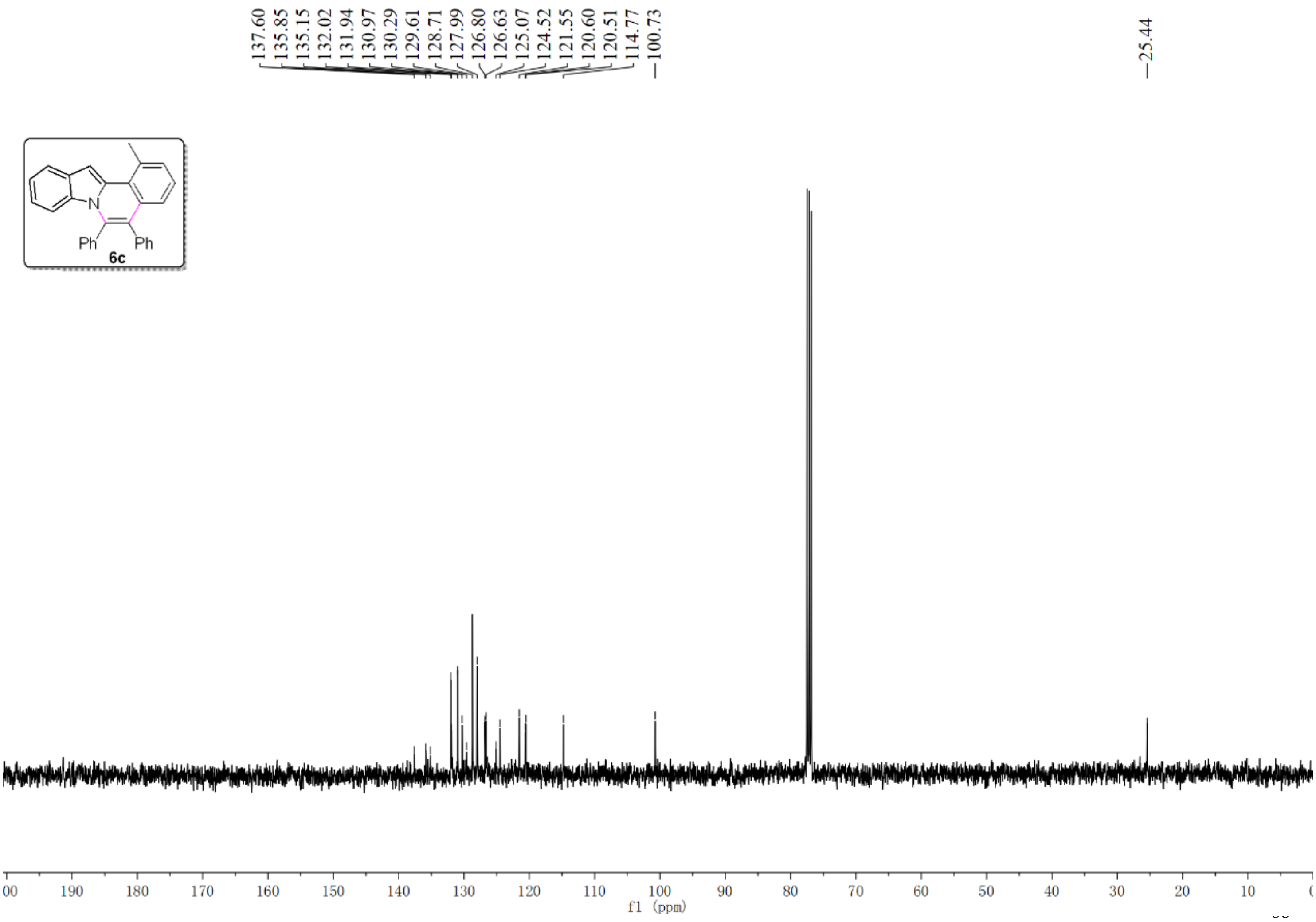

(23) The ^{1}H NMR and ^{13}C NMR spectrum for **6d**

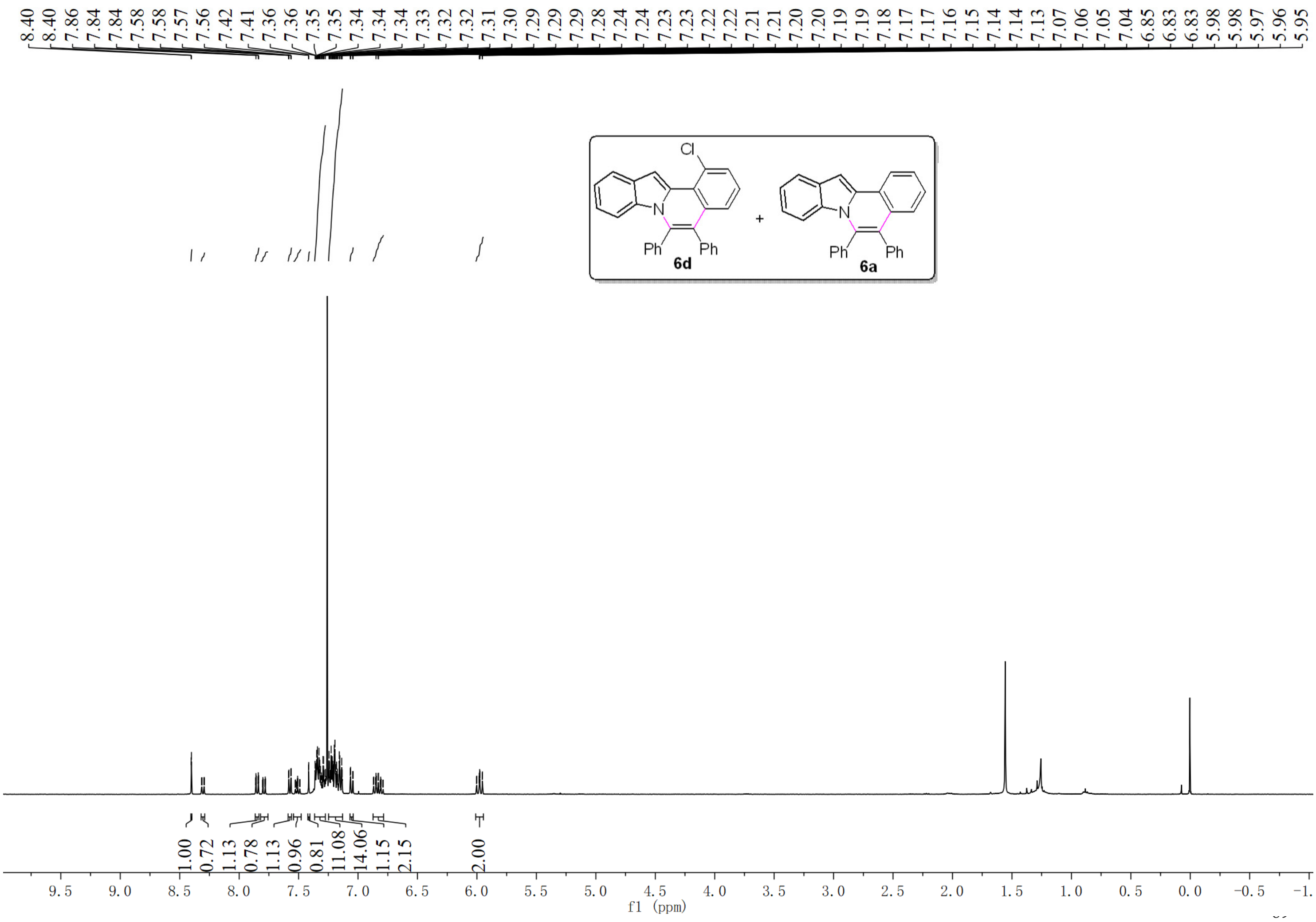

8.40
8.40
7.86
7.84
7.84
7.58
7.58
7.57
7.56
7.42
7.41
7.36
7.36
7.35
7.35
7.34
7.34
7.34
7.33
7.32
7.32
7.31
7.30
7.29
7.29
7.29
7.28
7.24
7.24
7.23
7.23
7.22
7.22
7.21
7.21
7.20
7.20
7.19
7.19
7.18
7.17
7.17
7.16
7.15
7.14
7.14
7.13
7.07
7.06
7.05
7.04
6.85
6.83
6.83
5.98
5.98
5.97
5.96
5.95
Cl
N
Ph
Ph
6d
+
N
Ph
Ph
6a
1.00
0.72
1.13
0.78
1.13
0.96
0.81
11.08
14.06
1.15
2.15
2.00
9.5
9.0
8.5
8.0
7.5
7.0
6.5
6.0
5.5
5.0
4.5
4.0
3.5
3.0
2.5
2.0
1.5
1.0
0.5
0.0
−0.5
f1 (ppm)

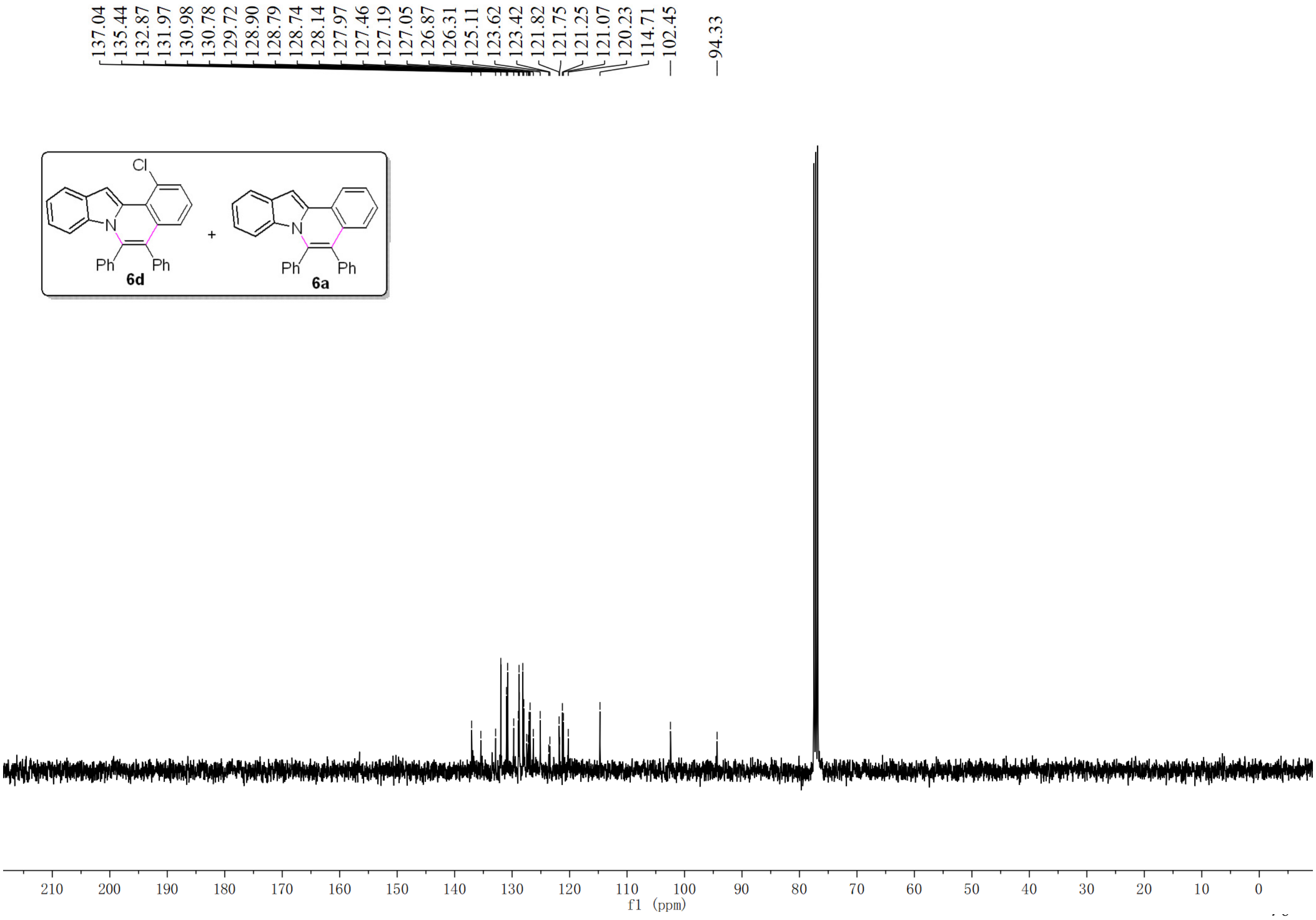

(24) The ^{1}H NMR and ^{13}C NMR spectrum for **6e**

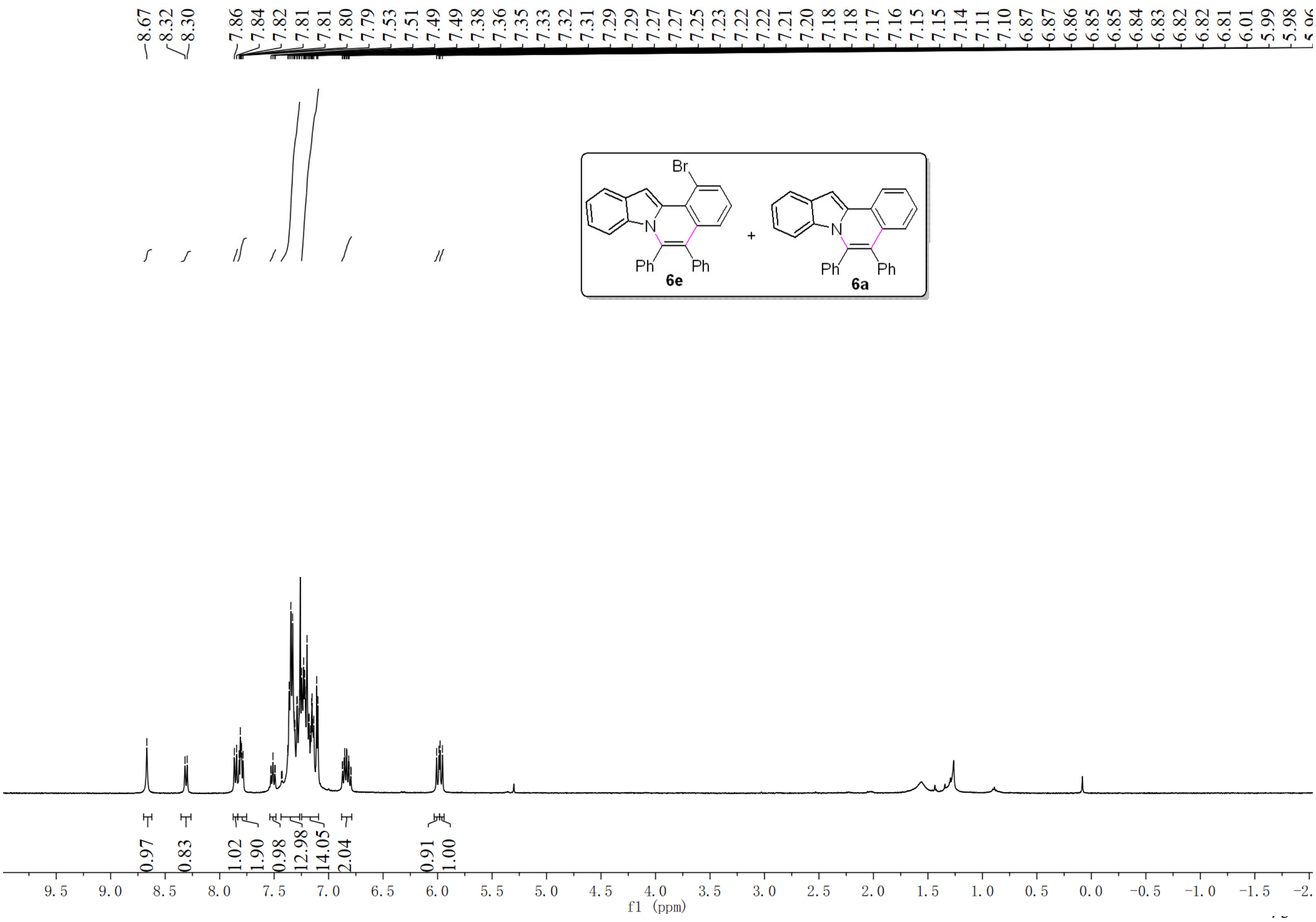
8.67
8.32
8.30
7.86
7.84
7.82
7.81
7.81
7.80
7.79
7.53
7.51
7.49
7.49
7.38
7.36
7.35
7.33
7.32
7.31
7.29
7.29
7.27
7.27
7.25
7.23
7.22
7.22
7.21
7.20
7.18
7.18
7.17
7.16
7.15
7.15
7.14
7.11
7.10
6.87
6.87
6.86
6.85
6.85
6.84
6.83
6.82
6.82
6.81
6.01
5.99
5.98
Br
N
Ph
Ph
6e
+
N
Ph
Ph
6a
0.97
0.83
1.02
1.90
0.98
12.98
14.05
2.04
0.91
1.00
9.5
9.0
8.5
8.0
7.5
7.0
6.5
6.0
5.5
5.0
4.5
4.0
3.5
3.0
2.5
2.0
1.5
1.0
0.5
0.0
−0.5
−1.0
−1.5
f1 (ppm)

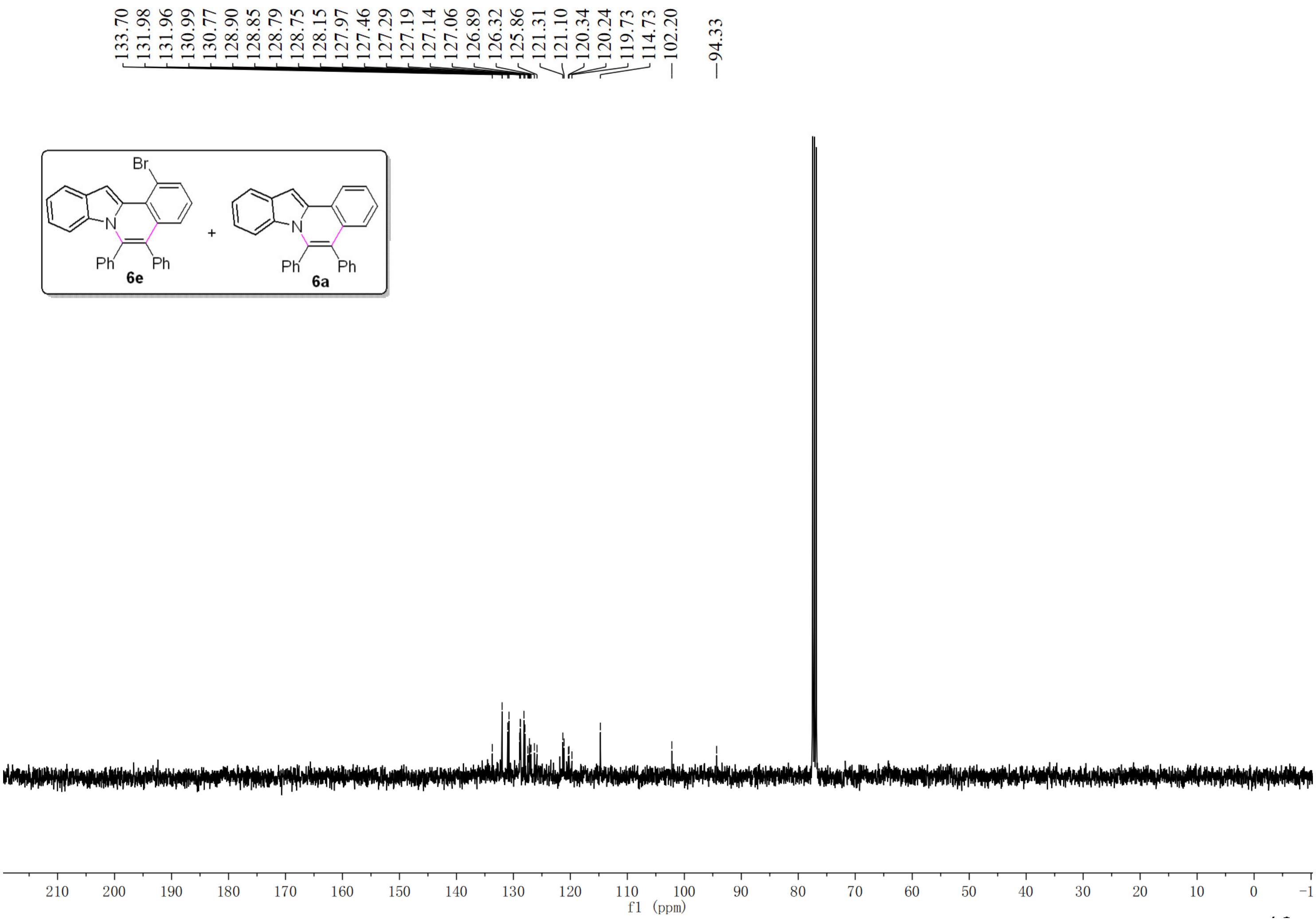

(25) The ^{1}H NMR and ^{13}C NMR spectrum for **6f**

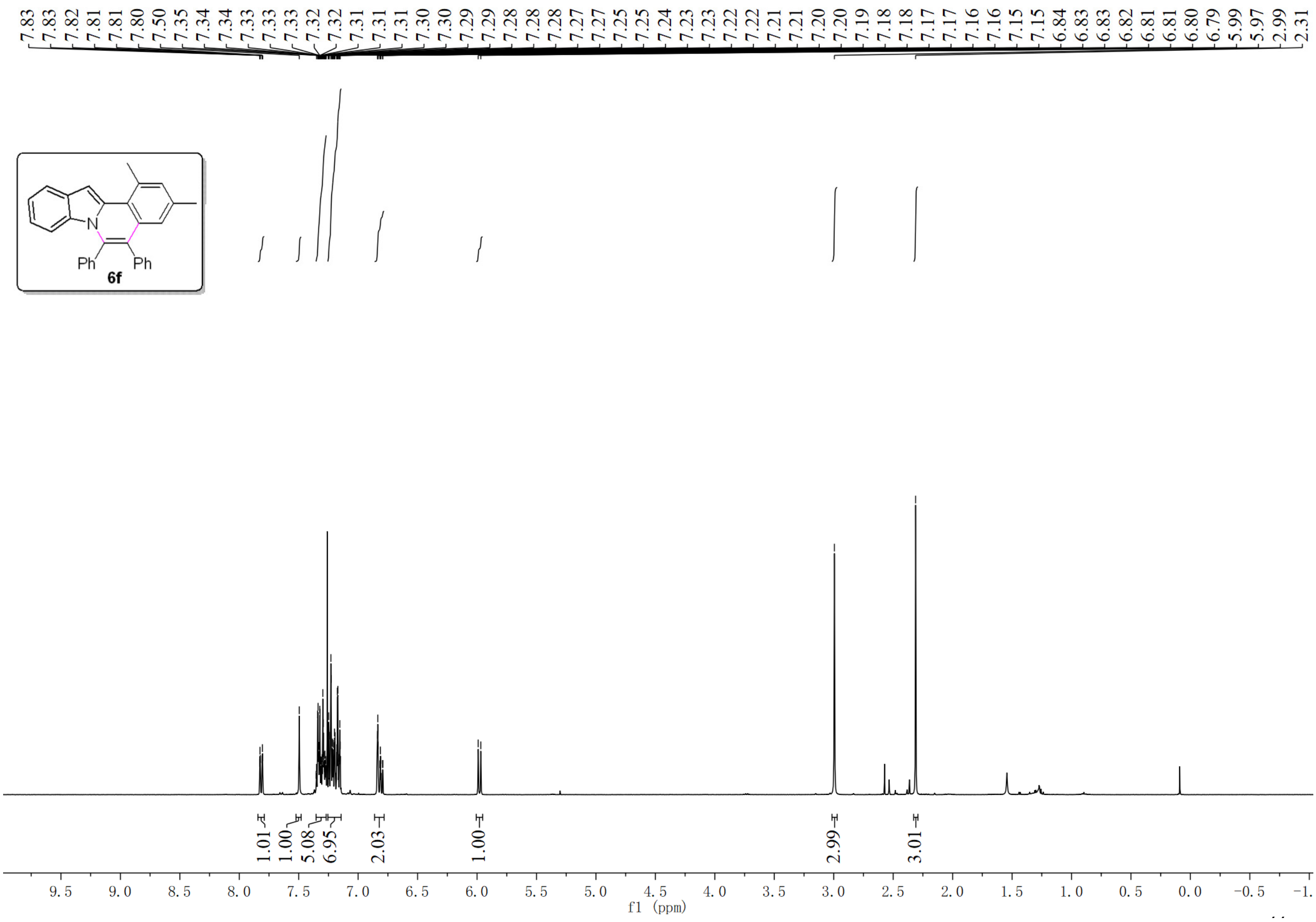
7.83
7.83
7.82
7.81
7.81
7.80
7.50
7.35
7.34
7.34
7.33
7.33
7.33
7.32
7.32
7.31
7.31
7.31
7.30
7.30
7.29
7.29
7.28
7.28
7.28
7.27
7.27
7.25
7.25
7.24
7.23
7.23
7.22
7.22
7.21
7.21
7.20
7.20
7.19
7.18
7.18
7.17
7.17
7.16
7.16
7.15
7.15
6.84
6.83
6.83
6.82
6.81
6.81
6.80
6.79
5.99
5.97
2.99
2.31
N
Ph
Ph
6f
1.01
1.00
5.08
6.95
2.03
1.00
2.99
3.01
9.5
9.0
8.5
8.0
7.5
7.0
6.5
6.0
5.5
5.0
4.5
4.0
3.5
3.0
2.5
2.0
1.5
1.0
0.5
0.0
-0.5
f1 (ppm)

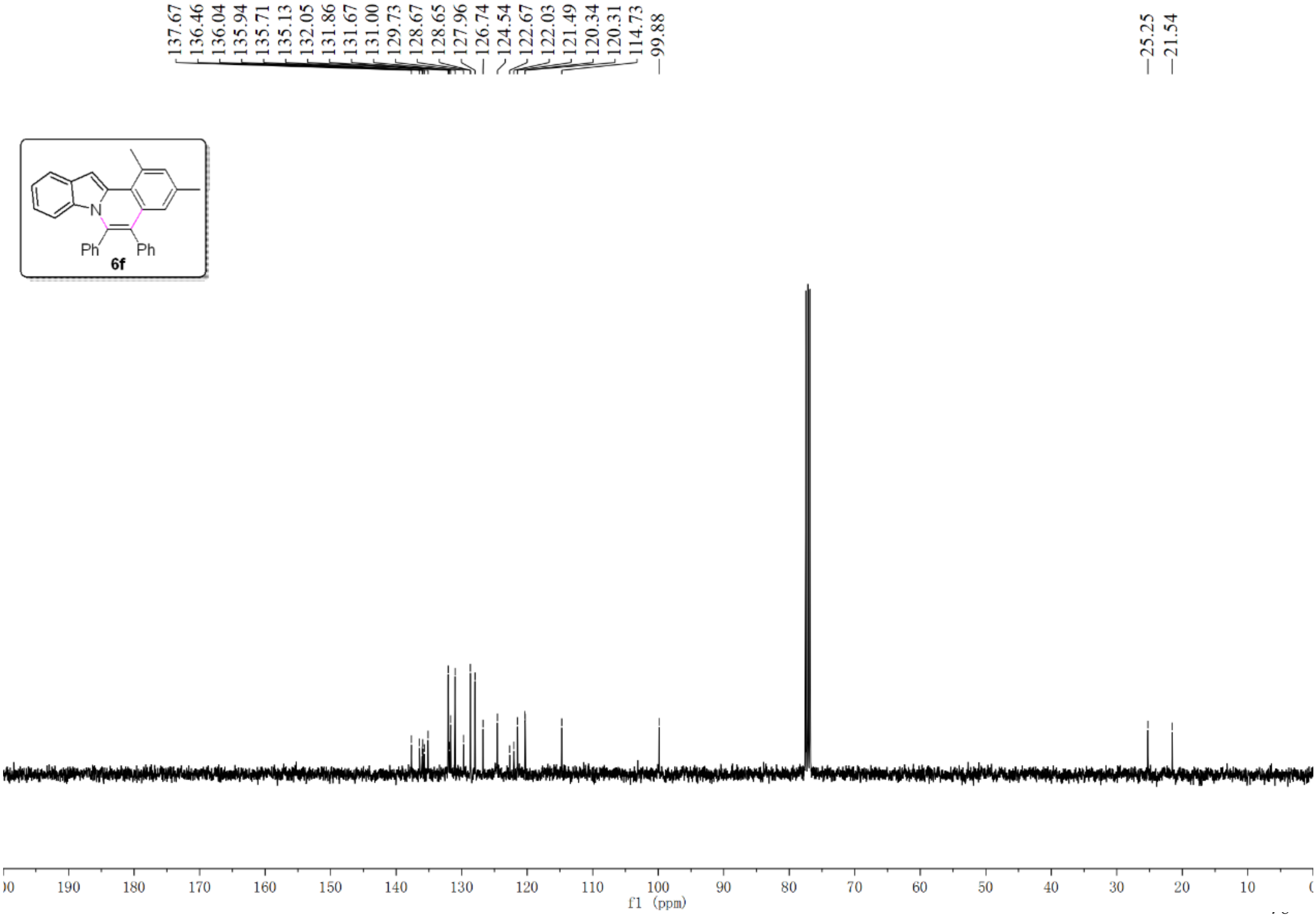

(26) The ^{1}H NMR and ^{13}C NMR spectrum for **6g**

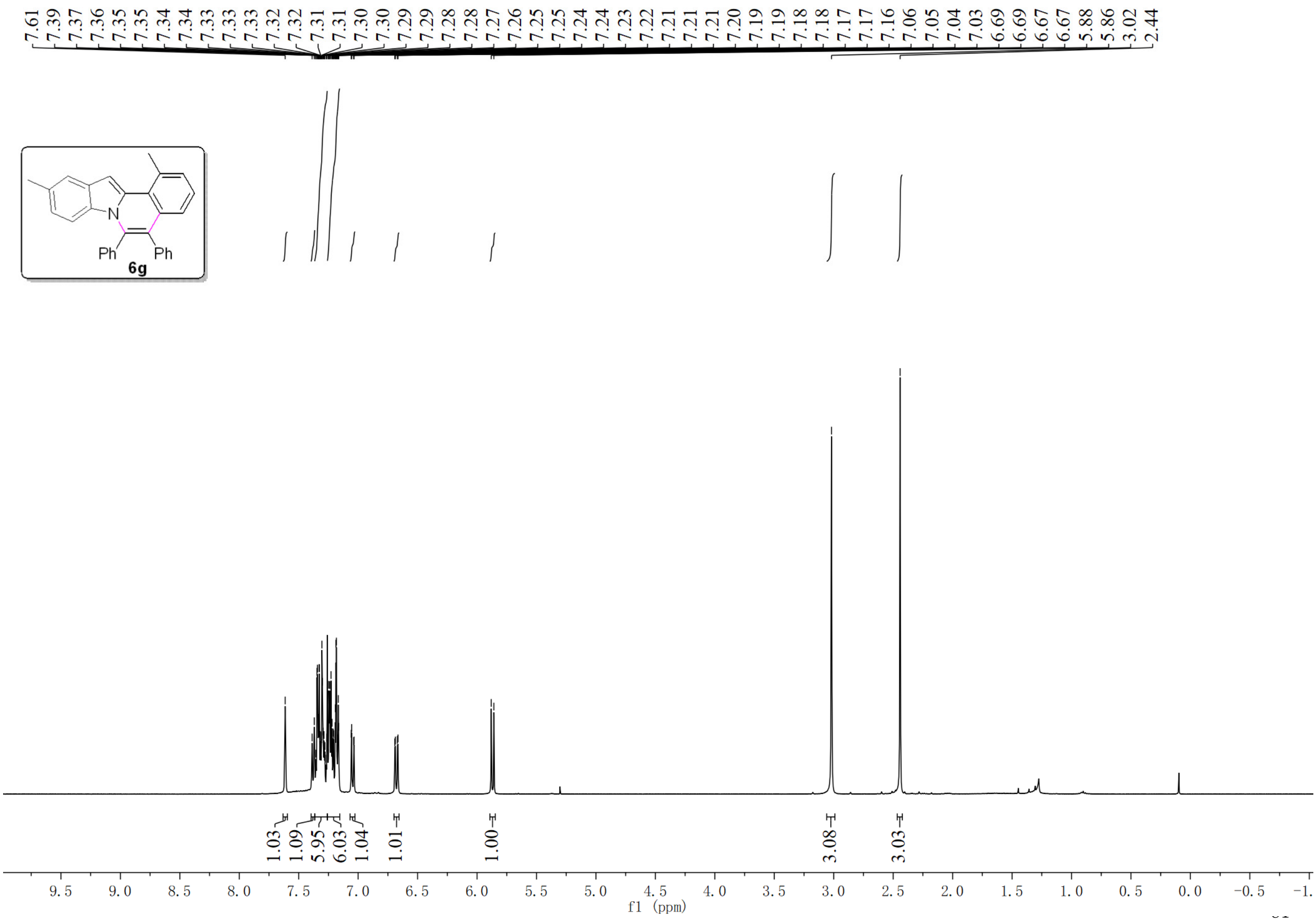
7.61 7.39 7.37 7.36 7.35 7.35 7.34 7.34 7.33 7.33 7.33 7.32 7.32 7.31 7.31 7.30 7.30 7.29 7.29 7.28 7.28 7.27 7.26 7.25 7.25 7.24 7.24 7.23 7.22 7.21 7.21 7.21 7.20 7.19 7.19 7.18 7.18 7.17 7.17 7.16 7.06 7.05 7.04 7.03 6.69 6.69 6.67 6.67 5.88 5.86 3.02 2.44
Ph
Ph
6g
1.03 1.09 5.95 6.03 1.04 1.01 1.00 3.08 3.03
9.5 9.0 8.5 8.0 7.5 7.0 6.5 6.0 5.5 5.0 4.5 4.0 3.5 3.0 2.5 2.0 1.5 1.0 0.5 0.0 −0.5
f1 (ppm)

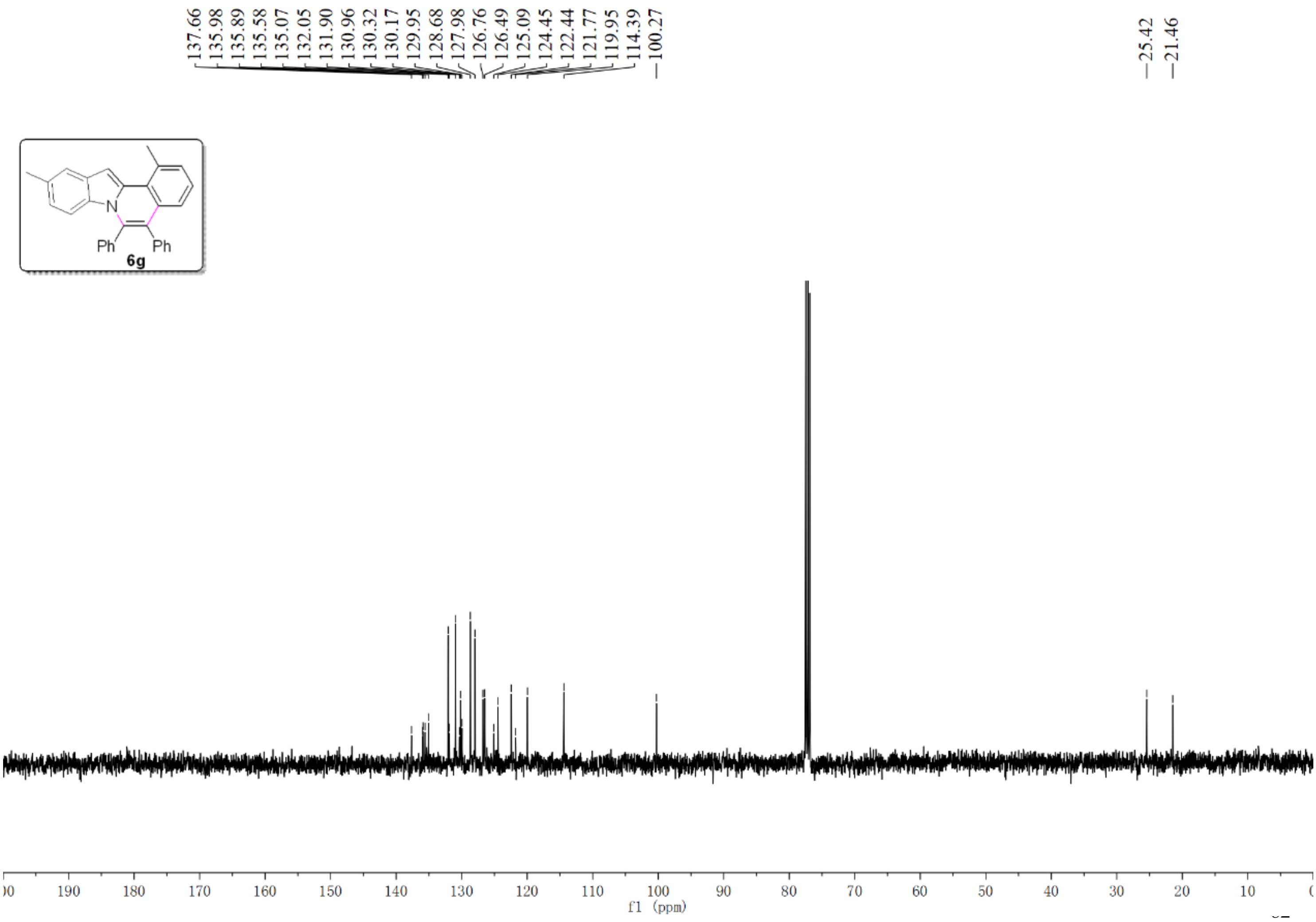

(27) The ^{1}H NMR and ^{13}C NMR spectrum for **6h**

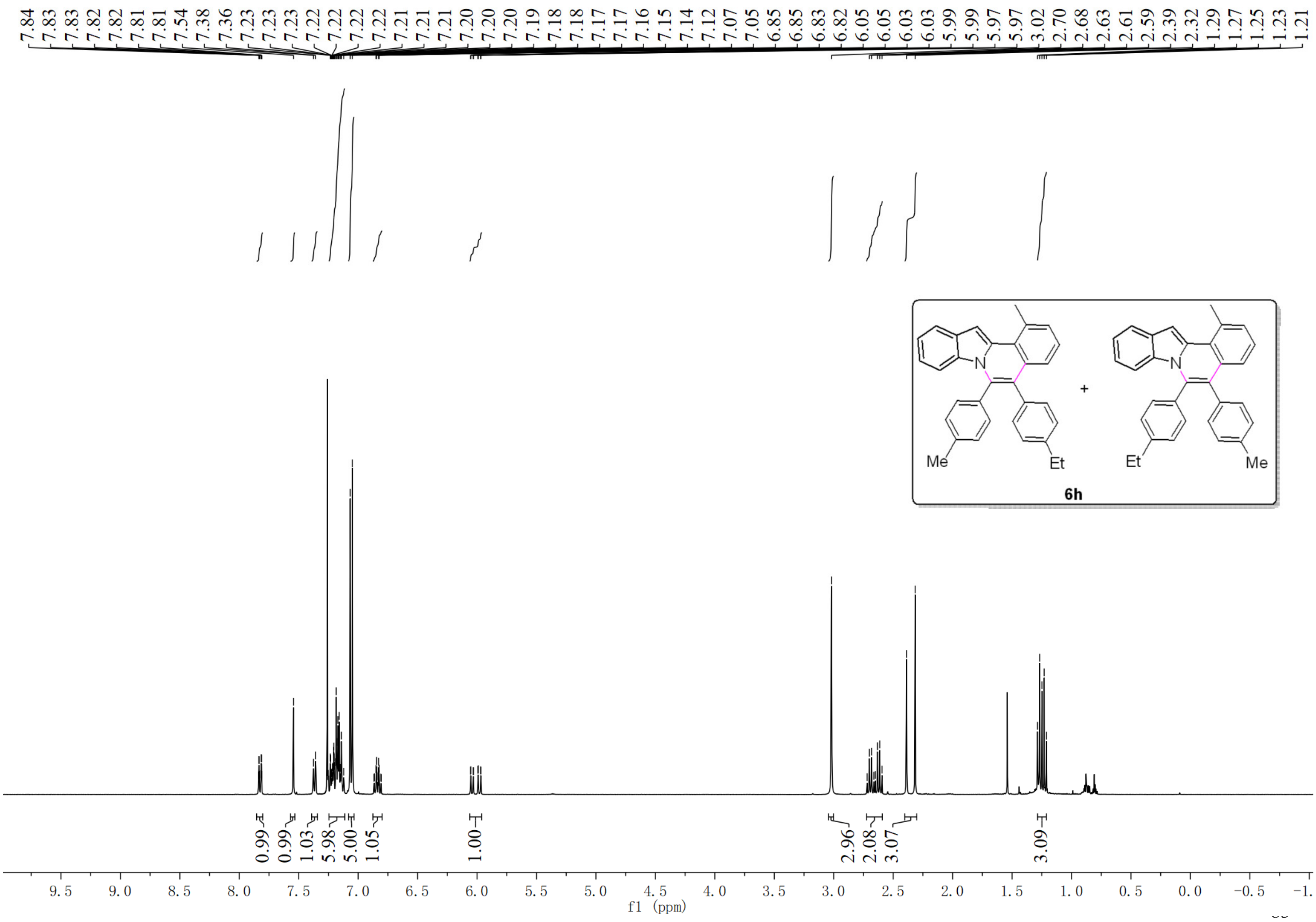
6h
Me
Et
+
N
f1 (ppm)

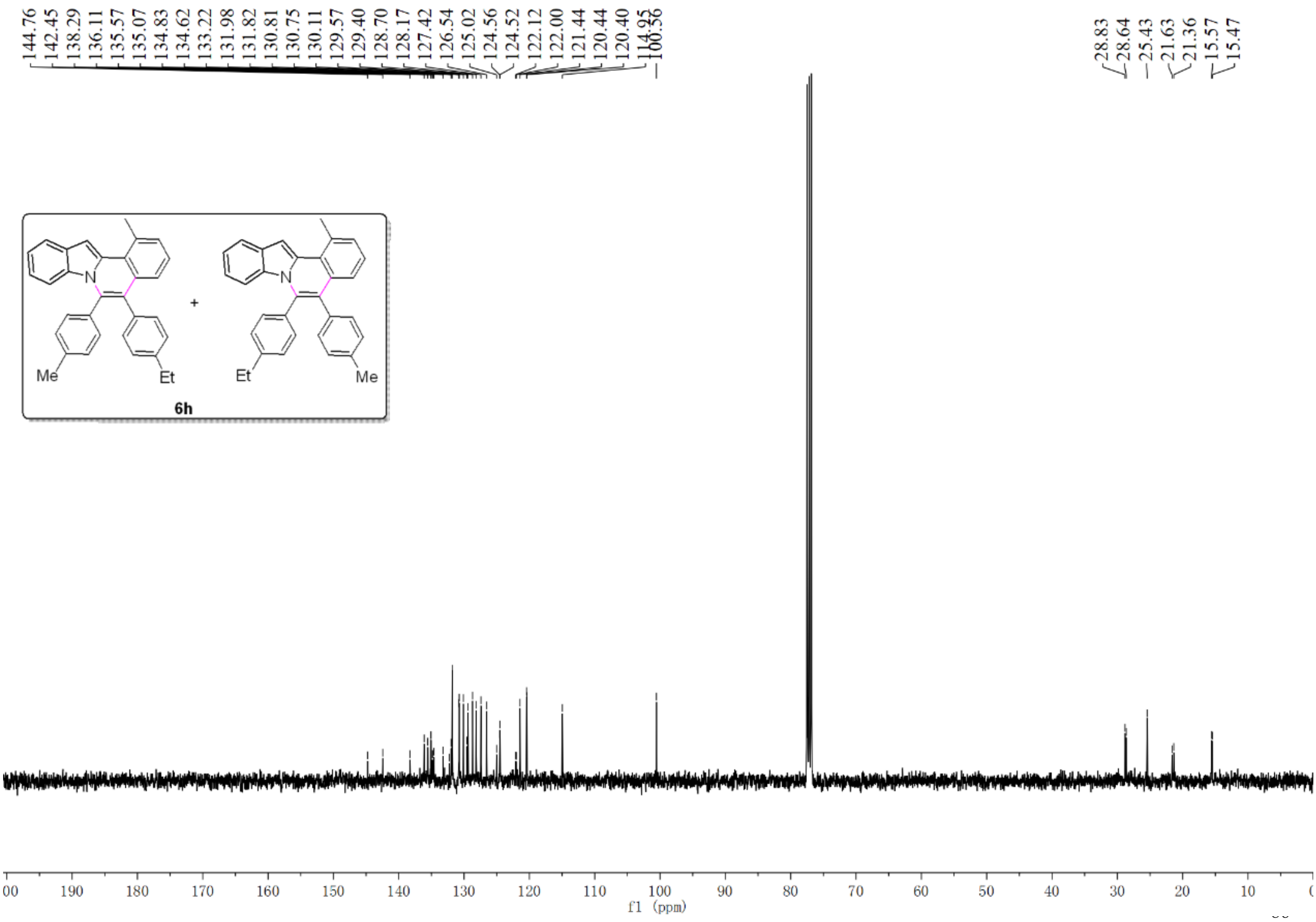
144.76
142.45
138.29
136.11
135.57
135.07
134.83
134.62
133.22
131.98
131.82
130.81
130.75
130.11
129.57
129.40
128.70
128.17
127.42
126.54
125.02
124.56
124.52
122.12
122.00
121.44
120.44
120.40
114.95
100.56
28.83
28.64
25.43
21.63
21.36
15.57
15.47
Me
Et
+
Et
Me
6h
f1 (ppm)
